# Catalytic site inhibition of insulin-degrading enzyme by a small molecule induces glucose intolerance in mice

**DOI:** 10.1038/ncomms9250

**Published:** 2015-09-23

**Authors:** Rebecca Deprez-Poulain, Nathalie Hennuyer, Damien Bosc, Wenguang G. Liang, Emmanuelle Enée, Xavier Marechal, Julie Charton, Jane Totobenazara, Gonzague Berte, Jouda Jahklal, Tristan Verdelet, Julie Dumont, Sandrine Dassonneville, Eloise Woitrain, Marion Gauriot, Charlotte Paquet, Isabelle Duplan, Paul Hermant, François- Xavier Cantrelle, Emmanuel Sevin, Maxime Culot, Valerie Landry, Adrien Herledan, Catherine Piveteau, Guy Lippens, Florence Leroux, Wei-Jen Tang, Peter van Endert, Bart Staels, Benoit Deprez

**Affiliations:** 1 Institut Pasteur de Lille, Lille F-59000, France; 2 Université de Lille, Institut Federatif de Recherche 114, Lille F-59000, France; 3 Institut National de la Sante et de la Recherche Medicale, U1177 Drugs and Molecules for Living Systems, Lille F-59000, France; 4 Institut Federatif de Recherche 142, Molecular and Cellular Medicine, Lille F-59000, France; 5 Pole de Recherche Interdisciplinaire pour le Medicament, Lille F-59000, France; 6 Institut National de la Sante et de la Recherche Medicale, U1011 Nuclear Receptors, Cardiovascular Diseases and Diabetes, European Genomic Institute for Diabetes, Lille F-59000, France; 7 Ben-May Institute for Cancer Research, The University of Chicago, Chicago, Illinois 60637, USA; 8 Institut National de la Sante et de la Recherche Medicale, Unité 1151; Université Paris Descartes, Sorbonne Paris Cité; Centre National de la Recherche Scientifique, , Unité 8253, Paris, France; 9 Centre National de la Recherche Scientifique, UMR 8576, Structural and Functional Glycobiology, Université de Lille, Lille F-59000, France; 10 Université d'Artois, LBHE, EA2465, Lens F-62300, France

## Abstract

Insulin-degrading enzyme (IDE) is a protease that cleaves insulin and other bioactive peptides such as amyloid-β. Knockout and genetic studies have linked IDE to Alzheimer’s disease and type-2 diabetes. As the major insulin-degrading protease, IDE is a candidate drug target in diabetes. Here we have used kinetic target-guided synthesis to design the first catalytic site inhibitor of IDE suitable for *in vivo* studies (BDM44768). Crystallographic and small angle X-ray scattering analyses show that it locks IDE in a closed conformation. Among a panel of metalloproteases, BDM44768 selectively inhibits IDE. Acute treatment of mice with BDM44768 increases insulin signalling and surprisingly impairs glucose tolerance in an IDE-dependent manner. These results confirm that IDE is involved in pathways that modulate short-term glucose homeostasis, but casts doubt on the general usefulness of the inhibition of IDE catalytic activity to treat diabetes.

## Abstract (web summary)

Inhibiting insulin-degrading enzyme (IDE) has been proposed as a potential therapeutic strategy for the treatment of patients with diabetes. Here, the authors develop a novel IDE inhibitor but find that, surprisingly, IDE inhibition has negative effects on glucose tolerance in mice.

## Graphical abstract

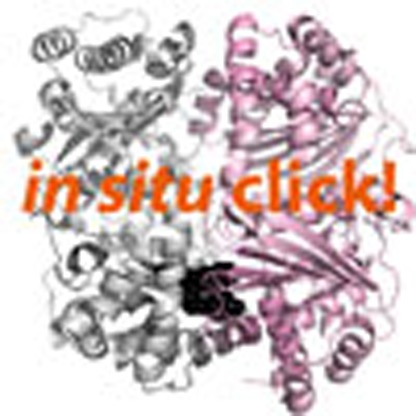

Insulin-degrading enzyme (IDE) is a 110 kDa zinc protease of the M16 family that is highly conserved and involved in the degradation of insulin^1^,^2^, amyloid-β (Aβ) (ref. 3), IGF-II (ref. 4), glucagon^5^, amylin^6^ and somatostatin^7^. Interestingly, although these substrates have unrelated amino-acid sequences, many of them are amyloidogenic^8^. The structure of IDE is atypical^9^ with a very large catalytic chamber, called ‘crypt’, formed by two joining N- and C-terminal domains (Fig. 1a)^10^. The zinc ion is located in the N-terminal domain, but key residues forming the hydrolytic site are located in both domains, resulting in constitution of the catalytic site only in the closed state. IDE has broad tissue distribution and subcellular localization, and a small fraction of IDE is secreted^11^. IDE acts not only through proteolysis but also via interactions with other intracellular proteins^12^,^13^,^14^ including chaperone-like activity on amyloidogenic peptides^15^. Mirsky and Perisutti showed that a crudely prepared liver-derived IDE inhibitor could enhance the hypoglycaemia action of insulin^16^, suggesting a therapeutic potential of IDE-targeted drugs. Subsequently, Fakhrai-Rad *et al.* showed that Goto-Kakizaki rats, which exhibit non-obese type-2 diabetes^17^, differ from the Wistar parent strain by an *Ide* allele coding for an enzyme with reduced activity. This observation prompted the suggestion that hypofunctional IDE is linked to diabetes^18^,^19^. In 2003, Farris *et al.*^20^ reported that *Ide* knockout mice display hyperinsulinemia, glucose intolerance and increased cerebral accumulation of endogenous Aβ. Abdul-Hay *et al.*^21^ more recently confirmed the observation of glucose intolerance, which however was limited to older mice. Both authors interpreted the glucose intolerance as a consequence of chronically elevated serum insulin levels. The observation of glucose intolerance contrasted with an earlier suggestion put forth by Mirsky and Perisutti in 1955 that transient IDE inhibition, by increasing circulating insulin levels, may be suitable as a treatment of diabetes. Other authors have also hypothesized that acute pharmacological inhibition of IDE may be a treatment option for diabetes^22^.

To test this hypothesis, specific inhibitors suitable for *in vivo* experiments are required. Potent substrate-based inhibitors of IDE have previously been described in the literature^22^,^23^. However, knowledge concerning the efficacy of these peptidic probes was limited to *in vitro* assays testing the degradation of exogenously added insulin by CHO cells overexpressing the human insulin receptor. Information on the consequence of inhibition of intracellular IDE was not obtained. None of these inhibitors could be tested *in vivo* owing to poor pharmacokinetic properties. Very recently, Maianti *et al.*^24^ have discovered non-catalytic site inhibitors of IDE. They have shown that inhibition of IDE through binding to a distal site of the enzyme modulates glucose tolerance in mice and discussed the applicability of IDE inhibitors to treat diabetes. Here, we report the development of a potent catalytic site inhibitor suitable for *in vivo* administration, and the characterization of its short-term *in vivo* effects on glucose tolerance in rodents.

## Results

### Discovery of inhibitors using kinetic target-guided synthesis

We designed an orthogonal multicomponent kinetic target-guided synthesis (TGS) experiment that allowed us to discover new IDE inhibitors with improved properties. In kinetic TGS^25^, the protein target is used to synthesize a divalent inhibitor by equilibrium-controlled selection of reagents with complementary reactive functions until an irreversible reaction links the pair of reagents that best fits the protein binding site. Only a few chemical reactions are amenable to kinetic TGS. The Huisgen cycloaddition involving one azide and one alkyne to form a disubstituted triazole is the most popular. This kind of TGS was pioneered by Sharpless and collaborators and has been shown to be useful in the search for active compounds in medicinal chemistry^26^. Several inhibitors of enzymes have been discovered in this way. They include inhibitors of acetylcholine esterase^26^,^27^, carbonic anhydrase^28^, HIV protease^29^ and chitinase^30^. TGS was also used to discover receptor antagonists^31^.

**Figure 1:**
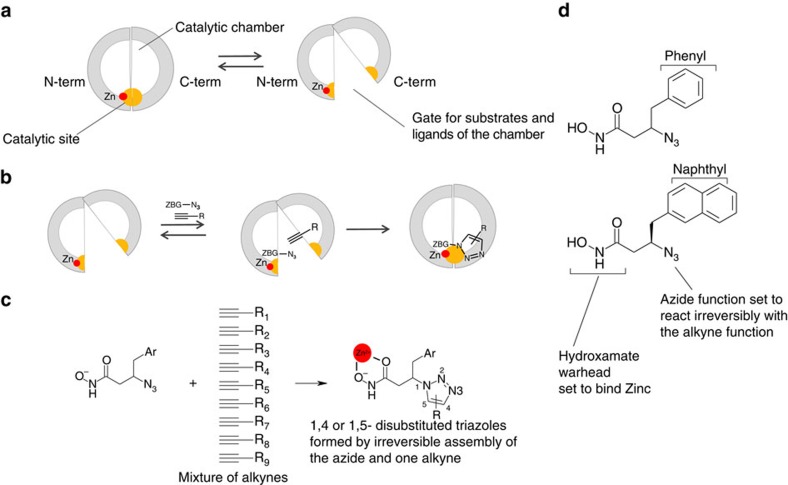
Use of TGS to design catalytic site inhibtors of IDE. (**a**) Schematic view of IDE showing the catalytic site in yellow formed inside the catalytic chamber by the N- and C-terminal domains. The equilibrium between closed and open conformations is shown. (**b**) Principle of kinetic TGS: an azide-bearing hydroxamate warhead (ZBG-N_3_) and an alkyne are shown bound to the enzyme and reacting irreversibly to form a triazole. (**c**) IDE chooses reagents amongst a mixture of alkynes (**d**) The two azide-bearing hydroxamate warheads used in the experiment.

We successfully used kinetic TGS coupled to high-resolution mass spectrometry detection to discover inhibitors binding to the IDE conformationally flexible catalytic site (Fig. 1a,b) and guide subsequent medicinal chemistry optimisation. The experiment was performed with diverse alkynes and two azide warheads designed to bind to the catalytic zinc ion of IDE. In a second step, several triazoles formed in kinetic TGS conditions and close analogues were selected and synthesized by procedures detailed in Supplementary Methods. The structure–activity relationships obtained on two substrates of IDE *in vitro* were determined and rationalized with respect to the crystal structure of the enzyme complexed to the best inhibitor (compound **1**, BDM44768) and two analogues.

### Design and use of reagents for TGS

We used the information available on substrate preference and inhibition of human IDE (*h*IDE) to design two azide-bearing hydroxamate warheads set to bind the zinc ion in the N terminus of the enzyme^1^,^22^. Ninety diverse alkynes were selected as potential binders to the C-terminal part of the catalytic site and sorted in orthogonal clusters (Supplementary Table 1). The two hydroxamic warheads (Fig. 1d) were used individually, whereas alkynes were handled as clusters. A series of 20 9-to-1 mixtures of reagents was incubated with *h*IDE or a control buffer at 37 °C for 72 h, potentially giving rise to 360 products. Interestingly, a direct analysis of the crude reaction mixtures by liquid chromatography coupled to high-resolution electrospray time-of-flight mass spectrometry suggested key aspects of structure–activity relationships (SAR). First, all compounds formed are derived from the naphthyl azide (Supplementary Fig. 1). Second, both 1,4- and 1,5-triazoles are formed (Supplementary Fig. 1), probably as a result of the large and flexible catalytic site. Finally, regarding the substituent brought by the alkyne, the analysis of the two partitions of mixtures directly showed that (a) the presence of an amide or a sulfonamide group in the linker is beneficial and (b) the group directly attached to the alkyne is preferred to be a cycloalkyl, a phenyl group (Supplementary Fig. 1).

### Structure-activity relationships

**Figure 2:**
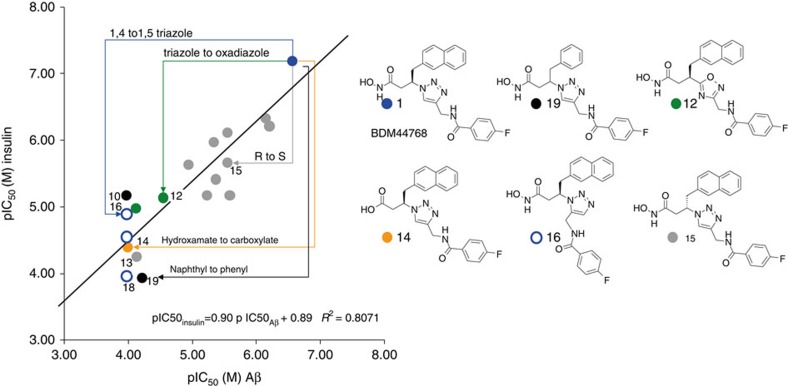
Structure–activity relationships as function of substrates. Compounds with coordinates (*x*, *y*) that show pIC_50_s measured with respectively labelled Aβ and native insulin as substrates for *h*IDE: Naphthyl compounds: **1** (blue dots); other 1, 4-triazoles (grey dots); 1, 5-triazoles **16–18** (open blue circles); oxadiazoles **11–12** (green dots); carboxylate analogue of the most active hydroxamate **14**(orange dot). Phenyl compounds : **10, 19** (black dots). The structures of six representative compounds are depicted on the right.

In a first round of medicinal chemistry directly inspired by the TGS experiment, we selected (Supplementary Fig. 2) and prepared chemically 12 compounds (**1**–**5**, **8**–**10** and **16**–**19**): seven 1,4-substituted triazoles and three 1,5-substituted triazoles formed in the *in situ* click experiment were prepared chemically, together with two combinations that were not fruitful in the TGS experiment. It has recently been observed that the apparent inhibitory potency differs according to the substrate used to assess the activity of IDE^23^,^32^. We therefore tested these compounds in competition with both a labelled Aβ peptide and native insulin (Supplementary Table 2). SAR obtained with compounds evidenced inhibitor **1** as the most active compound. In a second round of medicinal chemistry, several analogues of **1** were synthesized to evaluate the impact of other modifications on its structure (**6**–**7** and **11–15**). Figure 2 shows the correlation between pIC_50_s (half-maximal inhibitory concentration) values measured with Aβ peptide and native insulin as substrates, respectively. In the series of compounds, IC_50_s range from 60 nM to >100 μM. The slope of the regression line is 0.90, its intercept is 0.89 and *R*^2^=0.81 (very similar results are obtained using Ki calculated with the Cheng-Prusoff equation, as, for both substrates, the [*S*]/Km values used in our experimental settings are comparable ([*S*]/Km = 0.35 and 0.29, respectively, for Aβ16-23 and insulin)), indicating that the SAR with insulin is very similar to the one obtained with Aβ. The six compounds illustrating critical features of SAR are depicted on the left. Our results show that 1,5-triazoles are much less active than the 1,4 isomers. It is interesting to note that the templating experiment accelerates in comparable extents the formation of both 1,4 and 1,5 isomers that eventually have rather different IC_50_s. This could be explained by the flexibility of IDE in general and of its catalytic site in particular. Moreover, the triazole is not a simple linker as its replacement with an isosteric oxadiazole is detrimental to activity. The stereochemistry of the carbon in β to the hydroxamate is also important, as the enantiomer of **1** is 10-fold less active. In this series of compounds, the hydroxamate, as is usually the case with zinc metalloprotease inhibitors, cannot be replaced by a carboxylate.

### Binding mode to *h*IDE

**Figure 3:**
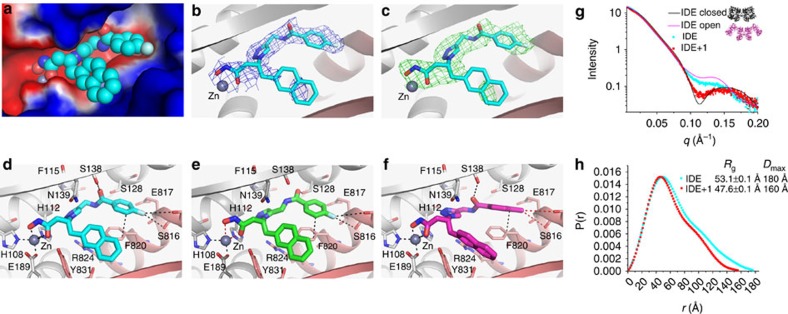
Structural and SAXS analyses of the interaction of *h*IDE with inhibitors. (**a**–**d**) Detailed interactions of *h*IDE with compound **1** (BDM44768; PDB accession code 4NXO), (**e**) **10** (BDM44619; PDB accession code 4IFH), and (**f**) **16** (BDM71290; PDB accession code 4RE9) are displayed. *h*IDE is in surface (**a**) or ribbon (**b**–**f**) representation. Compound **1**: C (cyan); Compound **10**: C (purple); Compound **16**: C (green); O (red); N (blue); F (light blue); zinc (deep grey sphere); N-terminal domain residues, (light grey); C-terminal domain residues (pink). Electrostatic potentials (**a**) that were calculated using APBS2.1, displayed using PyMol, and coloured from red (−1kT) to blue (+1kT). 2mFo-DFc map ((**b**) blue mesh), and mFo-DFc SA omit map ((**c**) green mesh) of *h*IDE bounded with compound **1** were contoured to 1*σ* and 2.5*σ*. SAXS analyses of the interaction of *h*IDE in the presence and absence of compound **1**, depicted by their scattering profile (**g**: light scattering intensity as a function of *q*=(2*π*sin*θ*/*λ* in Å^−1^) and pair distribution function of scaterers in the real space derived from the scattering profile (**h**, including calculated radius of gyration *R*_g_ and the maximum linear dimension *D*_max_). The theoretical scattering profiles of closed (black) and open (magenta) states of *h*IDE dimer are shown in **g**. SAXS data fitting was performed with CRYSOL and *P*(r) distribution function was calcultated with GNOM.

To substantiate our understanding of SAR, we used X-ray crystallography to determine the structure of compound **1**-bound *h*IDE and examine the binding mode of compound **1** (Supplementary Table 3; Supplementary Fig. 3). *h*IDE consists of two 50 kDa homologous N- and C-terminal domains (IDE-N and IDE-C). Compound **1** forms close contact with the catalytic site formed by both IDE-N and IDE-C of IDE (Fig. 3a–d). The hydroxamate tightly chelates the zinc ion at IDE-N. The fluorinated group of BDM44768 also forms a π-bond with Phe820 at IDE-C. In addition, the triazole interacts with the positively charged guanidinium of Arg824 at IDE-C. This is consistent with the high affinity of triazole for cations^33^. Interestingly, the naphthyl side chain has some conformational freedom within a hydrophobic pocket formed in the C-terminal domain of the catalytic site. The relative freedom of the naphthyl chain in the binding pocket is consistent with limited loss of potency observed when inversing the chiral centre of **1** and explains the discontinuous electron density (Fig. 3b,c). The hydrophobic contacts of the naphthyl group with F820 and F834 residues are also consistent with the observed reduction in affinity when it is replaced by the phenyl group (Fig. 2). Given this binding mode, the large differences in the value and direction of the dipole moment between triazole and oxadiazole (3.55 and 1.2 Debye, respectively)^34^ explain why isosteric oxadiazoles are less potent inhibitors. As it binds to the catalytic site formed by IDE-N and IDE-C, compound **1** appears to inhibit IDE by keeping the enzyme in a closed, inhibited conformation.

Our structures provide the molecular basis of why compound **1** binds IDE tighter than compound **10** and **16** (Fig. 3; Supplementary Fig. 2). The F-atom in compound **1** forms key hydrogen bonds with S816 and E817 but the methyl group in compound **10** does not (Fig. 3d,e). The 1, 4-triazole moiety allows compound **1** to complement well with the catalytic pocket of IDE, whereas 1,5-triazole makes compound **16** more compact (distance from the branching β carbon to F of compound **16** is ∼1 Å less than that of compound **1**) (9.2 Å versus 10.1 Å). Compound **16** thus needs to be twisted so that only suboptimal contacts are made (Fig. 3f). The dislocation of the hydroxamate moiety from zinc ion (∼0.5 Å farther from zinc ion in **16** than that in compound **1**) explains the reduced affinity.

### Solution-phase interactions

In order to confirm the physical interaction of **1** with IDE and compare it with **16**, we recorded ^19^F NMR (nuclear magnetic resonance) spectra in the absence and presence of the enzyme at several compound:enzyme ratios. Already at 10-fold excess of compound, the ^19^F NMR spectra of both compounds broadened appreciably in the presence of the enzyme, confirming the direct interaction with the enzyme. Broadening of the NMR signal of **1** was more severe, with vanishing signal when the ratio was lowered to 2:1 or 1:1. This suggests a stronger interaction for the 1,4 isomer (**1**) when compared with the 1,5 isomer (**16**), for which signal intensity remains detectable even at the 2:1 or 1:1 compound:enzyme ratio (Supplementary Fig. 4). These data are consistent with both the relative inhibitory potencies of **1** and **16** in the enzymatic assays, the observation of their formations in the click experiment, and the binding revealed by X-ray diffraction.

Our structure predicts that compound **1** could induce a conformational shift from the open state of *h*IDE into the closed one. To test this, we performed a small-angle X-ray scattering study (Fig. 3g,h). As expected, our SAXS data showed that IDE in solution preferred to be in the open state at the physiologically relevant pH and buffer conditions^35^. The addition of compound **1** profoundly reduced the values of radius of gyration (*R*_g_) (from 53.1 to 47.6 Å) and *D*_max_ (from 180 to 160 Å; Fig. 3g,h). Also, the addition of compound **1** rendered the SAXS profile of IDE to exhibit a depression at the q values ranging between 0.1 and 0.15 (Fig. 3g,h). Such depression is predicted in the theoretical SAXS profile of IDE dimer in its closed conformation, where an enclosed 13,000 Å^3^ catalytic chamber is formed, but not in the open conformation^35^. This suggests that compound **1** induced a conformational change of IDE into a smaller size. When fitting the SAXS profile with the IDE closed structure and with the open model of IDE that is derived from *Escherichia coli* pitrilysin, an IDE homologue (PDB=1Q2L), we found that ∼63% of IDE was in an open state in the absence of compound **1,** whereas only ∼16% of IDE remained in the open conformation when compound **1** was added (Supplementary Fig. 5). Thus, our SAXS data support the notion that compound **1** induces a shift from the open to the closed state of IDE in solution. We also proved that inhibition of IDE by compound **1** is reversible by showing that the enzyme activity is fully recovered after a rapid 100-fold dilution of an enzyme–inhibitor mixture made with compound **1** at IC_50_ × 10. IDE has two substrate binding sites, one at the catalytic cleft and the other at the exosite that is distal away from the catalytic cleft. Compound **1** is predicted to compete with the substrate binding at the catalytic site directly. Compound **1** would also prevent the substrate from binding to the exosite by inducing the conformational switch of IDE to the closed state. As we experimentally observe complete enzymatic inhibition at the highest concentrations of compound **1**, it is likely that the direct competition between compound **1** and substrate at the catalytic site plays the dominant role in inhibition.

### Drug-like properties of 1

To be pharmacologically useful, the inhibitor needs to be selective, soluble, stable and cell permeable. Table 1 shows that **1** is selective for IDE over a panel of metalloproteases. It also displays satisfactory physical–chemical properties (Table 1), including a very good aqueous solubility and an adequate stability in plasma from three mammalian species. The stability of the hydroxamate function in this compound is consistent with previously published structure–stability studies on this chemical function^36^. Furthermore, a bidirectional transport study performed on a polarized monolayer of Caco-2 cells *in vitro* at pH=7.4 shows that **1** displays a good cell membrane permeability at two different concentrations (Table 1).

Finally, to evaluate the suitability of compound **1** for *in vivo* pharmacological studies, we measured plasma concentrations over time in mice treated intraperitoneally with **1**. Compound **1** reaches a plasma *C*_max_ of 9.2 μM 10 min after intraperitoneal dosing (30 mg kg^−1^). It displays a significant area under the curve (AUC; 0→4 h) of 256 min μg ml^−1^ and a half-life of 80 min (Table 1).

### Effect of 1 on IDE in cell culture

IDE degrades Aβ (ref. 37) as well as the amyloid precursor protein intracellular domain^38^. Secretion of endogenous Aβ_1–40_ is significantly increased in primary neuronal cultures from *Ide*−/− mice^20^. We measured the effect of **1** and **10** on Aβ_1–40_ secreted by cultured human SY5Y neuroblastoma cells. Our enzyme-linked immunosorbent assay (ELISA) data revealed significantly increased concentrations of Aβ_1–40_ in culture supernatants of cells treated with **1**, but not with **10,** a 100-fold less active analogue (Supplementary Fig. 5). This confirms the inhibitory effect of **1** on IDE activity in a cellular context where both substrate and enzyme are endogeneously produced.

With respect to insulin, isolated mouse islets were incubated with compound **1** and the amount of insulin in the culture medium in response to increasing concentrations of glucose was measured. Compound **1** increased, in a dose-dependent manner, the quantity of insulin detectable in the supernatant of islets isolated from wild-type C57BL/6J mice, but not from *Ide*−/− mice (Supplementary Fig. 7). However, comparing islets incubated with **1** versus vehicle, the ratio of stimulation of insulin secretion by low and high glucose concentrations was not altered by the inhibitor, suggesting that **1** does not affect the response of beta cells to glucose stimulation. Therefore, the observed increase in the concentration of insulin in supernatants was very likely due to inhibition of insulin degradation within cells or in the supernatant by **1**.

### *In vivo* effect of 1 on exogeneous insulin

**Figure 4:**
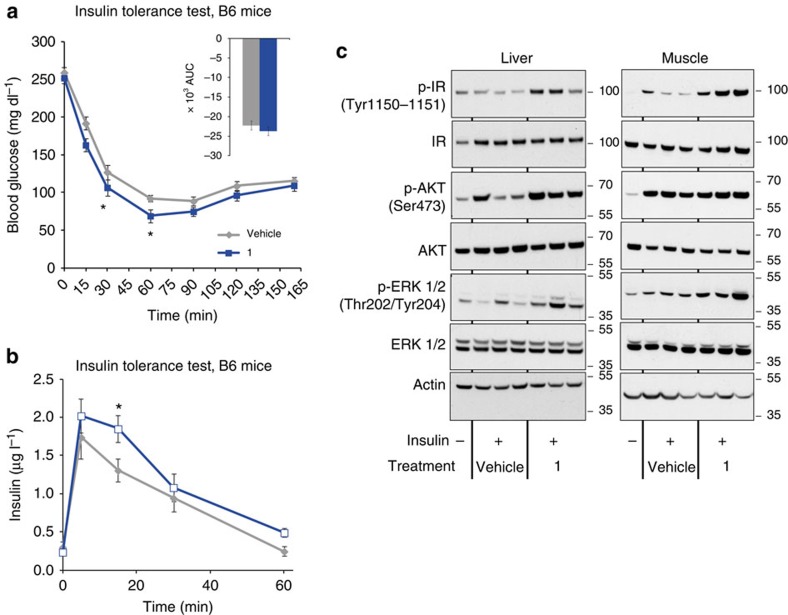
Effect of 1 on C57BL/6J mice during insulin tolerance test. C57BL/6J mice were treated with **1** at 50 mg kg^−1^ (blue lines) or with vehicle (grey lines) intraperitoneally and insulin tolerance test (ITT) was conducted by i.p. injection with 0.75 U kg^−1^ insulin on 5 h-fasted mice 15 min after intraperitoneal treatment. Blood glucose (**a**) and insulin (**b**) concentrations (**1**: blue bars; vehicle: grey bars) were measured at the indicated time points. Insert represents AUC _(0–165)_. Data are mean±s.e.m (*n*=8 mice per group), two-sided *t-*test *P<0.05; **P<0.01 ; ***P<0.001. Liver and muscle (**c**) protein expression and insulin-stimulated phosphorylation of insulin receptor, Akt and ERK evaluated by western blot analysis. Actin was used as loading control. Liver or muscle were collected 15 or 5 min after insulin injection at 0.75 U kg^−1^ or 10 U kg^−1^, respectively.

To find out whether the *ex vivo* stabilization of insulin translates in an *in vivo* setting, an insulin tolerance test (ITT) was performed in mice treated with **1** or vehicle (Fig. 4a). In this experiment, treatment with **1** slightly increased serum insulin concentrations as expected, with a significant effect at 30 min (Fig. 4b). This was associated with enhanced insulin signalling in skeletal muscle and liver. Indeed, treatment with **1** increased the phosphorylation of liver and muscle insulin receptor, as well as the downstream signalling molecules Akt and ERK, which are functional relays between insulin receptor activation and glucose uptake and cell growth and differentiation respectively (Fig. 4c). Treatment with **1** also tended to enhance the glucose lowering effect of insulin at 30 and 60 min (Fig. 4a), an effect consistent with the increased intracellular signalling.

### Effect of **1** on glucose tolerance

**Figure 5:**
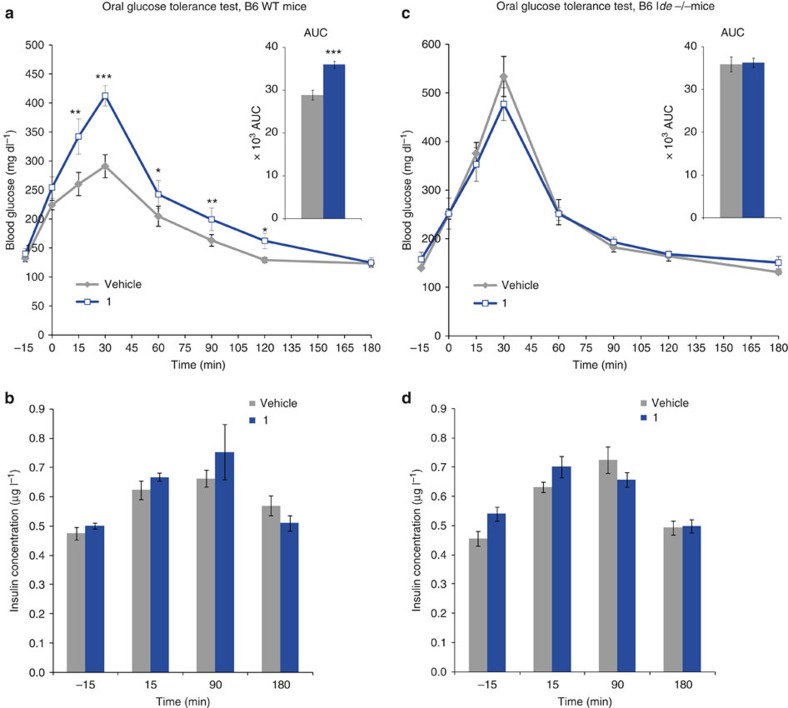
Acute *in vivo* effect of 1 in mice in an OGTT. (**a**,**c**) Mice (WT and *Ide*−/− C57BL/6J) were treated with **1** at 50 mg kg^−1^ (blue line) or with vehicle (grey line) intraperitoneally. Plasma glucose concentrations measured immediately before inhibitor injection, corresponding to *t*=−15 min, immediately before oral glucose challenge (3 g kg^−1^, at *t*=0), and up to 180 min after it. Inserts represent AUC _(−15 to 180)_. (**b**,**d**) : Insulin concentration measured in the same animals (WT and *Ide−/−* C57BL/6J) (**1**: blue bars; vehicle: grey bars) was measured at the indicated time points. One out of three independent experiments is shown. The experiment shown was performed as ‘cross-over’, such that the group initially treated with vehicle was treated two days later with **1** and vice versa. Data are means±s.e.m (*n*=6 mice per group). two-sided *t-*test *P< 0.05; **P<0.01 ; ***P<0.001.

Farris *et al.*^20^ showed that mice with homozygous deletion of the *Ide* gene (*Ide*−/−) are viable and fertile, but develop hyperinsulinemia and glucose intolerance. Subsequently *Ide*−/− mice were shown to present glucose intolerance of growing severity with age^21^. This was thought to result from the chronic exposure to high levels of insulin. To evaluate the effect of pharmacological IDE inhibition on glucose homeostasis, we treated C57BL/6J mice with compound **1** at 50 mg kg^−1^ intraperitoneally before challenging them with 3 g kg^−1^ glucose in an oral glucose tolerance test (OGTT) 15 min later. Surprisingly, treatment with **1** resulted in acute glucose intolerance as evidenced by increased plasma glucose concentrations from 15 to 90 min after oral glucose load. The AUC of glycaemia as a function of time was significantly increased (Fig. 5a). Plasma insulin levels, determined at baseline, 15 and 90 min following glucose load were similar in mice treated by **1** versus control vehicle, suggesting that the impaired glucose tolerance was not attributable to a change in insulin levels (Fig. 5b). *Ide*−/− mice did not develop glucose intolerance upon treatment with **1**, indicating that the glucose intolerance observed in wild-type (WT) mice was a direct effect of IDE inhibition by **1** (Fig. 5c,d). Interestingly, the level of glucose intolerance of Ide−/− mice was similar to the one of parallel bred WT mice of the same genetic background treated with **1** (Fig. 5a,c), suggesting that the effect of short-term IDE inhibition in WT mice is similar to that of life-long IDE deficiency. As expected, treatment with compound **1** also had no effect on plasma insulin concentrations in *Ide−/−* mice. The same OGTT performed with inactive analogue **14**, further confirms the fact that effects measured with **1** are the consequence of IDE inhibition (Supplementary Fig. 8).

**Figure 6:**
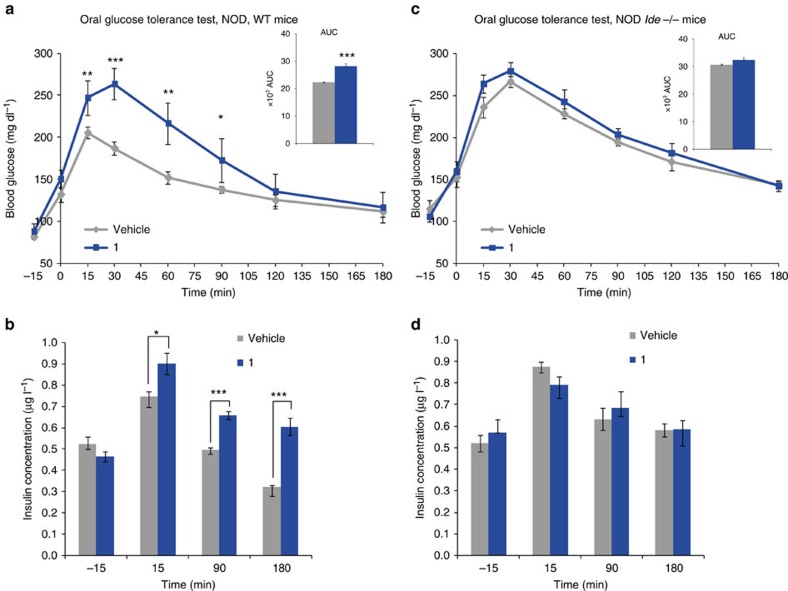
Acute *in vivo* effect of 1 on NOD mice in an OGTT. (**a**,**c**) Mice (WT and *Ide−/−* NOD) were treated with **1** at 50 mg kg^−1^ (blue line) or with vehicle (grey line) intraperitoneally, and plasma glucose concentrations measured before (−15 min) and after the oral glucose challenge (3 g kg^−1^, at *t*=0), inserts represent AUC _(−15 to 180)_. (**b**,**d**) Insulinaemia measured in the same animals as in (**a**,**c**) NOD and *Ide−/−* respectively (**1**: blue bars; vehicle: grey bars) measured at the indicated time points. One out of three independent experiments is shown. Data are mean±s.e.m (*n*=6 mice per group). two-sided *t-*test *P<0.05; **P<0.01 ; ***P<0.001.

Wishing to confirm our *in vivo* findings in animals of a second genetic background, we assessed the effect of treatment with compound **1** in female non obese diabetic (NOD) mice. As these mice spontaneously develop autoimmune insulin-dependent diabetes starting after the age of about 14 weeks in our colony, we tested 14 week-old non-insulinopenic non-diabetic NOD mice. Similar to mice on the C57BL/6J background, WT but not *Ide*−/− NOD mice, exhibited a strong impairment of glucose tolerance when treated with **1** (Fig. 6a,c). However additionally and in contrast with the C57BL/6J background, IDE deficiency in NOD mice resulted in elevated insulin levels in OGTT at 15, 90 and 180 min compared with WT (Fig. 6b,d). Note that the hyperinsulinemia induced in WT NOD mice by compound **1** was similar to the hyperinsulinemia in *Ide−/−* NOD mice treated with vehicle or **1**. Thus, like in mice of the C57BL/6J background, the effect of short-term IDE inhibition is similar to that of life-long IDE deficiency in NOD mice (compare blue bars in Fig. 6b with grey bars in 6d).

The deteriorating effect of **1** on glucose tolerance was clearly unexpected considering the effects observed sofar with the compound, which included inhibition of insulin and Aβ_1–40_ degradation *in vitro* and *ex vivo* as well as increased plasma insulin concentration and intracellular insulin signalling in the insulin tolerance test.

**Figure 7:**
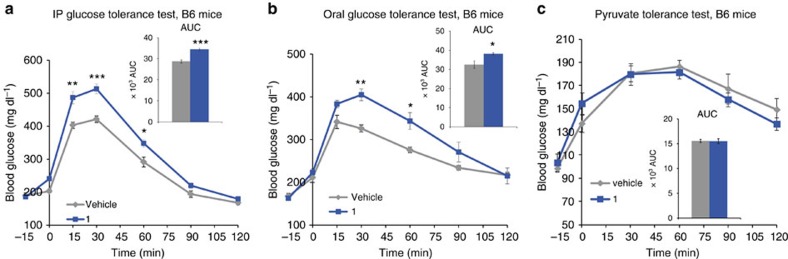
Effect of 1 on blood glucose during oral and intraperitoneal glucose administration, and pyruvate challenge. C57BL/6J mice were treated with **1** at 50 mg kg^−1^ (blue lines) or with vehicle (grey lines) intraperitoneally, and plasma glucose concentrations measured before (−15 min) and after (**a**) intraperitoneal (IP) glucose (1.5 g kg^−1^, at *t*=0), (**b**) oral glucose (2 g kg^−1^, at *t*=0), (**c**) pyruvate (2 g kg, at *t*=0). Inserts represent AUC _(−15 to 120)_. (**1**: blue bars; vehicle: grey bars) measured at the indicated time points. Data are mean±s.e.m (*n*=6 mice per group). two-sided *t-*test *P<0.05; **P<0.01; ***P<0.001.

To investigate whether the impaired glucose tolerance induced by **1** is the consequence of increased intestinal glucose absorption, we compared the effect of **1** in an OGTT with that in an intraperitoneal glucose tolerance (IPGTT) test (Fig. 7). Treatment with **1** again significantly increased plasma glucose concentrations at all time points following oral glucose administration (Fig. 7a). A similar increase was observed when glucose was administered intraperitoneally (Fig. 7b), suggesting that the impaired glucose tolerance in the OGTT upon treatment with **1** is not due to an effect of incretin production or increased glucose absorption in the intestine. Moreover, hepatic gluconeogenesis was not altered by **1** as measured during a pyruvate tolerance testing. Indeed, glucose excursion curves and AUC were similar in animals treated with **1** or vehicle (Fig. 7c).

## Discussion

We have demonstrated that kinetic TGS and medicinal chemistry enabled us to rapidly identify several inhibitors of IDE. We established SARs in enzymatic assays using insulin and Aβ peptide as substrates which were consistent with the binding mode observed in the crystal structure of IDE in complex with the most potent compound and two analogues. The strong preference of IDE in the TGS for the naphthyl group compared to the phenyl group is reflected in the SARs and in the distinct effects of **1** and **10** in cells. Interestingly, TGS formed the two possible triazole isomers that were shown later to display different inhibitory potencies and binding ability in enzymatic assays, crystallography and ^19^F NMR experiments. Owing to its selectivity and pharmacokinetic profile, the most potent compound **1** from this series was selected as a suitable pharmacological probe for whole animal studies.

Initially we studied the effect of **1** on degradation of several preferred substrates in *in vitro* and *ex vivo* assays. In an assay measuring the catalytic activity of IDE on endogenously produced Aβ in differentiated SY5Y cells, treatment with **1** led to a dose-dependent increase of Aβ_1–40_ abundance. In this model, analogue **10** which has a 100-fold lower activity against purified IDE had no effect, strongly suggesting that the effect of **1** is IDE-dependent. **1** also protected insulin secreted by murine pancreatic islets *ex vivo* from degradation, without affecting secretion. This effect was dependent on IDE inhibition as islets from *Ide*−/− animals did not respond to the treatment. Extending these findings to the *in vivo* setting, evidence that **1** can inhibit degradation of preferred substrates was obtained in an insulin tolerance test. Here treatment with **1** led to increased plasma insulin concentrations upon injection of exogenous insulin. **1** increased also intracellular signalling of exogenous insulin, and slightly enhanced its glucose lowering effect. All of these results are consistent with the recognized role of IDE as major protease acting in the degradation of insulin and Aβ.

In striking contrast to these anticipated results, treatment with **1** surprisingly increased glycaemia in mice of two genetic backgrounds when challenged with an oral or intraperitoneal glucose load. The absence of an effect of **1** in *Ide*−/− mice proved that this effect is IDE-dependent. Interestingly, there was no difference in insulinaemia and glucose excursion between *Ide*−/− mice treated with the vehicle and WT mice treated with **1**. This suggests that the pharmacological blockade of IDE by **1** in WT mice completely abolishes the function of IDE. In experiments with C57BL/6J mice, the insulinaemia in knockout (KO) and wild type (WT) mice was similar and not affected by a treatment with **1**. In contrast with the C57BL/6J background, both genetic deficiency for, and pharmacological inhibition of IDE on the NOD background resulted in a significantly increased insulinaemia during OGTT. Although the reason for this different response between the strains is unclear, it has been suggested that the late pre-diabetic phase of type-1 diabetes, which characterizes 14-week-old NOD mice used in our tests, may be associated with insulin resistance, a scenario that could explain the phenomenon. Alternatively, β-cells of NOD mice may display altered sensitivity to the regulatory input of IDE in glucose homeostasis, potentially as a consequence of β-cell stress resulting from the ongoing autoimmune attack. The observation that the insulin secretion index is lower in NOD versus C57BL/6J mice is in line with this hypothesis. However, whatever the reason for the distinct insulin response in the two strains, the most significant and unexpected result of OGTT and IPGTT studies in the mice clearly was the glucose intolerance upon treatment with **1**.

The glucose intolerance reported earlier in the IDE KO mice has been interpreted as a consequence of chronically increased insulin levels, leading Abdul-Hay *et al* to postulate that a short-acting inhibitor of IDE would exert similar effects as sulfonylureas^22^. Our results clearly do not support this interpretation as well as the prediction deduced from it and indicate that the mechanism underlying the insulin resistance phenotype in older KO mice must be more complex.

Key results obtained with **1** are not fully consistent with those published recently by Maianti *et al.*^24^ who studied the effect of acute pharmacological inhibition of IDE with a distinct novel inhibitor called 6bK. These authors observed contrasting effects on glucose tolerance: 6bK only improves glucose tolerance under conditions that mimic the intake of a meal. According to the authors, this complex pharmacology is explained by variations in the relative concentrations of circulating substrates of IDE—mainly glucagon and insulin—depending on the feeding state: 6bK would be a better stabilizer of insulin when the ratio of insulin over glucagon is high, that is, after oral feeding, owing to the incretin effect.

With **1**, we observe a spectrum of coherent biological effects all in line with the inhibition of proteolytic IDE activity and comparable to the reported effects of 6bK, with the exception of an impaired glucose tolerance observed with **1** after both intraperitoneal and oral glucose challenge. Thus, at the difference with 6bK, the effect of **1** on glucose tolerance is not affected by the incretin effect. Neither did we observe an effect of IDE inhibition by **1** on hepatic glucose production, as measured by the pyruvate tolerance test.

It is noteworthy that the binding modes of the two inhibitors to IDE are very different: 6bk binds to an exosite 11 Å away from the catalytic Zn ion, while compound **1** binds to the catalytic site, chelates the Zn ion and interacts with both N-terminal and C-terminal domains of IDE, locking it in a closed conformation. The closed conformation is observed in the co-crystal structure with **1** and its existence in solution is strongly suggested by the SAXS experiment. We could speculate that the conformational consequence of compound **1** binding to IDE may alter non proteolytic functions of IDE, including binding to other proteins with potential regulatory functions^12^,^14^, and contribute to the glucose intolerance triggered by **1** in both settings (IPGTT and OGTT). Therefore, the effects of compound **1** on other IDE-regulated proteins deserve attention in future studies as they could underpin new mechanisms regulating glucose metabolism and explain distinct effects of inhibitors with different binding sites.

Also linked to its binding mode, the differential ability of 6bK to inhibit the degradation of insulin and glucagon could explain why it does not behave like **1** in controlling glucose tolerance in mice. On the basis of structures of IDE in complex with insulin^39^ and glucagon^9^, the site occupied by 6bK is crucial for the binding of insulin but not for glucagon. However, 6bk binds near the anchoring site that recognizes the N terminus of substrates, where glucagon needs to bind for its degradation. Thus, it is reasonable to speculate that 6bK may behave as a full inhibitor for insulin degradation by IDE while only as a partial inhibitor for glucagon. This could explain why 6bK only improves the glucose intolerance in the oral glucose tolerance test.

In conclusion, we have provided conclusive evidence that compound **1** (BDM44768) can be used for temporary manipulation of IDE catalytic function *in vitro* and *in vivo*. We speculate that this may be of particular interest in animal models of chronic or degenerative diseases such as type-2 diabetes and Alzheimer’s disease. Most importantly, our results lead us to conclude that, contrary to various suggestions in the literature, acute inhibition of the catalytic function of IDE is not a generally applicable option to treat type-2 diabetes.

## Methods

### Orthogonal partition of alkyne mixtures

The 90 alkynes were sorted in two orthogonal partitions (X and Y) which were used in two parallel TGS experiments. Partition X contains nine clusters (X_1_ to X_9_) of 10 alkynes each, while partition Y contains 10 clusters (Y_1_ to Y_10_) of nine alkynes each (Supplementary Fig. 3). Because these partitions are orthogonal to each other, a given cluster of partition X has only one alkyne in common with any cluster of partition Y. As a consequence of this arrangement, each alkyne is used in two different competing environments in the two parallel TGS experiments. This was meant to maximize the chance of forming and detecting binding triazoles amongst the possible combinations.

### Mixtures of alkynes

Mixtures of alkynes (Supplementary Table 1) were prepared from a dimethylsulfoxide (DMSO) 51 mM stock solution of each alkyne: mixtures X (9 clusters of 10 alkynes): mixing 100 μl of stock solutions of 10 alkynes to reach 5.1 mM final concentration of each alkyne; mixtures Y (10 clusters of 9 alkynes): mixing 100 μl of stock solutions of nine alkynes mixed and 100 μl of the DMSO to reach 5.1 mM final concentration of each alkyne. Each azide was dissolved in DMSO at 10.2 mM final concentration.

### Synthetic 1, 4-triazoles controls

In each well were mixed 3 μl of an azide at concentration of 25.5 mM in DMSO, 3 μl of an alkyne mixture (5.1 mM), 2 μl of a water solution of CuSO_4_ (20 mM) and 296 μl of a mixture of *t*-BuOH/H_2_O (1/1). The plate was shaken at room temperature during 36 h.

### General protocol for kinetic target-guided synthesis

In a 96-well plate, were mixed 2 μl of azide (10.2 mM), 2 μl of a given mixture of alkyne (each alkyne at 10.2 mM) and 200 μl of the enzyme IDE-E111Q-CF (4.8 μM) in a buffer solution (Hepes, pH=7.4). The 96-well plate was sealed and shaken at 37 °C for 72 h. Samples of the reactions (50 μl) were diluted in MeOH (50 μl) and injected (20 μl) for liquid chromatography–mass spectrometry (LC–MS)-time of flight analysis. Hits were identified in each cluster by mass and retention time and compared to both incubation with buffer in place of the enzyme and synthetically prepared triazoles obtained in mixtures.

### Synthesis of inhibitors

Compound **1** (BDM44768) and analogues were prepared using synthetic procedures described in the Supplementary Methods.

### *In vitro h*IDE inhibition of Aβ_16–23_-based substrate hydrolysis

*In vitro* IDE activity was measured with a quenched substrate ATTO_655_-Cys-Lys-Leu-Val-Phe-Phe-Ala-Glu-Asp-Trp. Human recombinant IDE was cloned and purified as previously described. Briefly, human IDE (1.87 ng μl^−1^) was incubated 10 min at 37 °C with compound in Hepes 50 mM, NaCl 100 mM, pH 7.4 and the enzymatic reaction was started by adding the substrate (final concentration 5 μM). After 30 min, samples (1% DMSO final) were excited at 635 nm and fluorescence emission at 750 nm was measured. All measurements were carried out as 8-point dose response curves and reported as the average of at least three independent measurements. EDTA was used as a reference inhibitor (100% inhibition at 2 mM). Data analysis was performed using Xlfit v 5.0 and GraphPad Prism v 4.0. Nonlinear curve fitting and statistical analysis was done using built-in functions.

### *In vitro h*IDE of insulin hydrolysis

The enzymatic activity of *h*IDE was assayed quantifying the amount of insulin (Actrapid) at the end of the reaction. The quantity of insulin was determined using a Luminex kit (HMHMAG-34K, Millipore). 20 μl of WT IDE in Hepes buffer (50 mM with 100 mM NaCl, pH 7.4) at 1 μg ml^−1^ were pre-incubated 10 min at ambient temperature with 20 μl of test compound or vehicle in 96-well microtiter plates (dark, non-binding surface). The reaction was then started by the addition of 40 μl of insulin at 40 nM. The final concentration of *h*IDE and substrate was 0.25 μg ml^−1^ and 20 nM, respectively. Incubations were performed at ambient temperature for 10 min. The reaction was then stopped by addition of 80 μl of 200 mM EDTA. The samples were diluted by a factor of 3, and 15 μl were transferred to 96-well microtiter plates (dark, clear bottom, non-binding surface) containing 15 μl of assay buffer (from Luminex Kit). Fifteen microlitre of insulin magnetic beads were added. Incubation was performed during 16–18 h at 4 °C under agitation. Wells were washed three times and 30 μl of hormone panel antibodies were added. The samples were incubated 30 min at ambient temperature under agitation. 30 μl of strepdavidin-phycoerythrin were added. Incubation was performed 30 min at ambient temperature under agitation. Wells were washed three times and 80 μl of drive fluid were added. The plate was then read with the MagPix apparatus. Data analysis was performed using Xlfit v 5.0 and GraphPad Prism v 4.0. Nonlinear curve fitting and statistical analysis was done using equation 1:




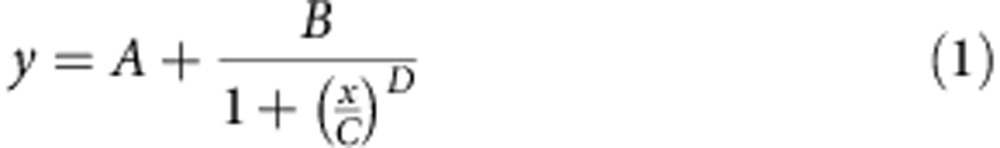




where *A* is minimum value of *y*; *B* is range of *y*; *C* is the IC_50_ value and *D* is slope factor.

### General Absorption, Distribution, Metabolism and Excretion (ADME) methods

For LC−MS/MS, a Varian HPLC−MS/MS system 1200L triple−quadrupole mass spectrometer equipped with an electrospray ionization source was used. Analytes were separated in incubation mixtures by HPLC with a Luna C18, 5 μm, 50 mm × 2.1 mm column (Phenomenex) or a Acquity BEH C18, 50 × 2.1 mm, 1, 7 μm column (Waters). The mobile phase solvents used were 0.1% formic acid in water (A) or 0.1% formic acid in acetonitrile (B) using a gradient: 2–98% B for 2.30 min; hold at 98% B for 1.00 min; 98–2% B for 0.10 min; 2% B hold for 1.50 min. The injection volume was 10 μl, and the flow rate was 0.6 ml min^−1^. MS analysis were performed with MRM detection using the parameters optimized for each compounds, with MS Workstation software (version 6.3.0 or higher).

### Solubility/Log*D* measurements

Ten microlitre of a 10 mM solution in DMSO of the compound are diluted either in 490 μl of PBS pH 7.4 or in organic solvent MeOH in a 700 μl-microtube (in triplicate). The tubes are gently shaken 24 h at room temperature, then centrifuged for 5 min at 4,000 r.p.m. The mixtures are filtered over 0.45 μm filters (Millex-LH Millipore). Twenty microlitre of sample are diluted in 180 μl of MeOH. The solubility is determined by the ratio of mass signal area PBS/ organic solvent. Forty microlitre of a 10 mM solution in DMSO of the compound were diluted in 1.960 ml of a 1/1 octanol /PBS at pH 7.4 mixture. The mixture was gently shaken 2 h at room temperature. Twenty microlitre of each phase was diluted in 480 μl of MeOH and analysed by LC–MS. Each compound is tested in triplicate. Log *D* was determined as the logarithm of the ratio of concentration of product in octanol and PBS respectively, determined by mass signals.

### Microsomal stability

Male mouse (CD-1) liver microsomes (BD Gentest) were used. All incubations were performed in duplicate in a shaking water bath at 37 °C. The incubation mixtures contained 1 μM compound with 1% methanol used as a vehicle, mouse liver microsomes (0.6 mg of microsomal protein per mL), 5 mM MgCl_2_, 1 mM NADP, 5 mM glucose 6-phosphate, 0.4 U ml^−1^ glucose 6-phosphate dehydrogenase, and 50 mM potassium phosphate buffer (pH 7.4) in a final volume of 1.5 ml. Samples were taken at 5, 10, 20, 30 and 40 min after microsome addition, and reactions stopped by adding ice-cold acetonitrile containing 1 μM internal standard (four volumes). Centrifugation of the samples for 10 min at 10,000*g* and 4 °C allowed to pellet precipitated microsomal proteins, and supernatant was subjected LC−MS/MS analysis, in acetonitrile containing 1 μM internal standard. Each compound was quantified by converting the corresponding analyte/internal standard peak area ratios to percentage drug remaining, using the initial ratio values in control incubations as 100%. Propranolol, known as a high hepatic clearance drug in rodents, was used as a quality-control compound for the microsomal incubations. *In vitro* intrinsic clearance (CLint expressed as μl min^−1^ mg^−1^) was calculated according to: the following formula : CLint=dose/AUC∞, where dose is the initial amount of drug in the incubation mixture (1 μM) and AUC∞ is the area under the concentration versus time curve extrapolated to infinity. The slope of the linear regression from log percentage remaining versus incubation time relationships (-*k*) was used in the conversion to *in vitro t*_1/2_ values by: *t*_1/2_=−0.693/*k*.

### Caco-2 permeation assay

Caco-2 cells were provided by ATCC and checked for mycoplasma contamination. These cells are not listed by ICLAC as misidentified cell lines (October 3rd, 2014). A total of 0.4 × 10^5^ Caco-2 cells (ATCC no. HTB-37), at passage 28, were seeded on 25 cm^2^ plastic flask and changed every second days with complete medium containing high glucose DMEM with L-glutamine supplemented by 10% of fetal calf/bovine serum, 1% of non-essential amino acids without L-glutamine. The paracellular barrier characteristics of Caco-2 cells monolayer was monitored using (i) measurement of transendothelial epithelial resistance and (ii) measurement of the permeability to the non-permeant fluorescent molecule, Lucifer Yellow. Caco-2 cells were trypsinized after 3 days of incubation while they cover 80–90% of the flask and seeded at a density of 5 × 10^5^ in 75 cm^2^ flasks in complete medium supplemented with 73 nM (∼0.04 μg ml^−1^) of the antibacterial puromycin (3′-[α-amino-*p*-methoxyhydrocinnamamido]-3′-deoxy-N,Ndimethyladenosine dihydrochloride). After 5–6 days, Caco-2 cells reach high cells density (>0.5 × 10^5^ cells per cm^2^) and are then passage into cell HTS 24-well plates with 0.4 μm polycarbonate membrane inserts. Cells were seeded at 600,000 cells per cm^2^ (200,000 cells per insert) and cultivated for 6 days in complete medium with puromycin. Media was replaced every second days. Compound solutions were prepared in HEPES-buffered Ringerŕs (RH) solution (NaCl 150 mM, KCl 5.2 mM, CaCl_2_ 2.2 mM, MgCl_2_ 0.2 mM, NaHCO_3_ 6 mM, Glucose 2.8 mM, HEPES 5 mM, water for injection), pH=7.4 at a final concentration of 1 or 10 μM for tested drugs. For A→B transport experiment, 0.2 ml of the compound solution was placed on the apical side of the cells and samples were taken from the basolateral compartment. For B→A transport experiment 0.8 ml of the solution was placed on the basolateral side of the cells and samples were taken from apical side. Transport Studies were done in Transwell polycarbonate: HTS 24-well plate inserts (surface area: 0.33 cm^2^–0.4 μm pore size). Cells were equilibrated for 10 min in transport buffer prior to the transport experiment, and then incubations with compounds were performed at 37 °C under agitation. After 1 h aliquots were taken from each compartment and sampled in 96-well plates with glass insert. Permeation and the % of recovery are calculated using the formulas below:




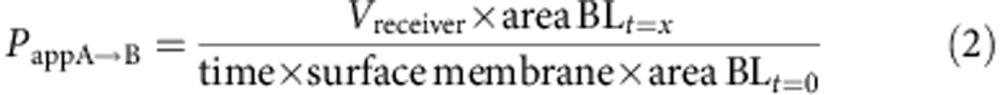







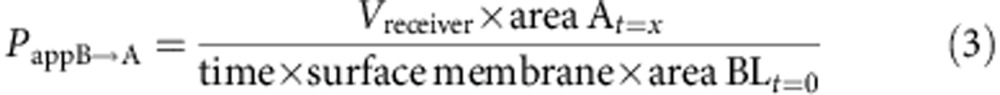







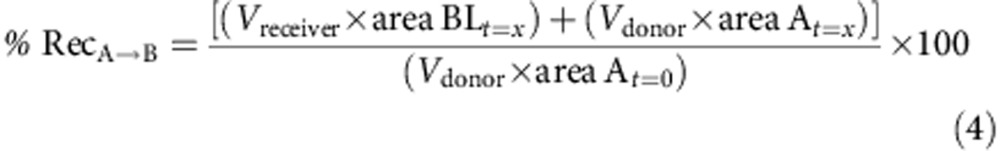







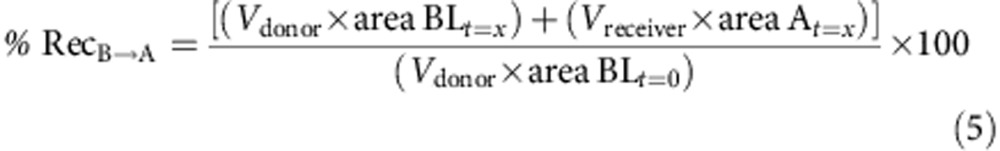




### Stability in plasma or in cell culture media

Incubations were performed in duplicate in Eppendorf tubes. The plasma (mouse plasma, rat plasma (mixed gender), human (mixed gender, all lithium heparine, from Sera Laboratories International) or cell culture medium (differenciation medium: DMEM (Life Technologies, Ref 41965), 0.4 % gentamicine, 10 % FCS (Lonza, Ref DE14-801F) or proliferation medium: DMEM (Life Technologies, Ref 41965), 0.4 % gentamicine, 2 % horse serum (Gbco, Ref16050) was pre-incubated 5 min at 37 °C before the addition of test compounds to a final concentration of 10 μM (1% DMSO maximum). At the defined time points, 50 μl from each tube were removed to another tube containing 450 μl of cold CH_3_CN+ internal standard (1 μM). After centrifugation (10 min at 10,000 r.p.m.), supernatants are analysed. The degradation half-life (*t*_1/2_) values were calculated as *t*_1/2_=0.693/*k* where *k* is the first-order degradation rate constant. The degradation rate constant (*k*) was estimated by one-phase exponential decay nonlinear regression analysis of the degradation time course data using Xlfit software (version 2.1.2 or higher) from IDBS.Ltd.

### Selectivity

1. *In vitro* Neutral endopetidase (NEP) activity was measured with a quenched substrate *N*-Dansyl-D-Ala-Gly-p-nitro-Phe-Gly (*K*_m_=350 μM). Briefly, human NEP (R&D Systems; 200 ng ml^−1^) was incubated 10 min at room temperature with compound in Hepes 50 mM, NaCl 100 mM, pH 7.4 and the enzymatic reaction started by adding the substrate (final concentration 200 μM). After 2 h, samples (1% DMSO final) are excited at 340 nm and fluorescence emission at 535 nm is measured on a Victor3 V1420 Perkin Elmer spectrophotometer. All measurements are reported as the average of at least three independent measurements (DL-thiorphan reference inhibitor (IC_50_=1(±0.3) nM)).
2. *In vitro* ACE activity was measured with a quenched substrate Abz-Gly-p-nitro-Phe-Pro-OH (*K*_m_=180 μM). Briefly, human ACE (R&D Systems) was incubated 10 min at room temperature with compound in Tris 50 mM 1% NaCl pH 7.4 and the enzymatic reaction is started by adding the substrate (final concentration 300 μM). After 17 h, samples (1% DMSO final) are excited at 340 nm and fluorescence emission at 420 nm is measured on a Victor3 V1420 Perkin Elmer spectrophotometer. All measurements are reported as the average of at least three independent measurements (Captopril reference inhibitor (IC_50_=11 nM)).
3. Inhibition of endothelin-converting enzyme (hECE), matrix metalloprotease 1 (hMMP1). *In vitro* activity was assayed at CEREP.SA as follows. Briefly, recombinant human ECE-1 (NSO cells) was incubated 45 min at room temperature with compound and enzymatic reaction was started using ECE-1 fluorescent substrate (15 μM). Recombinant human MMP-1 (*E. coli*) activity was measured using DNP-Pro-Cha-Gly-Cys(Me)-His-Ala-Lys(n-Me-Abz)-NH_2_ (10 μM) after incubation with compound 40 min at 37 °C.

### Cell culture and Aβ secretion

The human neuroblastoma cell lines SH-SY5Y were provided from ATCC (cat. no. crl2266) with a Certificate of Analysis and not checked in the lab for mycoplasma contamination. These cells are not listed by ICLAC as misidentified cell lines (3/10/2014). Cells were maintained in DMEM/F12 glutaMAX medium (Life Technologies) supplemented with 10% FBS, 1% non-essential amino acids, 50 U ml^−1^ penicillin, 50 μg ml^−1^ streptomycin (Life Technologies) in a 5% CO_2_ humidified incubator at 37 °C. SH-SY5Y were seeded in a 96-well plate at 200,000 cells per cm^2^. After 24 h all-trans-retinoic acid (Sigma) was added at 10 μM and medium was renewed every 2 or 3 days for 1 week. Differentiated cells were then incubated with compounds at 10 μM and 30 μM in presence of 0.1% DMSO. After 48 h Aβ 1–40 concentration was determined by ELISA (Invitrogen, cat. no. KHB3482) according to the manufacturer recommendations.

### Cell culture and glucose-stimulated insulin secretion

Islet preparation: the pancreatic duct was cannulated and injected with 2 ml of collagenase D dissolved at 0.66 mg ml^−1^ in HBSS (Hanks Balanced Salt Solution), followed by removal of the pancreas and incubation at 37 °C for 18 min. Digestion was stopped by addition of cold HBSS with 10% FCS. The islets were washed twice in HBSS with FCS and once in RPMI with FCS. After resuspension in DMEM with 10% FCS, the islets were hand-picked repeatedly using a binocular, seeded at 15 islets per well of round bottom 96-well plates in RPMI containing 10% FCS, 1 mM Hepes and 11.2 mM glucose, and rested overnight. The next day, the islets were incubated in fresh DMEM with 10% FCS without glucose for 90 min. Then glucose at 2.8 mM together with BDM44378 was added; after 1 h, the supernatant was removed and replaced by fresh medium with 28 mM glucose, followed by another 1-h incubation in the presence or absence of inhibitor. Finally the insulin content in supernatants was quantified using an insulin ELISA (cat. no. 10-1247-01, Mercodia).

### Crystallization, data collection and structure determination

IDE cysteine-free (IDE-CF) and IDE wild type (IDEWT) were obtained as previously published^39^. IDE-CF was mixed with **1** (BDM44768), **10** (BDM44619) or **16** (BDM71290) at a molar ratio of 1 to 10 (protein to compound) following by the fractionation on a Superdex S-200 column to purify protein-compound complex. The extensive purification of IDE using gel-filtration chromatography was a key step to ensure the formation of diffracting quality crystals of IDE. The complex of IDE-CF with **1** was then co-crystallized by hanging drop vapour diffusion at 18 °C using 1 μl of 10 mg ml^−1^ IDE and 1 μl of mother liquor (10–13% PEG MME 5000, 100 mM HEPES pH 7.0, 4–14% Tacsimate, 10% dioxane) and the addition of 200 μM **1** in the crystallization drop to ensure the high occupancy of **1** in IDE. IDE crystals were equilibrated in a cryo-protective buffer containing the mother liquor with 30% glycerol and flash frozen in liquid nitrogen. Diffraction data were collected at 100 K at the Advance Photon Source 19-ID beamline at Argonne National Laboratory. The data sets were processed using HKL2000 (ref. 40) and the structures were solved by molecular replacement using software Phaser^41^ and the IDE-CF-E111Q portion of insulin-bound IDE-CF-E111Q (PDB:2WBY) structure as a search model. Structure refinement and rebuilding were performed using software, Coot^42^ and Phenix^43^. The extra electron density at the catalytic site in the structure of **1**-bound IDE was clearly visible based on *σ*_A_-weighted Fo–Fc map calculated by software Phenix and the compound models were manually built using software Coot. The refinement statistics are summarized in Supplementary Table 2. Figures were generated using software Pymol.

### SAXS data collection and processing

SAXS data were collected at the APS 18-ID (BioCAT) with the Mar 165 CCD detector at room temperature (24 °C). The incident X-ray wavelength is 1.033 Å. *h*IDE was purified as described before. After 2 gel-filtration, *h*IDE was concentrated and dialysis against 20 mM Hepes, pH 7.2, 100 mM NaCl. A total of 1 mg ml^−1^ of IDE was used for data collection and 20 mM compound **1** was added in 1:100 dilution immediately prior to SAXS data acquisition. The data reducing and all data processing were carried out using ATSAS. *P*(r) function was calculated with GNOM^44^, and SAXS data fitting was performed with CRYSOL^45^.

### ^19^F NMR

The ^19^F NMR measurements were done at 20 °C using 3 mm tubes containing 200 μl of sample on a Bruker 600 MHz Avance I spectrometer equipped with a CP-QCI-F cryoprobe with Fluor Cryo-detection. ^19^F NMR experiments were done on control samples with compound **1** or compound **16** at 50 μM, 37.5 μM, 25 μM, 12.5 μM and 6.25 μM, in a 50 mM Hepes 100 mM NaCl buffer with a constant percentage (1%) of DMSO. The ^19^F titration experiments were carried out on samples containing 50 μM, 37.5 μM, 25 μM, 12.5 μM and 6.25 μM of compounds (**1** and **16**) with a constant enzyme concentration of 5 μM in the same buffer. The carrier frequency was set at −100 p.p.m. with a spectral window of 40 ppm, and 4,024 scans were recorded per sample, except for the 6.25 μM sample for which 12,288 scans were recorded.

### Pharmacokinetics

Compound **1** (BDM44768) was dissolved in pure DMSO and administered at 30 mg per kg body weight by intraperitoneal route to male C57Bl6/N 8-week-old mice (∼25–30 g) (Charles River). Three mice per time point were anesthetized with isoflurane and aliquots taken from the retroorbital sinus using sampling heparinated tubes (4 °C) at 10, 20, 30 min, 1, 2 and 4 h after administration of a single dose of ligands. The blood samples were centrifuged (5,000*g*, 15 min) for plasma separation. Plasma samples were thawed on ice. Aliquots of 50 μl were precipitated with 450 μl of ice-cold acetonitrile containing (R)-N-[3-(1-Hydroxycarbamoylmethyl-2-naphthalen-2-yl-ethyl)-3H-[1,2,3]triazol-4-ylmethyl]-benzamide (0.5 μM) used as internal standard. The samples were vigorously mixed with a vortex and centrifuged at 10,000 r.p.m. at 4 °C for 10 min, and the supernatants were transferred into tubes for LC−MS−MS analysis (Supplementary Table 2). Spiked standard solutions (10, 50, 100, 500, 1,000, 5,000, 10,000 and 50,000 nM) were prepared the same way. The protocol was approved by the ethical comitee CEEA Nord Pas de Calais (CEEA75).

### OGTT and IPGTT tolerance test in WT and *Ide−/−* mice

OGTT and IPGTT were performed on 11 to 16 week-old male or female mice (*n*=4–7 per experimental group). C57BL/6J mice were purchased from Charles River Laboratories (France), Stock no. 000664. C57BL/6J *Ide−/−* mice were obtained from Guenette^20^ and bred in the animal facility of INSERM U1151. WT C57BL/6J mice of the same genetic background were bred in the same animal facility in parallel. NOD Ide−/− mice were generated by backcrossing C57BL/6J Ide−/− mice with NOD wt mice bred in the animal facility of INSERM U1151 and maintained in this facility together with WT NOD mice. Normoglycemic WT and *Ide−/−* female NOD mice aged 14 weeks were used for OGTTs. Following a 6 h fasting period, a small tail cut was made, basal blood glucose was determined. Mice were randomized according to blood glucose and received an intraperitoneal injection of vehicle (DMSO) or compound **1** (50 mpk). Fifteen minutes later, blood glucose concentrations were measured before and after (up to 120 min, as shown) oral (2 g kg^−1^ or 3 g kg^−1^, as shown) or intraperitoneal (1.5 g kg^−1^) glucose administration. In one series of OGTTs using 3 g kg^−1^ glucose and shown in Fig. 5, groups of WT and *Ide−/−* were inverted between experiments, such that mice initially treated with vehicle were treated 2 days later with **1** and vice versa. Blood glucose levels were determined using an Accu-Check glucometer (Roche Diagnostics). Areas under the curves (AUC) for OGTT and IPGTT were calculated to evaluate glucose tolerance. Plasma insulin concentrations were measured by ELISA (cat. no. 10-1247-01,Mercodia) in blood samples collected 15 min before and 15 and 90 min relative to oral glucose injection. The protocol was approved by the ethical comitee CEEA Paris Descartes (CEEA34).

### Insulin tolerance test

ITT was conducted by i.p. injection with 0.75 U kg^−1^ insulin 15 min after intraperitoneal treatment, on 5 h-fasted mice (12 week-old male C57BL/6J , *n*=8 for each group, randomized on blood glucose at the end of the fasting period). Blood samples for glucose and insulin measurements were obtained up to 160 min after injection. AUC for ITT were calculated to evaluate insulin sensitivity. Blood glucose levels were determined using an Accu-Check glucometer (Roche Diagnostics). Insulin concentrations were quantified by ELISA (cat. no. 10-1247-01, Mercodia) in blood samples. The protocol was approved by the ethical comitee CEEA Nord Pas de Calais (CEEA75).

### Pyruvate tolerance test

Pyruvate tolerance test was performed on 18 h-fasted mice (12 week-old male C57BL/6J mice, *n*=8 for each group, randomized on blood glucose at the end of the fasting period). Basal blood glucose was determined, followed by intraperitoneal injection of vehicle (DMSO) or compound **1** (50 mpk). Fifteen minutes later, blood glucose was measured before and after (up to 120 min) intraperitoneal sodium pyruvate (2 g kg^−1^) injection. The AUC of glycaemia versus time were calculated to estimate the total glucose synthesized from pyruvate. Blood glucose levels were determined using an Accu-Check glucometer (Roche Diagnostics). The protocol was approved by the ethical comitee CEEA Nord Pas de Calais (CEEA75).

### Insulin signalling by western blotting

Liver and muscle were homogenized in a lysis buffer (1% triton, 0.5 % deoxycholate, 10 mM Na pyruvate, 2 mM Na Vanadate, 100 mM NaF) supplemented with the proteases inhibiteurs cocktails) and the resulting homogenate clarified by centrifugation. Proteins expression and phosphorylation were evaluated by western blot analysis. The signals were detected with a Pierce ECL western blotting substrate (Thermo scientific), according the manufacturer instructions. Primary antibodies were diluted at 1/1,000 and incubated overnight in PBS 0.1% Tween 20, 5% milk. Anti p-InsR beta (Tyr 1150-1151, cat. no. 3024), anti-phospho Akt (Ser473, cat. no.4058), anti-phospho Erk ½ (Thr202/Tyr204, cat. no. 4370), anti-Akt (cat. no. 9272) and anti-Erk 1/2 (cat. no. 4695) were from Cell Signaling; anti-InsR (cat. no. SC-559) and anti-Actin (cat. no.SC-1616) (loading control) were from Santa-Cruz.

### Statistics for *in vivo* studies

Group size were compliant with the guidelines of the ethical commitee (6<*n*<15 in our case). Statistical significance was determined by using a two-sided *t*-test. Normality was checked using a Shapiro–Wilk test. Homogeneity of variances has been checked. *P* values<0.05 were considered as significant and different levels of significance are represented by asterisks : *P<0.05; **P<0.01; ***P<0.001. No investigator blinding was applied.

## Additional information

**Accession codes:** X-ray structures have been deposited in Protein Database under accession codes 4NXO (*h*IDE in complex with compound **1**); 4IFH (*h*IDE in complex with compound **10**), and 4RE9 (*h*IDE in complex with compound **16**).

**How to cite this article:** Deprez-Poulain, R. *et al.* Catalytic site inhibition of insulin-degrading enzyme by a small molecule induces glucose intolerance in mice. *Nat. Commun.* 6:8250 doi: 10.1038/ncomms9250 (2015).

## Supplementary Material

Supplementary Information Supplementary Figures 1-9, Supplementary Tables 1-6, Supplementary Methods and Supplementary References

## Figures and Tables

**Table 1:** Drug-like properties of **1**.

**Selectivity (IC**_**50**_ **, μM)**	**Physical properties and stability**	**Membrane permeability (*****P***_**app**_**, 10**^−6^ **cm s**^−1^)	**Pharmacokinetics**
*h*IDE: 0.06	Log*d*_7.4_: 2.2	A→B: 1 μM: 3.3	*C*_max_ (μg ml^−1^): 4.1
*h*NEP: 2.6	Solubility (μM): 58.0	10 μM: 2.4	*C*_max_ (μM): 9.2
*h*ACE: >10.00	Plasma stability *m* (*t*_1/2_, h): 24.0	B→A: 1 μM: 19	*t*_max_ (min): 10
*h*ECE: 6.50	Plasma stability *r* (*t*_1/2_, h): 13.3	10 μM: 17	*t*_1/2_ (min): 80
*h*MMP-1: 10.00	Plasma stability *h* (*t*_1/2_, h): >24.0		AUC_0→4h_ (min μg ml^−1^): 256
	Microsomal stability *m* (*t*_1/2_, min): 20		

IC_50_, half-maximal inhibitory concentration; h, human; *h*IDE, human insulin-degrading enzyme; m, mouse; hMMP-1, matrix metalloprotease 1; NEP, neutral endopeptidase; r, rat.

Selectivity of **1**: values are means of two experiments minimum, s.d.±10%; substrate for IDE: native insulin; >10 μM stands for <10% at 10 μM. Physical properties and stability of **1**; *t*_1/2_>24 h stands for 100% remaining compound at 24 h. Cell membrane permeability of **1** assessed on a Caco-2 cell monolayer; bidirectional transport was measured at 37 °C, for a time period of 1 h (pH: 7.4/7.4); Permeability classification: low=(*P*_app_ A→B)<1.0 × 10^−6^cm s^−1^; high=(*P*_app_ A→B)>1.0 × 10^−6^ cm s^−1^. Pharmacokinetic parameters for **1** (30 mg kg^−1^, *ip*) injected in male mice (*n*=3 for each time point) as a DMSO solution.
