# Supplementary material for: Catalytic site inhibition of insulin-degrading enzyme by a small molecule induces glucose intolerance in mice
Source: Nat Commun. 2015 Sep 23;6:8250. doi: 10.1038/ncomms9250 (PMC4580987; doi:10.1038/ncomms9250)
Supplement: Supplementary Information — Supplementary Figures 1-9, Supplementary Tables 1-6, Supplementary Methods and Supplementary References [file ncomms9250-s1.pdf]

a. b.

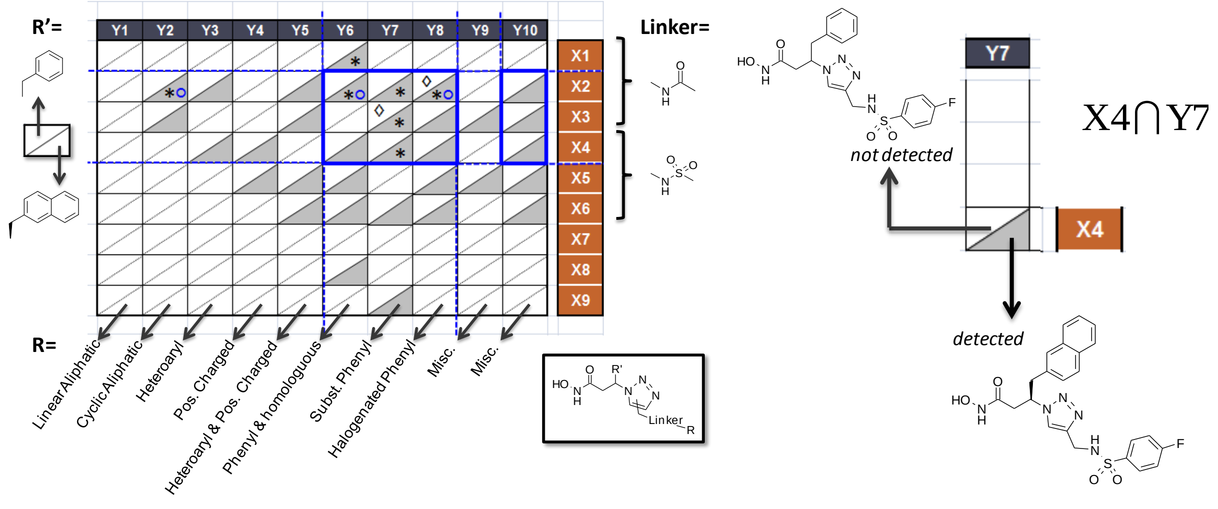

c.

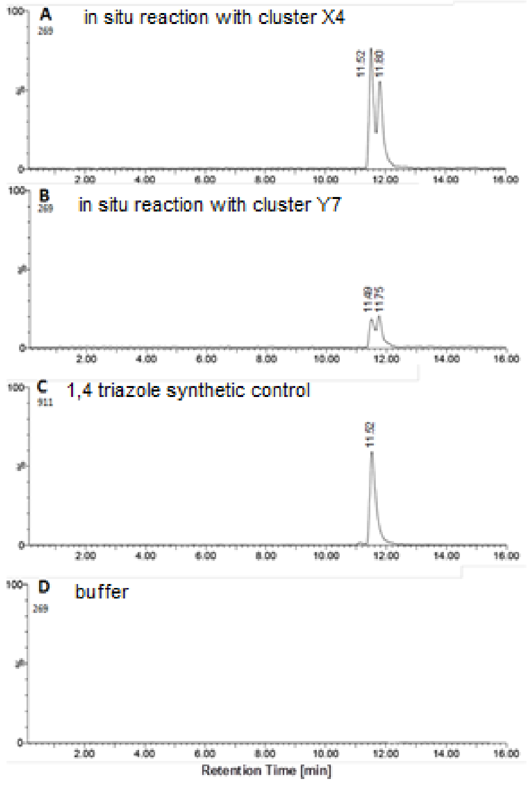

d.

	Advantages	Caveats
**Kinetic TGS**	Target selects its own ligands from a large combinatorial diversity).	Possible disconnect between rate of acceleration and Kd.
**LC-TOF-HRMS hit detection**	Confirms the molecular formula of hits.	Not quantitative.
**Single component TGS**	1 combination evaluated at a time (two possible regioisomers).	Slow and resource consuming especially at the LC-MS detection step.
**Multicomponent TGS**	Time & resource saver.	Each reagent is in competition with the others.
**Orthogonal multicomponent TGS**	Restores different competitive environments for each alkyne.	Need for cluster designs.

Supplementary Figure 1: Orthogonal multicomponent kinetic TGS experiment results and interpretation
(a) Results of multicomponent kinetic TGS displayed by orthogonal partition of alkyne mixtures, and azides (phenyl or naphthyl: upper or lower triangle respectively). Formed triazoles (gray triangles): resynthesized 1,4 triazole (stars),1,5 triazole (open circles); 1,4 Triazoles not formed but resynthesized (open diamonds); Higher hit-populated cluster intersections are highlighted with blue squares. **(b)** example of mixture deconvolution; **(c)** LC/MS-TOF analysis of kinetic TGS incubations and controls with the naphthyl-derived azide (A) Incubation sample with mixture *X4* and IDE (B) Incubation sample with mixture *Y7* and IDE (C) 1,4-triazole (anti) synthetic reference compound obtained from azide and cluster *X4* in the presence of $CuSO_4$ (D) Incubation sample of azide with cluster *X4* and buffer, ($(M+H^+$: 484.146). All detected combinations were mixtures of 1,4 and 1,5 isomers. The intensity of the triazole peak varies in both clusters for each detected combination. **(d)** Features of our experimental setup for kinetic TGS.

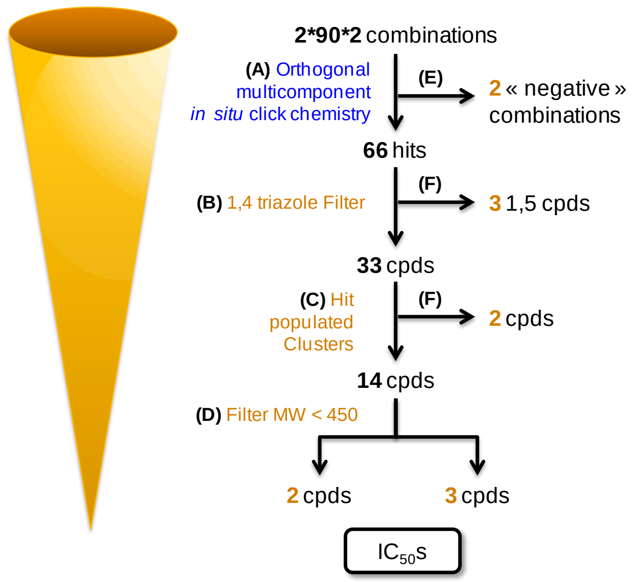

Supplementary Figure 2: Flow chart for hit resynthesis for 1st round of SAR. Flow chart for selection of 10 positive combinations and 2 negative combinations from in situ click chemistry using filters **A-E**. **(A)** Selected if detected in the orthogonal multicomponent *in situ* click chemistry (ratio Signal/Noise > 10). **(B)** Selected if 1,4-triazoles, as they are more easily synthesized than 1,5-triazoles at larger scale via conventional chemistry. **(C)** Selection if belong to higher hit-populated cluster intersections: X2-4 ∩ clusters Y6-8;10 (Supplementary Figure S1). **(D)** Selection if MW below 450. **(E)** Selection of negative combinations derived of benzyl azide to complete SARs. **(F)** Selection of compounds that did not pass filters A-D to complete early SARs (similarity filter). Flow chart was done using PipelinePilot™ from Biovia**.**

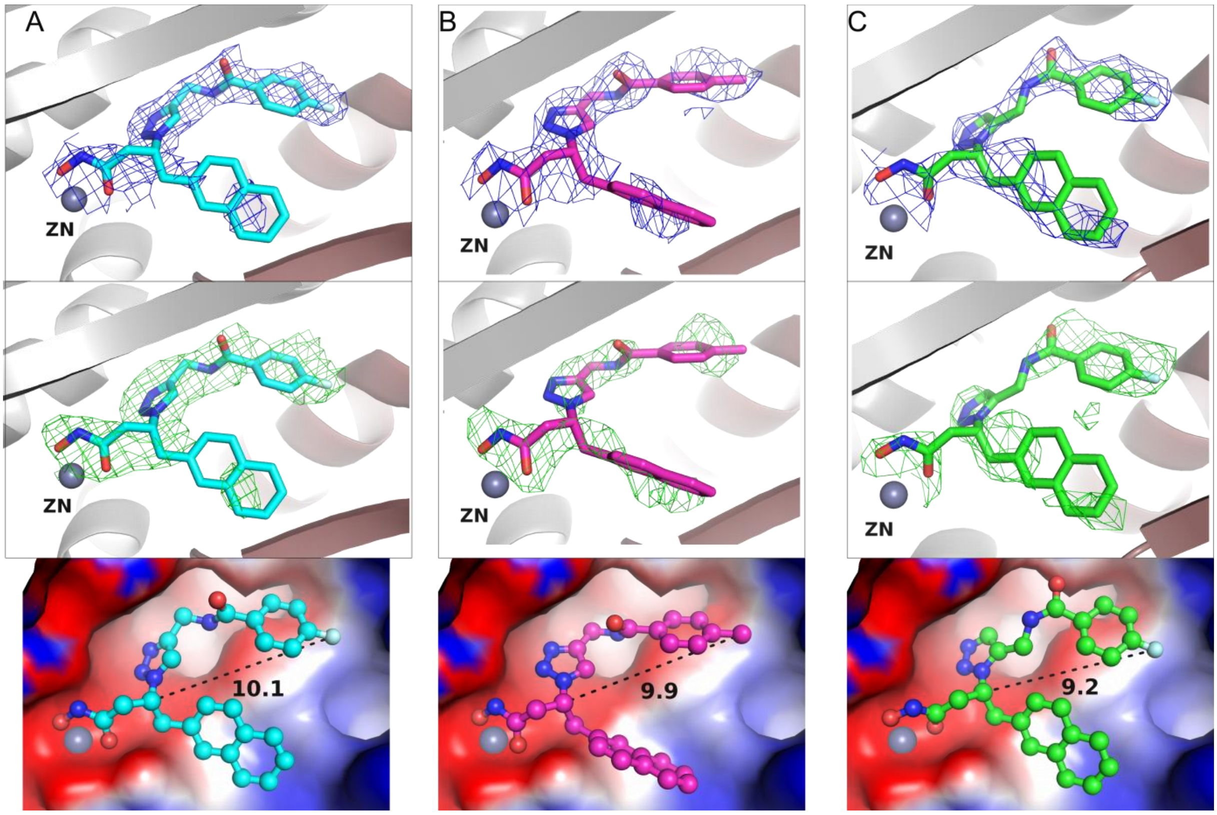

Supplementary Figure 3: 2mFo-DFc map (top, blue mesh) and mFo-DFc SA omit map (middle, green mesh) and surface electrostatic potential (bottom) of IDE-cf bounded with compound **1** (A), **10** (B), or **16** (C). 2mFo-DFc map was contoured to 1 σ, and mFo-DFc SA omit map was contoured to 2.5 σ.

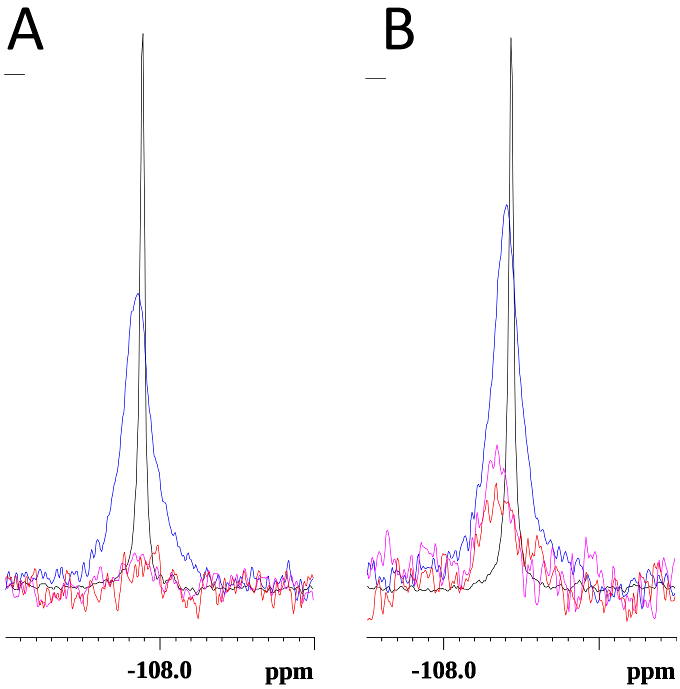

Supplementary Figure 4: ^{19}F-NMR of Compound **1** and **16** in the presence of hIDE. The ^{19}F signal of 50 µM (black) of isolated compound **1** (A) and compound **16** (B) are compared with the spectra of 50 µM (blue), 12.5 µM (magenta) and 6.25 µM (red) of the compounds in the presence of 5 µM enzyme. Spectra in the presence of the enzyme have been scaled by a factor of 4 compared to the spectra of the free compounds.

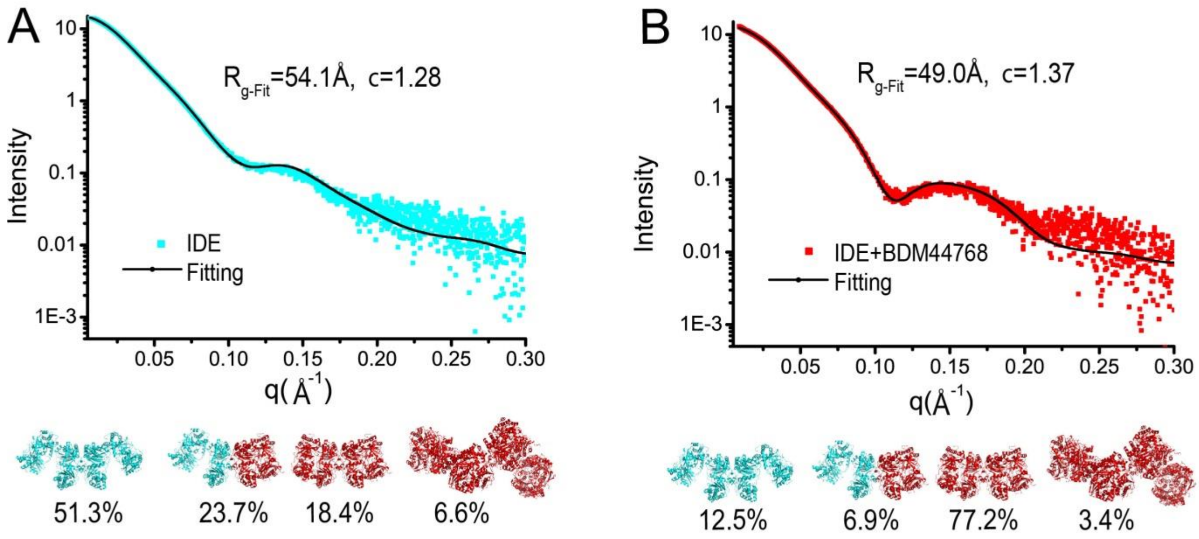

Supplementary Figure 5: SAXS analysis of IDE with compound **1**. Model fitting of SAXS data of IDE with the scattering data for *h*IDE alone (A) and *h*IDE in complex with compound **1** (B).

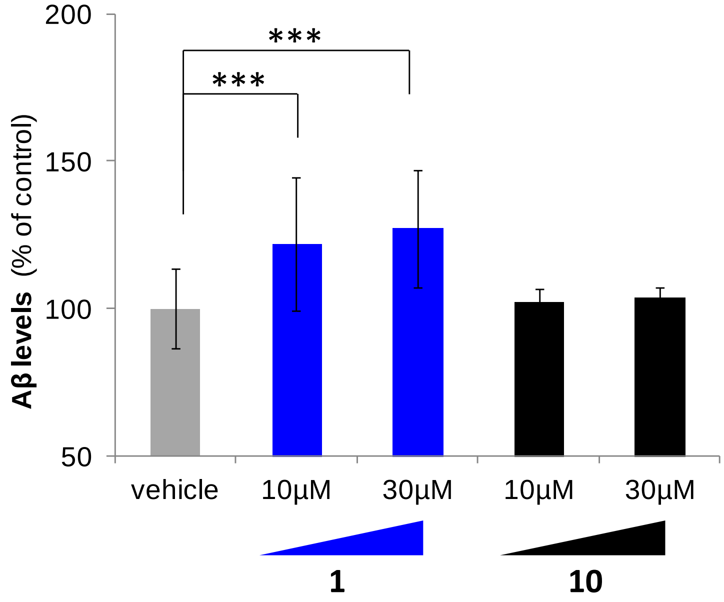

Supplementary Figure 6. Effect of **1** and inactive analogue **10** on extracellular content of $A\beta_{1-40}$ peptide in differentiated SY5Y cells. Extracellular content of $A\beta_{1-40}$ peptide for cells treated with **1** (blue bars) and its inactive analogue **10** (black bars) at two concentrations. The content is expressed as the percentage of content in the supernatant of vehicle-treated cells. Data shown are mean +/- SD (n=14). two-sided t-test *** $p < 0.001$.

IDE is a well-established protease capable of degrading Aβ[1] as well as the amyloid precursor protein intracellular domain (AICD)[2]. Endogenously secreted $A\beta_{1-40}$ is significantly elevated in primary neuronal cultures from ide KO[3]. We thus measured the effect of **1** and **10** on $A\beta_{1-40}$ accumulation in cultured human SY5Y neuroblastoma cells. Our ELISA data revealed significant elevations of the peptide in the culture treated with **1,** but not with **10,** a 100 fold less active analog. This confirms the inhibitory effect of **1** on IDE activity in cell culture.

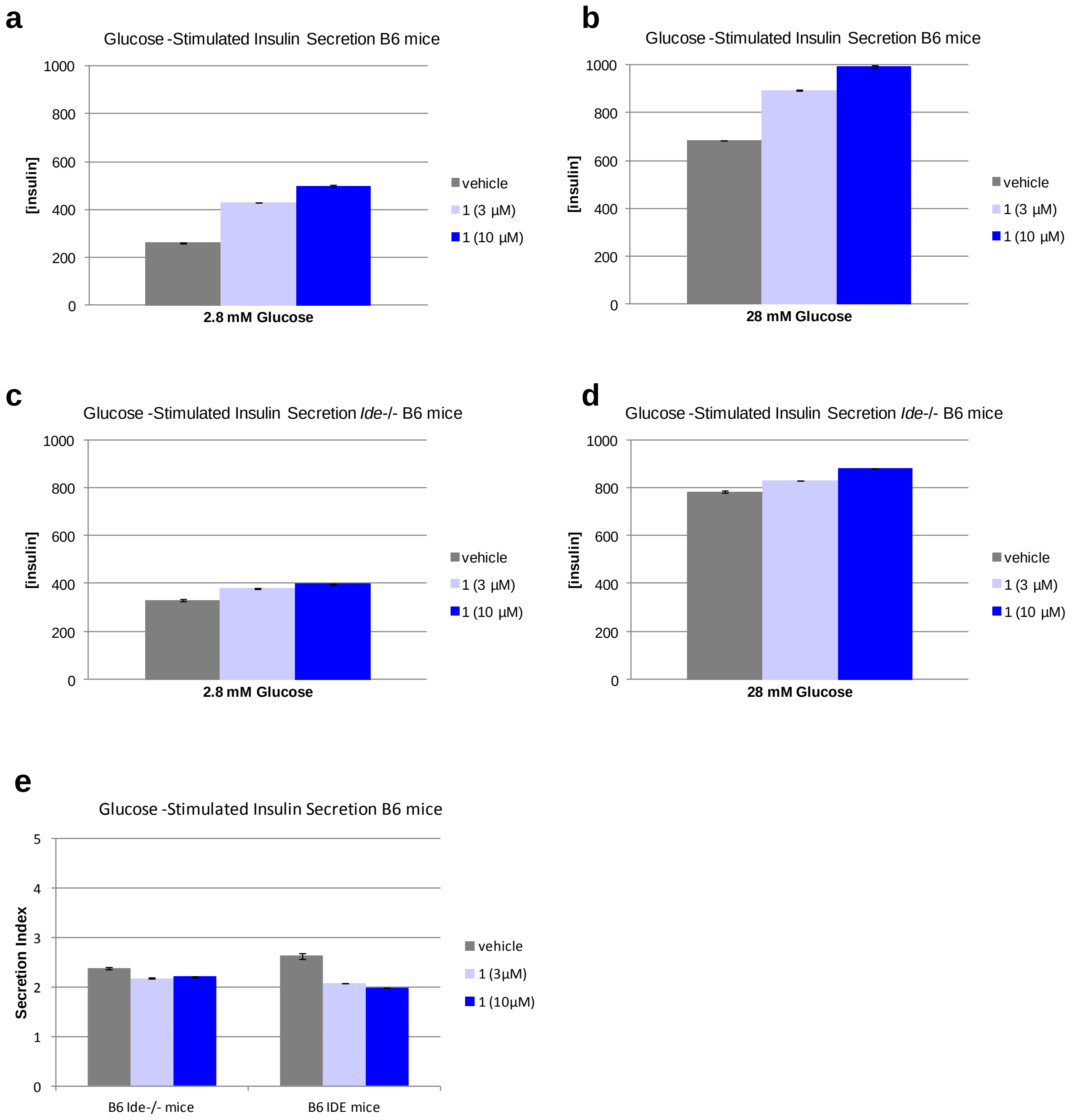

Supplementary Figure 7. Glucose-Stimulated Insulin Secretion on islets from C57BL/6J wt and Ide-/-mice. Islets isolated from C57BL/6J wild type (a, b) and Ide KO (c, d) were treated successively with 2.8 and 28 mM glucose, in the presence of compound **1** at 3 or 10µM (blue bars) or vehicle (grey bars). The concentrations of insulin (µg/L) in supernatants were then measured after 1 hour incubation time using ELISA. Data are expressed as means of two measurements. Insulin secretion indexes (e) were calculated as the ratio of concentrations of insulin measured at 28mM glucose to the concentrations measured at 2.8 mM for each treatment condition (blue bars for compound **1**, grey bars for vehicle). Data shown are mean +/- s.e.m (n=3).

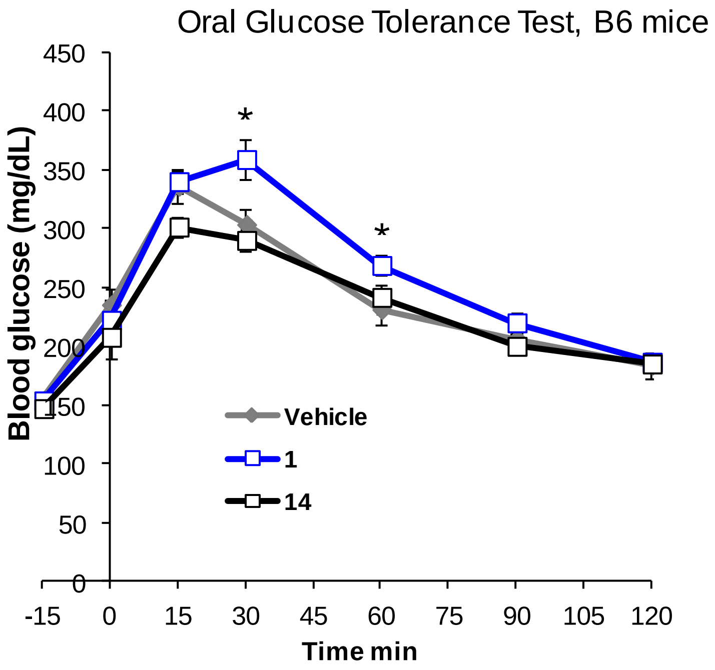

Supplementary Figure 8. Acute *in vivo* effect of **1** and inactive analogue **14** in mice during the oral glucose tolerance test. WT C57BL/6 mice were treated with **1** (blue line) or **14** (black line) at 50 mg/kg or with vehicle (grey line) intraperitoneally, and plasma glucose concentrations measured before (-15 min) and after the oral glucose challenge (2g/kg, at t=0). Data are mean ± s.e.m (n=6). two-sided t-test * $p < 0.05$.

Liver

Anticorps anti-phospho-Insulin receptor beta (Tyr1150/1151) – 95 kDa – Cell signaling

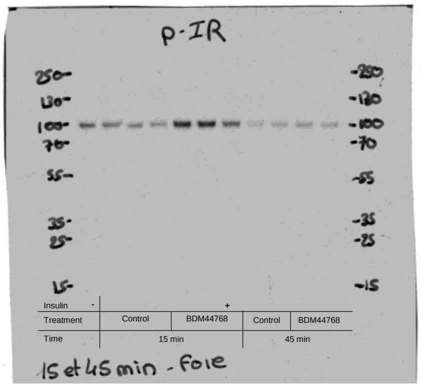

Liver

Anticorps anti-Insulin receptor beta – 95 kDa – Santa Cruz biotechnology

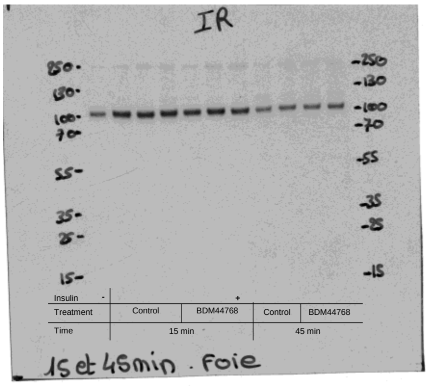

Liver

Anticorps anti-phospho-AKT (Ser473) – 60 kDa – Cell signaling

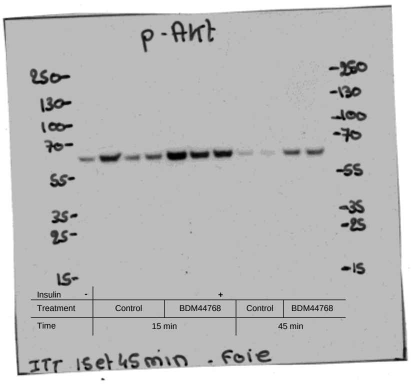

Liver

Anticorps anti-AKT – 60 kDa – Cell signaling

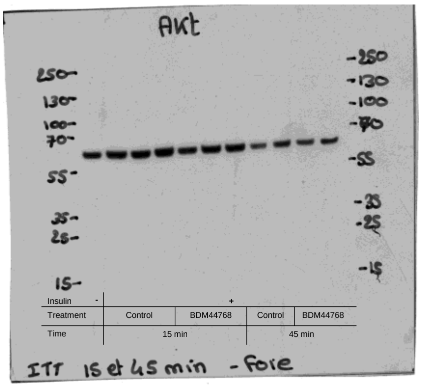

Liver

Anticorps anti-phospho-p44/42 MAPK (Erk1/2) (Thr202/Tyr204) – 44/42 kDa – Cell signaling

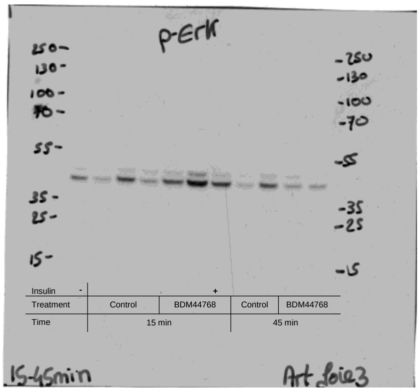

Liver

Anticorps anti-p44/42 MAPK (Erk1/2) – 44/42 kDa – Cell signaling

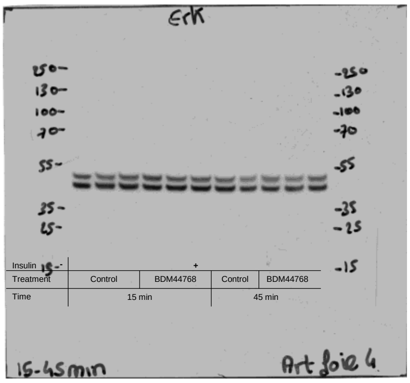

Liver

Anticorps anti-Actine – 43 kDa – Santa Cruz Biotechnology

AKT (60kDa) appears upper Actine (detection of actine after detection of AKT)

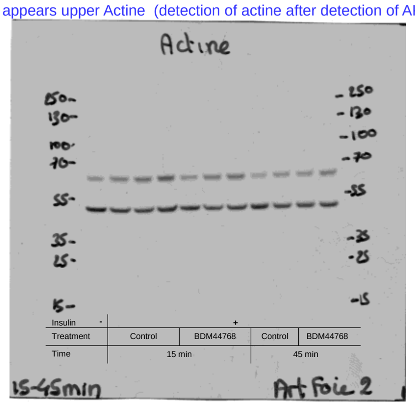

Supplementary Figure 9: Full Western blots of the ITT experiments.
Part 1 : Liver

Muscle

Anticorps anti-phospho-Insulin receptor beta (Tyr1150/1151) – 95 kDa – Cell signaling

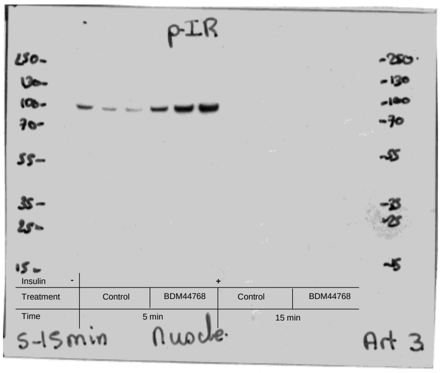

Muscle

Anticorps anti-Insulin receptor beta – 95 kDa – Santa Cruz biotechnology

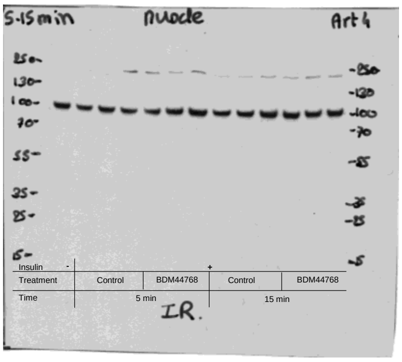

Muscle

Anticorps anti-phospho-AKT (Ser473) – 60 kDa – Cell signaling

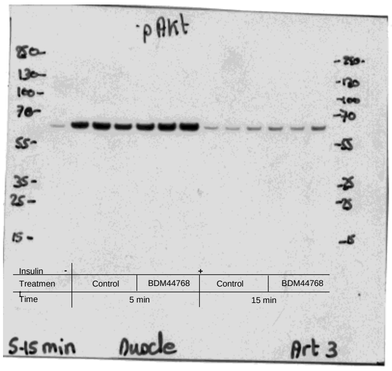

Muscle

Anticorps anti-AKT – 60 kDa – Cell signaling

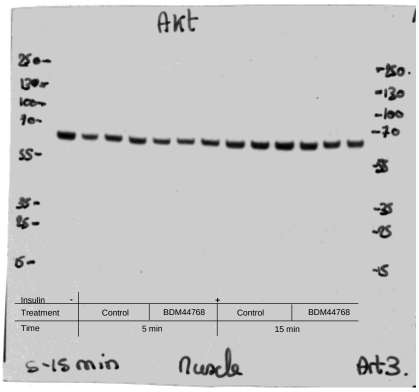

Muscle

Anticorps anti-phospho-p44/42 MAPK (Erk1/2) (Thr202/Tyr204) – 44/42 kDa – Cell signaling

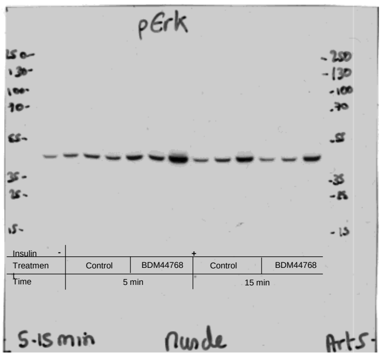

Muscle

Anticorps anti-p44/42 MAPK (Erk1/2) – 44/42 kDa – Cell signaling

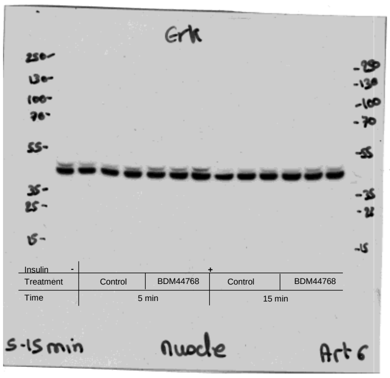

Muscle

Anticorps anti-Actine – 43 kDa – Santa Cruz Biotechnology

p-AKT (60kDa) appears upper Actine (detection of actine after detection of p-AKT)

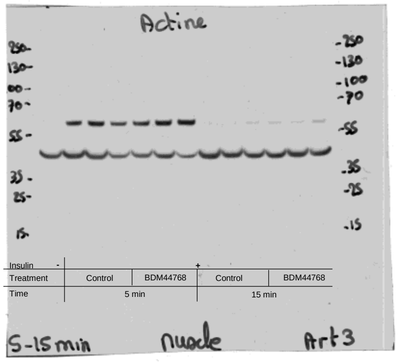

Supplementary Figure 9: Full Western blots of the ITT experiments.
Part 2: Muscle

Supplementary Table 1: Partitions of Mixtures Xn and Ym of alkynes [a,b,c]

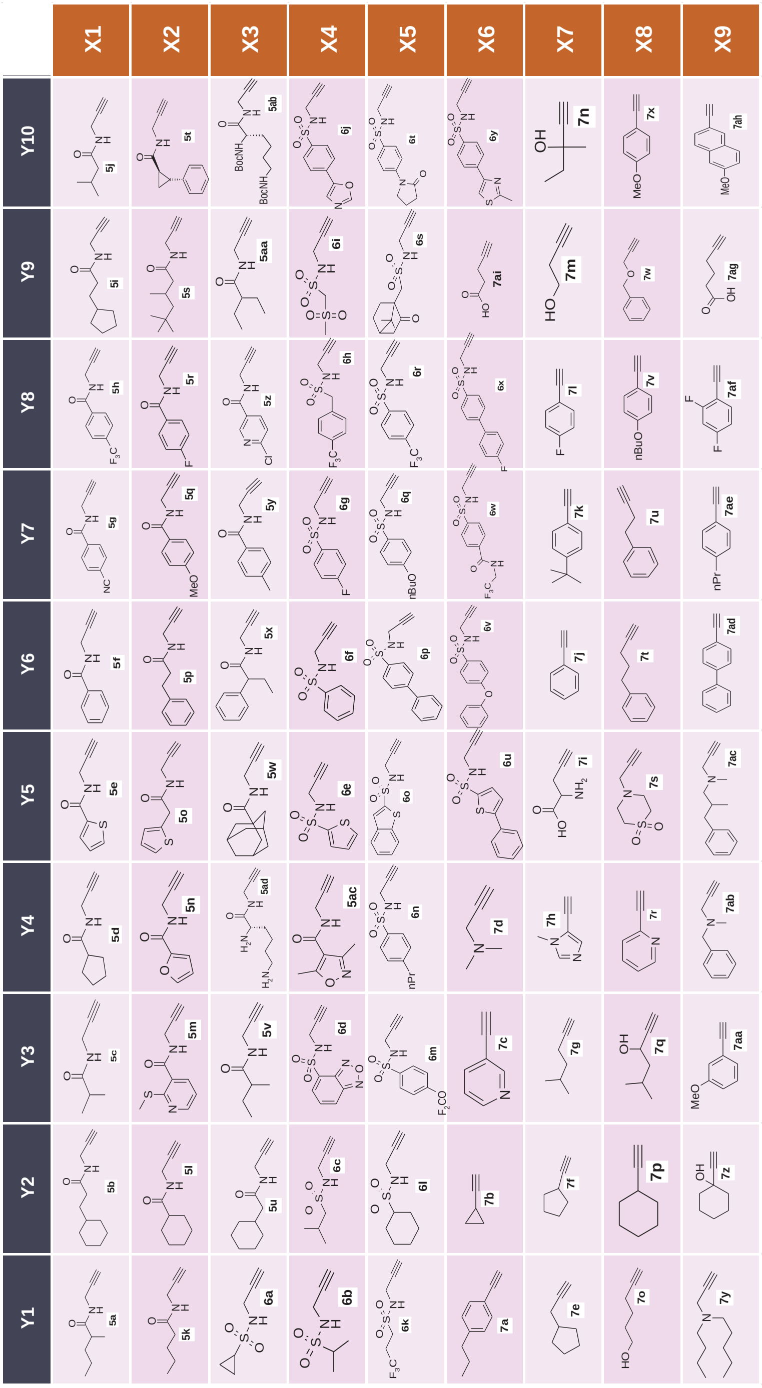

	X1	X2	X3	X4	X5	X6	X7	X8	X9
Y10	5j	5t	5ab	6j	6t	6y	7n	7x	7ah
Y9	5i	5s	5aa	6i	6s	7ai	7m	7w	7ag
Y8	5h	5r	5z	6h	6r	6x	7l	7v	7af
Y7	5g	5q	5y	6g	6q	6w	7k	7u	7ae
Y6	5f	5p	5x	6f	6p	6v	7j	7t	7ad
Y5	5e	5o	5w	6e	6o	6u	7i	7s	7ac
Y4	5d	5n	5ad	5ac	6n	7d	7h	7r	7ab
Y3	5c	5m	5v	6d	6m	7c	7g	7q	7aa
Y2	5b	5l	5u	6c	6l	7b	7f	7p	7z
Y1	5a	5k	6a	6b	6k	7a	7e	7o	7y

[a] The diversity of the acyl or sulfonyl alkynes and of the global set of alkynes was evaluated using PipelinePilot from Biovia™. [b] 90 alkynes were used, out of which 30 amides and 25 sulfonamides synthesized from propargylamine and acyl- or sulfonyl-chlorides respectively (**5a-ad**, **6a-y**, Scheme S1 and Supplementary methods) and 35 commercial alkynes **7a-7ai**. [c] The 90 alkynes were sorted in 2 orthogonal partitions (X and Y). Clusters X result from sorting the alkynes by the type of linker, while clusters Y result from sorting the alkynes by the type of R-group. No isobar compounds were within the same cluster. Partition X contains 9 clusters (X1 to X9) of 10 alkynes each, while partition Y contains 10 clusters (Y1 to Y10) of 9 alkynes each. A given cluster of partition X shares only one alkyne in common with any cluster of partition Y. Each alkyne is used in two different competing environments in the TGS experiment. All this is made to maximize the chance of forming and detecting all the binding triazoles amongst the possible combinations.

Supplementary Table 2. *h*IDE inhibition of triazoles **1-19**.

1st round of SAR: *in situ* click chemistry		
compd	IC_{50} (µM)[a] $A\beta_{16-23}$ hydrolysis	IC_{50} (µM)[b] insulin hydrolysis
**1**	0.30 (0.20-0.32)	0.06 (0.03-0.35)
**2**	0.70 (0.63-1.0)	0.51 (0.25-1.0)
**3**	2.7 (1.9-3.9)	6.7 (3.2-16)
**4**	4.5 (3.2-6.3)	3.4 (1.9-6.3)
**5**	0.80 (0.63-1.0)	0.44 (0.25-0.79)
**8**	4.4 (3.2-6.3)	1.0 (0.40-2.5)
**9**	5.7 (3.9-7.9)	6.0 (0.83-7.8)
**10**	>100 [c]	6.1 (3.2-10)
**16**	>100 [c]	12.1 (6.3-25)
**17**	>100 [c]	30.0 (19-63)
**18**	>100 [c]	>100 [c]
**19**	80 (72-99)	>100 [d]

2nd round of SAR:		
compd	IC_{50} (µM)[a] $A\beta_{16-23}$ hydrolysis	IC_{50} (µM)[a] insulin hydrolysis
**6**	3.2 (2.5-3.9)	0.74 (0.39-1.2)
**7**	12.0 (7.9-20)	2.3 (1.2-3.9)
**11**	74 (63-79)	9.6 (5.0-16)
**12**	29.0 (25-32)	6.5 (3.9-12)
**13**	67 (63-78)	50.0 (33-59)
**14**	>100 [b]	30.0 (16-41)
**15**	2.8 (1.9-4.0)	2.0 (1.2-3.2)

[a] IC_{50} (µM) (CI 95%), labelled $A\beta_{16-23}$
[b] IC_{50} (µM) (CI 95%), full insulin.
[c] < 10% inhibition at 100 µM.
[d] 50% inhibition at 300 µM.
IC_{50} are the mean of 3 to 10 experiments.

Supplementary Table 3: Data collection and structure refinement statistics of IDE in complex with **1, 10,** and **16.**

	**IDE-CF-1 BDM44768**	**IDE-CF-10 BDM44619**	**IDE-CF-16 BDM71290**
**Data Collection**			
Beamline	APS-19ID	APS 19ID	APS-19ID
Wavelength (Å)	0.9793	0.9793	0.9793
Space group	$P6_5$	$P6_5$	$P6_5$
Cell dimension(Å)			
a	262.9	263.0	263.0
b	262.9	263.0	263.0
c	86.3	90.6	90.5
Resolution (Å)	50-2.75	50-3.3	50-2.90
Rsym (%)[a]	18.8	17.9	29.2
I/sigma	15.2 (1.8)[e]	6.8 (2.2)[e]	13.5 (2.2)[e]
Redundancy[b]	4.8 (3.3)[e]	2.9 (2.6)[e]	5.4 (3.8)[e]
Completeness (%)	100.0 (100.0)[e]	98.9 (99.6)[e]	99.8 (100.0)[e]
Unique reflections	90121	51394	78626
**Refinement**			
R_{work} [c]	0.1697	0.184	0.1592
R_{free} [d]	0.2188	0.224	0.2129
No. atoms			
Protein	15560	15515	15549
Water	155	114	98
B-factors			
IDE	65.0	32.1	45.5
Compound	85.2	83.0	83.0
Water	61.7	28.3	38.6
r.m.s. deviations			
Bond lengths (Å)	0.004	0.005	0.006
Bond angles (°)	0.761	0.886	0.896
Ramachandran plot (%)			
Favorable region	97.43	97.5	97.0
Allowed region	2.57	2.5	3.0
Generously allowed region	0.0	0.0	0.0
Disallowed region	0.0	0.0	0.0
PDE code	4NXO	4IFH	4RE9

[a] $R_{merge} = \Sigma (I - \langle I \rangle)/ \Sigma \langle I \rangle$
[b] N_{obs}/N_{unique}
[c] $R_{work} = \Sigma_{hkl} ||F_{obs}| - k\,|F_{calc}||/ \Sigma_{hkl} |F_{obs}|$
[d] $\boldsymbol{R}_{free}$, calculated the same as for $\mathbf{R}_{work}$ but on the 5% data excluded from the refinement calculation.
[e] the outer resolution shell. Values in parentheses indicate the highest resolution shell

Supplementary Table 4. Optimum Mass Spectrometer Conditions and Fragmentations of Compound **1**.

parent ion, *m/z*	predominant product ion, *m/z*	capillary (V)	CE (V)	dwell time (ms)	drying gas temp. (°C)
446.1 ($[M-H]^-$)	218.9	70	15	0.1	400

Supplementary Table 5: Comparison of compound **1** with **6bK**.

6bk (Maianti *et al*, Nature 2014)	1, this paper
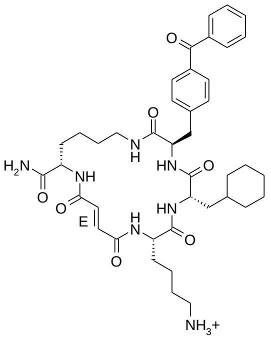	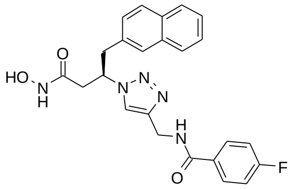
Systemic distribution after ip administration	Systemic distribution after ip administration
Specific, reversible inhibitor of IDE	Specific, reversible inhibitor of IDE
Binds to an exosite distinct from catalytic site	Binds to the catalytic site
IC_{50} approx 10^{-7}M	IC_{50} approx10^{-7}M
NT	Changes the conformation of IDE in solution (SAXS)
NT	Inhibits degradation of insulin secreted by mouse islets *ex vivo*, (controlled vs KO)
Augments insulin action in vivo (ITT)	Augments insulin action *in vivo* (ITT)
NT	Increase insulin signaling in liver and skeletal muscle
Impairs glucose tolerance in IPGTT (IDE dependant)	Impairs glucose tolerance in IPGTT (IDE dependant)
Improves glucose tolerance in OGTT (IDE dependant), GLP-1 dependant, insulin driven	Impairs glucose tolerance in OGTT (IDE dependant)
Lowers gluconeogenesis (in a pyruvate tolerance test)	No effect on gluconeogenesis (in a pyruvate tolerance test)

NT: Not Tested

Synthetic Chemistry

Supplementary Table 6: Structures of compounds tested and key intermediates.

**test compounds**				
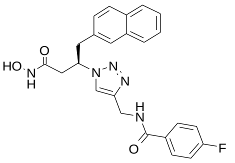<br>**1**	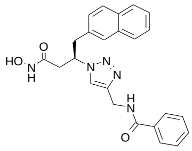<br>**2**	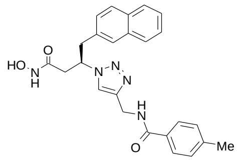<br>**3**	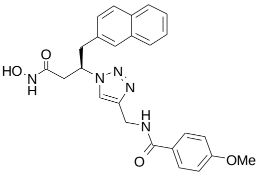<br>**4**	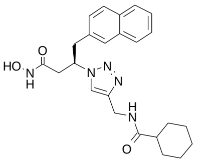<br>**5**
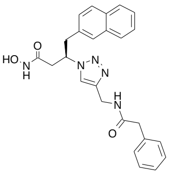<br>**6**	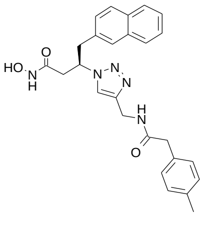<br>**7**	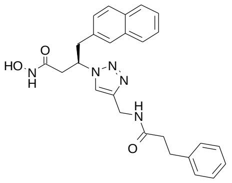<br>**8**	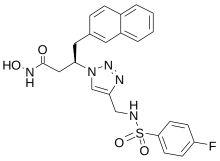<br>**9**	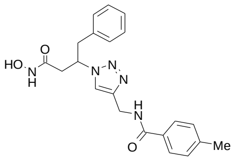<br>**10**
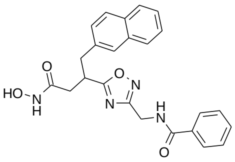<br>**11**	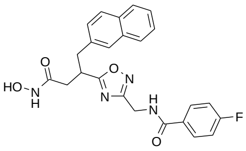<br>**12**	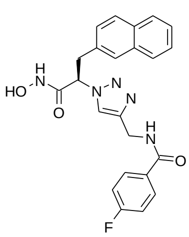<br>**13**	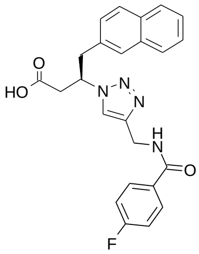<br>**14**	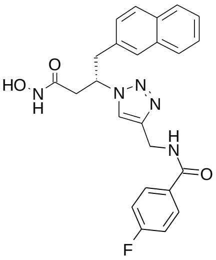<br>**15**
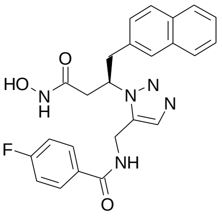<br>**16**	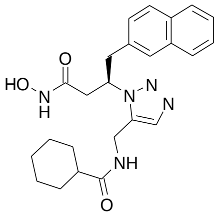<br>**17**	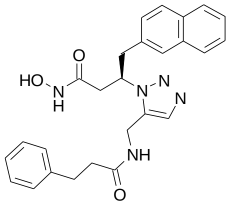<br>**18**	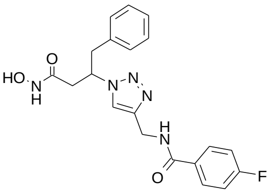<br>**19**	
**intermediates**				
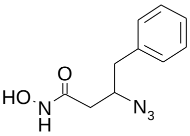<br>**20a**<br>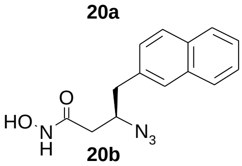<br>**20b**	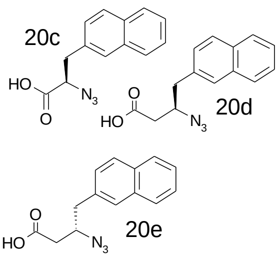<br>20c 20d 20e	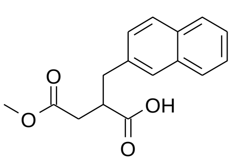<br>**21**	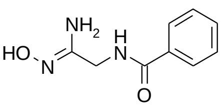<br>**22**	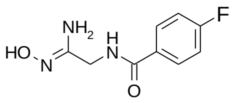<br>**23**

Supplementary Methods.

All reagents, solvents and starting materials were purchased from commercial suppliers and used without further purification. Melting points were determined using a Büchi B-540 melting point apparatus and are uncorrected. ^{1}H NMR spectra were recorded on a Brucker Avance 300 MHz spectrometer with methanol-*d6*, $CDCl_3$ or DMSO-*d6* as the solvent. ^{13}C NMR spectra are recorded at 100 MHz. All coupling constants are measured in hertz (Hz) and the chemical shifts (δ) are quoted in parts per million (ppm). Liquid chromatography mass spectroscopy analyses (LC–MS) were performed using LCMS-MS triple-quadrupole system (Waters) with a C_{18} TSK-GEL Super ODS (2 µm particle size column, 50 * 4.6 mm). LCMS gradient starting from 98% H_2O / 0.1% formic acid and reaching 2% H_2O / 98% MeOH within 5 min (method A) at a flow rate of 2 mL/min or starting from 100% H_2O / 0.1% formic acid and reaching 5% H_2O / 95% MeOH within 10 min (method B) at a flow rate of 1 mL/min was used. Purity (%) was determined by Reversed Phase HPLC, using UV detection (215 nM). High resolution mass spectroscopy (HRMS) were carried out on an Waters LCT Premier XE (TOF), ESI ionization mode, with a Waters XBridge C_{18} (150*4.6 mm, 3.5 µm particle size). LCMS gradient starting from 98% ammonium formate buffer 5 mM (pH 9.2) and reaching 95% CH_3CN / 5% ammonium formate buffer 5 mM (pH 9.2) within 15 min at a flow rate of 1 mL/min was used.

Synthesis of 1-10, 19 (Structures in Supplementary Table 6)

The azide **20a** or **20b** (1 equiv) and the alkyne (1equiv) were dissolved separately in DMSO (100-150µL) then added to a mixture of *t*BuOH/water (1/1) or directly solubilised in a mixture of DMF/water (1/1). $CuSO_4$:5H_2O (0.1equiv) and sodium ascorbate (1 equiv) were added. After 12 h of stirring at room temperature, the media was filtered. In most cases, concentration of the filtrate, followed by precipitation in water, filtration and washing with ethyl acetate gave a pure product. If necessary, the final triazole was purified by flash chromatography on silica gel (CH_2Cl_2/MeOH).

4-Fluoro-N-[1-(R-2-hydroxycarbamoyl-1-naphthalen-2-ylmethyl-ethyl)-1H-[1,2,3]triazol-4-ylmethyl]-benzamide (1). Beige solid (36 mg, 57%), Purity: 96%, mp 183.6-184.1 °C, LC t_R = 2.42 min, MS (ESI+): *m/z*=448 $(M+H)^+$. ^{1}H NMR (DMSO *d6*) δ: 10.50 (s, 0.5H, CONHOH), 8.99 (t, 0.9H, *J* = 5.4Hz,1H, CONH), 8,80 (s, 0.5H, CONHOH), 7.93-7.87 (m, 3H), 7.78-7.68 (m, 3H), 7.45-7.36 (m, 3H), 7.29 (t, 8.8Hz, 2H), 7.14 (d, *J* = 8,9Hz, 1H), 5.28-5.24 (m, 1H), 4.41 (d, *J* = 5.4Hz, 2H), 3.31 (m, 2H), 2.77 (dd, *J* = 8.7 and 14.9 Hz, 1H), 2.66 (dd, *J* = 5.4 and 14.9 Hz, 1H). ^{13}C NMR (DMSO *d6*) δ: 165.5, 165.0, 163.9 (d, $J_{C\text{-}F}$ = 247 Hz), 144.5, 135.0, 132.9, 132.3, 131.8, 130.7, 130.6, 130.0, 129.9, 127.7, 127.4 (2C), 127.3, 126.0, 125.6, 118.0, 115.2 (d, $J_{C\text{-}F}$ = 22 Hz), 58.8, 40.9, 37.1, 34.8. ^{19}F NMR (DMSO *d6*) δ: -109.99. HRMS *m/z* calculated for $C_{24}H_{23}FN_5O_3$ $(M+H)^+$ 448.1785, found 448.1801.

N-[1-((R)-2-Hydroxycarbamoyl-1-naphthalen-2-ylmethyl-ethyl)-1H-[1,2,3]triazol-4-ylmethyl]-benzamide (2). *B*eige solid (24 mg, 51%), Purity: 95%, mp 192.8-194.0 °C, LC t_R = 3.10 min, MS (ESI+): *m/z* = 430 $(M+H)^+$. ^{1}H NMR (DMSO *d6*) δ: 10.51 (s, 1H, CONHOH), 8.95 (t, *J* = 5.9Hz, 0.8H, CONHOH), 8,79 (s, 0.5H, CONHOH), 7.88-7.67 (m, 5H), 7.56-7.35 (m, 7H), 7.15 (dd, *J* = 1.3 and 8.6Hz, 1H), 5.28-5.26 (m, 1H), 4.42 (d, *J* = 5.6Hz, 2H), 3.32 (m, 2H), 2.77 (dd, *J* = 8.8 and 15.0 Hz, 1H), 2.65 (dd, *J* = 5.4 and 15.0 Hz, 1H). ^{13}C NMR (DMSO *d6*) δ: 166.5, 166.0, 145.1, 135.0, 134.6, 133.3, 132.3, 131.7 (2C), 128.7 (2C), 128.2, 127.9 (2C), 127.7 (3C), 126.5, 126.0, 122.9, 59.2, 41.4, 37.6, 35.2. HRMS *m/z* calculated for $C_{24}H_{24}N_5O_3$ $(M+H)^+$ 430.1879, found 430.1884.

N-[1-((R)-2-Hydroxycarbamoyl-1-naphthalen-2-ylmethyl-ethyl)-1H-[1,2,3]triazol-4-ylmethyl]-4-methyl-benzamide (3). White solid (25 mg, 66%), Purity: 100%, mp 181.7-182.6 °C, LC t_R = 3.22 min, MS (ESI+): *m/z*=444 $(M+H)^+$. ^{1}H NMR (DMSO *d6*) δ: 10.54 (s, 1H, CONHOH), 8.90 (t, *J* = 5.7Hz, 1H, CONH), 8,84 (s, 0.7H, CONHOH), 7.9 (s, 1H), 7.80-7.68 (m, 5H), 7.48-7.36 (m, 3H), 7.27 (d, *J* = 7.9Hz, 2H), 7.15 (d, *J* = 8.6Hz, 1H), 5.32-5.23 (m, 1H), 4.42 (d, *J* = 5.7Hz, 2H), 3.30 (d, *J* = 7.08 Hz, 2H), 2.79 (dd, *J* = 8.9 and 15.2 Hz, 1H), 2.66 (dd, *J* = 5.6 and 15.2 Hz, 1H), 2.36 (s, 3H). ^{13}C NMR (DMSO *d6*) δ: 166.4, 166.0, 145.1, 141.6, 135.0, 133.3, 132.3, 131.8, 129.3 (2C), 128.2 (2C), 127.9 (2C), 127.8 (3C), 126.5, 126.0, 123.0, 59.2, 41.1, 37.5, 35.2, 21.4. HRMS *m/z* calculated for $C_{25}H_{26}N_5O_3$ $(M+H)^+$ 444.2036, found 444.2041.

N-[1-((R)-2-Hydroxycarbamoyl-1-naphthalen-2-ylmethyl-ethyl)-1H-[1,2,3]triazol-4-ylmethyl]-4-methoxy-benzamide (4). Beige solid (43 mg, 68%), Purity: 96%, mp 178.3-179.6 °C, LC t_R = 2.33 min, MS (ESI+): *m/z*=460 $(M+H)^+$. ^{1}H NMR (DMSO *d6*) δ: 10.50 (s, 0.6H, CONHOH), 8,80 (m, 1.5 H, CONHOH + CONH), 7.86-7.69 (m, 6H), 7.47-7.39 (m, 3H), 7.15 (d, *J* = 8.6Hz, 1H), 6.98 (d, *J* = 8.6Hz, 2H), 5.26 (m, 1H), 4.40 (d, *J* = 5.3Hz, 2H), 3.81 (s, 3H), 3.31 (m, 2H), 2.77 (dd, *J* = 9.5 and 15.1 Hz, 1H), 2.65 (dd, *J* = 5.2 and 15.1 Hz, 1H). ^{13}C NMR (DMSO *d6*) δ: 166.0 (2C), 162.1, 145.2, 135.0, 133.3, 132.3, 129.6 (2C), 128.2 (2C), 127.9 (2C), 127.8, 126.8, 126.5, 126.0, 123.0, 113.9 (2C), 59.2, 55.8, 41.4, 37.6, 35.1. HRMS *m/z* calculated for $C_{25}H_{26}N_5O_4$ $(M+H)^+$ 460.1985, found 460.1977.

Cyclohexanecarboxylic acid [1-(R-2-hydroxycarbamoyl-1-naphthalen-2-ylmethyl-ethyl)-1H-[1,2,3]triazol-4-ylmethyl]-amide (5). Beige solid (25 mg, 51%), Purity: 95%, mp 176.6-177.9 °C, LC t_R = 2.48 min, MS (ESI+): *m/z*=436 $(M+H)^+$. ^{1}H NMR (DMSO *d6*) δ: 10.51 (s, 0.6H, CONHOH), 8,80 (s, 0.6H CONHOH), 8.07 (m, 1H, CONH), 7.82-7.72 (m, 4H), 7.49-7.46 (m, 3H), 7.15 (d, *J* = 7.9Hz, 1H), 5.27 (m, 1H), 4.17 (m, 2H), 3.32 (m, 2H), 2.81-2.63 (m, 2H), 2.07-2.03 (m, 1H), 1.65-1.59 (m, 5H), 1.22-1.15 (m, 5H). ^{13}C NMR (DMSO *d6*) δ: 175.6, 166.0, 135.0, 133.4, 132.3, 128.2 (2C), 127.9 (2C), 127.8, 127.7, 126.5, 126.1, 123.1, 59.2, 44.3, 41.4, 37.7, 34.4, 29.5 (2C) , 25.9, 25.7 (2C). HRMS *m/z* calculated for $C_{24}H_{30}N_5O_3$ $(M+H)^+$ 436.2349, found 436.2349.

***(R)-N-hydroxy-4-naphthalen-2-yl-3-[4-(phenylacetylamino-methyl)-[1,2,3]triazol-1-yl]-butyramide* (6)**. White solid (36 mg, 30%). Purity: 95%, LC tR= 2.34 min, MS (ESI+): *m/z* = 444 $[M + H]^+$. ^{1}H NMR (CD_3CN) δ: 9.08 (s, 1H, CONHOH), 7.85-7.74 (m, 3H), 7.48-7.42 (m, 4H), 7.34 (m, 6H), 6.88 (s, 1H), 5.28-5.17 (m, 1H), 4.25 (d, *J* = 5.7 Hz, 2H), 3.42-3.35 (m, 4H), 2.81-2.76 (m, 2H). ^{13}C NMR (CD_3CN) δ: 170.9, 166.4, 144.5, 135.9, 134.6, 134.5, 133.4, 132.3, 129.2, 128.5 (2C), 128.0, 127.7, 127.5 (2C), 127.2, 126.7, 126.2, 125.8, 122.4, 59.5, 42.6, 40.9, 37.7, 34.7. HRMS *m/z* calculated for $C_{25}H_{26}N_5O_3$ $(M+H)^+$ 444.2036, found 444.2024.

(R)-N-Hydroxy-4-naphthalen-2-yl-3-{4-[(2-p-tolyl-acetylamino)-methyl]-[1,2,3]triazol-1-yl}-butyramide (7). Yellow oil (21 mg, 55%), Purity: 90%, LC t_R = 2.52 min, MS (ESI+): *m/z* = 458 $(M+H)^+$. ^{1}H NMR (DMSO *d6*) δ: 10.52 (s, 0.6H, CONHOH), 8,81 (s, 0.7H, CONHOH), 8.41 (t, *J* = 5.0Hz, 0.8H, CONH), 7.85-7.74 (m, 4H), 7.49-7.44 (m, 3H), 7.15-7.04 (m, 5H), 5.26 (m, 1H), 4.20 (d, *J* = 5.2Hz, 2H), 3.31-3.25 (m, 4H), 2.76 (dd, *J* = 9.3 and 15.3 Hz, 1H), 2.65 (dd, *J* = 5.4 and 15.3Hz, 1H), 2.24 (s, 3H). ^{13}C NMR (DMSO *d6*) δ: 170.7, 166.1,

144.8, 135.9, 135.1, 133.7, 133.4, 132.4, 129.4, 129.3 (2C), 128.3, 127.9 (3C), 127.7 (2C), 126.6, 126.2, 122.9, 59.3, 42.2, 41.6, 37.8, 34.7, 21.1. HRMS *m/z* calculated for $C_{26}H_{28}N_5O_3$ $(M+H)^+$ 458.2192, found 458.2212.

(R)-N-Hydroxy-4-naphthalen-2-yl-3-{4-[(3-phenyl-propionylamino)-methyl]-[1,2,3]triazol-1-yl}-butyramide (8). Beige solid (43 mg, 82%), Purity: 95%, mp 150.0-151.4 °C, LC t_R = 2.50 min, MS (ESI+): *m/z* = 457 $(M+H)^+$. ^{1}H NMR (DMSO *d6*) δ: 10.52 (s, 0.7H, CONHOH), 8,81 (s, 0.7H, CONHOH), 8.27 (m, 1H, CONH), 7.83-7.67 (m, 4H), 7.50-7.42 (m, 3H), 7.28-7.16 (m, 6H), 5.25 (m, 1H), 4.20 (m, 2H), 3.32 (m, 2H), 2.80-2.73 (m, 2H), 2.78 (t, J=7.5 Hz, 2H), 2.35 (t, J=7.8 Hz, 2H). ^{13}C NMR (DMSO *d6*) δ: 171.6, 166.0, 141.8, 135.3, 135.1, 133.4, 132.3, 128.7 (2C), 128.6 (2C), 128.3, 127.9 (3C), 127.7, 126.5, 126.3 (2C), 126.1, 59.2, 41.4, 37.7, 37.3, 34.5, 31.5. HRMS *m/z* calculated for $C_{26}H_{28}N_5O_3$ $(M+H)^+$ 458.2192, found 458.2191.

(R)-3-{4-[(4-Fluoro-benzenesulfonylamino)-methyl]-[1,2,3]triazol-1-yl}-N-hydroxy-4-naphthalen-2-yl-butyramide (9). Beige solid (40 mg, 60%), Purity: 95%, mp 157.1-158.7 °C, LC t_R = 2.54 min, MS (ESI+): *m/z* = 484 $(M+H)^+$. ^{1}H NMR (DMSO *d6*) δ: 7.83-7.75 (m, 6H), 7.54-7.33 (m, 5H), 7.15 (d, *J*= 8.7Hz, 1H), 5.27-5.23 (m, 1H), 3.95 (s, 2H), 3.28-3.26 (m, 2H), 2.75 (dd, *J* = 9.1 and14.9Hz, 1H), 2.63 (dd, *J* = 5.6 and 14,9Hz, 1H). ^{13}C NMR (DMSO *d6*) δ: 166.0, 164.5 (d, J_{C-F} = 247 Hz), 137.1, 135.0, 133.4, 132.3, 130.1 (2C), 130.0, 128.3, 127.9 (3C), 127.7, 126.5, 126.1, 123.3, 116.7 (d, J_{C-F} = 22 Hz, 2C), 59.3, 41.3, 38.4, 37.5. HRMS *m/z* calculated for $C_{23}H_{23}FN_5O_4S$ $(M+H)^+$ 484.1455, found 484.1457.

N-[1-(1-Hydroxycarbamoylmethyl-2-phenyl-ethyl)-1H-[1,2,3]triazol-4-ylmethyl]-4-methyl-benzamide (10). White solid (20 mg, 54%), Purity: 97%, LC t_R = 2.10 min, MS (ESI+): *m/z* = 394 $(M+H)^+$. ^{1}H NMR (DMSO *d6*) δ: 8.89 (t, *J* = 5.6 Hz, 1H, CONH), 8.37 (s, 1H), 7.78 (s, 1H), 7.75 (d, *J* = 8.0 Hz, 2H), 7.26 (d, *J* = 8.0 Hz, 2H), 7.19-7.10 (m, 3H), 6.99-6.96 (m, 2H), 5.14 (m, 1H), 4.42 (d, *J* = 5.6Hz, 2H), 3.11 (d, *J* = 7.6 Hz, 2H), 2.72 (dd, *J* = 8.9 and 15.1 Hz, 1H), 2.58 (d, *J* = 5.5 and 15.1 Hz, 1H), 2.34 (s, 3H). ^{13}C NMR (DMSO *d6*) δ: 166.4, 165.9, 145.1, 141.6, 137.4, 131.8, 129.4, 129.2 (2C), 128.7 (2C), 127.8 (2C), 127.1 (2C), 123.0, 59.3, 41.3, 37.5, 35.2, 21.4. HRMS *m/z* calculated for $C_{21}H_{24}N_5O_3$ $(M+H)^+$ 394.1879, found 394.1892.

N-[1-(1-Hydroxycarbamoylmethyl-2-phenyl-ethyl)-1H-[1,2,3]triazol-4-ylmethyl]-4-fluoro-benzamide (19). White solid (15 mg, 15%)., Purity: 98%, LCt_R = 2.13 min, MS (ESI +): *m/z*= 398 $(M+H)^+$. ^{1}H NMR (MeOD, *d6*) δ: 7.68 (m, 2H), 7.59 (s, 1H, CONHOH), 7.19 (m, 1H), 7.14 (t, J= 0.06 Hz, 2H), 7.08 (m, 1H), 6.98 (dd, J_1= 0.03Hz, J_2= 0.004 Hz), 5.17 (m, 1H), 4.53 (s, 2H), 3.32 (m, 3H), 3.23 (m, 2H), 2.86 (m, 2H). ^{13}C NMR (MeOD, *d6*) δ: 167.4, 167.1, 164.9 (d, *J* = 250 Hz), 163.2, 144.2, 136.5, 130.3, 129.7, 129.5, 128.6 , 128.3, 126.7, 123.5, 115.1, 114.8, 60.1, 47.0, 40.9, 37.4, 34.6. HRMS *m/z* calculated for $C_{20}H_{20}FN_5O_3$ $(M+H)^+$ calculated 398,4125 found 398,1625.

Synthesis of 11 and 12

2-naphthalen-2-ylmethyl-succinic acid 4-methyl ester (**21**). To a refluxing suspension of *t*-BuOK (2.59 g, 23.1 mmol, 1.2 eq) in *t*-BuOH (17 mL) was carefully added a solution of dimethyl succinate (3.3 mL, 25.0 mmol, 1.3 eq) and 2-naphtaldehyde (3.00 g, 19.2 mmol, 1 eq) in *t*-BuOH (17 mL). The reaction mixture was stirred at reflux temperature for 3 h, after which the solvent was removed under vacuum. The residue was dissolved in 1 M HCl (17 mL) and this solution was extracted with EtOAc (3×50 mL). The organic layers were dried ($MgSO_4$)

and concentrated. The resulting monoacid was dissolved and EtOH (24 mL) and aqueous NaOH (2 M, 48 mL) were added. The resultant mixture was stirred under reflux for 16 h, followed by evaporation of the EtOH under reduced pressure. Extra H_2O (60 mL) and NaOH (2 M, 10 mL) was added and the mixture was washed with EtOAc (3×60 mL). Next, the aqueous layer was acidified (pH≈1) with 1 M HCl, extracted with EtOAc (2×120 mL) and the organic layers were dried ($MgSO_4$) and concentrated. The resulting diacid was dissolved in MeOH (7.4 mL), Amberlyst-15H^+ (1.3 g) was added and the reaction mixture was heated under reflux for 16 h. The mixture was filtered over Celite and concentrated under vacuum, resulting in crude ester. The product was purified by flash chromatography (CH_2Cl_2/MeOH 100:0 to 98:2 (v/v)) to give compound *2-naphthalen-2-ylmethylene-succinic acid 4-methyl ester* as a white amorphous solid (2.067 g, 40%). Purity: 75%, LC tR= 3.09 min (method A), MS (ESI+): *m/z*= 271 $[M + H]^+$. ^1H NMR ($CDCl_3$) δ: 8.21 (br s, 1H, O<u>H</u>), 7.89-7.86 (m, 4H), 7.58-7.47 (m, 3H), 3.80 (s, 3H), 3.67 (s, 2H). ^{13}C NMR ($CDCl_3$) δ: 172.8, 171.6, 144.4, 133.4, 133.1, 132.1, 129.3, 128.5, 127.7, 127.2, 126.7, 126.2, 125.3, 52.3, 33.3. Under an atmosphere of argon, Pd/C (0.35 g) and ammonium formate (1.64 g) was added to a solution of the previous acid (0.780 g, 2.89 mmol) in ethanol (29 mL). The mixture was stirred at room temperature for 16 h, then it was filtrated over Celite. Solvent was removed under vacuum. The residue was solubilized in water and acidified (pH≈1) with 1 M HCl, extracted with CH_2Cl_2 (2×120 mL) and the collected organic layers were dried ($MgSO_4$) and concentrated. The residue was purified by flash chromatography (CH_2Cl_2/MeOH 100:0 to 98:2 (v/v)) to give compound **21** as a yellow oil (0.663 g, 84%). Purity: 84%, LC tR= 2.65 min (method A), MS (ESI-): *m/z*= 271 $[M - H]^-$. ^1H NMR (MeOD) δ: 9.74 (br s, 1H, O<u>H</u>), 7.89-7.84 (m, 3H), 7.70 (s, 1H), 7.54-7.48 (m, 2H), 7.39 (d, *J* = 8.4 Hz, 1H), 3.66 (s, 3H), 3.37-3.32 (m, 2H), 3.00 (dd, *J* = 10.5 and 15.3 Hz, 1H), 2.75 (dd, *J* = 8.7 and 17.1 Hz, 1H), 2.51 (dd, *J* = 4.5 and 17.1 Hz, 1H). ^{13}C NMR (MeOD) δ: 180.1, 172.2, 135.7, 133.5, 132.4, 128.3, 127.7, 127.6, 127.6, 127.2, 126.2, 125.7, 42.9, 37.5, 34.6.

General procedure for the chemical synthesis of the N-*hydroxy amidines* ***22-23***. Aminoacetonitrile hydrochloride (1 eq) was placed in a flask where pyridine was carefully added to obtain a solution, and to this was added acyl chloride (1.05 eq) dropwise over 20 min. After stirring overnight at room temperature, water was carefully added; pyridinium hydrochloride dissolved while the product precipitated as a white solid. The precipitate was collected by filtration and washed with water. If no precipitation was observed, the mixture was extracted three times with dichloromethane. The combined organic layers were dried over $MgSO_4$, filtrated and concentrated under reduced pressure to give the cyanomethylbenzamide as a solid. To a solution of the obtained cyanomethylbenzamide (1.0 equiv) in methanol cooled to 0 °C were added hydroxylamine hydrochloride (1.0 equiv) and triethylamine (1.0 equiv), and the mixture was stirred at room temperature or heated to reflux overnight. The mixture was concentrated under reduced pressure. Water was added to the residue and the solid was collected by filtration, washed with water and dried to give the *N*-hydroxy amidine product as a white amorphous solid.

N-*(*N-*hydroxycarbamimidoylmethyl)-benzamide (**22**)* (yield = 80%). Purity: 93%, LC tR= 1.37 min (method A), MS (ESI+): *m/z*= 194 $[M + H]^+$. ^1H NMR (DMSO-*d6*) δ: 9.04 (br s, 1H, O<u>H</u>), 8.70 (br s, 1H, N<u>H</u>), 7.86 (d, *J* = 7.2 Hz, 2H), 7.53-7.46 (m, 3H), 5.38 (br s, 2H, N<u>H₂</u>), 3.86 (d, *J* = 5.4 Hz, 2H). ^{13}C NMR (DMSO-*d6*) δ: 167.2, 151.0, 134.5, 131.8, 128.8, 127.7, 46.1.

*4-fluoro-*N-*(*N-*hydroxycarbamimidoylmethyl)-benzamide (**23**)* (yield = 95%). Purity: 100%, LC tR= 1.37 min (method A), MS (ESI+): *m/z*= 212 $[M + H]^+$. ^1H NMR (DMSO-*d6*) δ: 9.05 (br s, 1H, O<u>H</u>), 8.78 (br t, *J* = 6.0 Hz,

1H, N$\underline{H}$), 7.97-7.92 (m, 2H), 7.29 (t, J = 9.0 Hz, 2H), 5.39 (br s, 2H, N$\underline{H}_2$), 3.85 (d, J = 6.0 Hz, 2H). ^{13}C NMR (DMSO-*d6*) δ: 166.1, 162.7, 150.9, 131.0, 130.4 (d, J_{C-F} = 9 Hz), 115.6 (d, J_{C-F} = 22 Hz), 45.9.

To a solution of **21** (1 eq) in DCM was added carbonyldiimidazole (2.2 eq). The reaction mixture was stirred at room temperature for 10 min. N-hydroxy amidine (1 eq) (**22 or 23**) was added and the solution was stirred at room temperature for 16 h. The solvent was removed by evaporation in vacuo, and the residue was dissolved in EtOAc and washed twice with water. The organic layer was dried ($MgSO_4$) and the solvent was removed by evaporation in vacuo. The residue was solubilized in DMF and the solution was heated to reflux for 4 h. The reaction mixture was cooled to room temperature, the solvent was removed by evaporation in vacuo, and the residue was dissolved in EtOAc and washed twice with water. The organic layer was dried ($MgSO_4$) and the solvent was removed by evaporation in vacuo. The oil was purified by flash chromatography on silica gel (cyclohexane/EtOAc 1:0 to 1:1 (v/v)) to afford oxadiazole as a colorless oil. The resulting ester was dissolved and EtOH (24 mL) and aqueous NaOH (2 M) was added. The resultant mixture was stirred under reflux for 16 h, followed by evaporation of the EtOH under reduced pressure. H_2O (60 mL) was added and the mixture was washed with EtOAc (3×60 mL). Next, the aqueous layer was acidified (pH≈1) with 1 M HCl, extracted with EtOAc (2×120 mL) and the organic layers were dried ($MgSO_4$) and concentrated. The resulting acid (0.121 g, 0.30 mmol) was dissolved in DMF (5.9 mL). EDCI (0.120 g, 0.74 mmol), HOBt (0.229 g, 0.92 mmol), N-methylmorpholine (0.41 mL, 3.70 mmol) were added. The mixture was stirred at room temperature for 5 min, and *O*-tritylhydroxylamine (0.204 g, 0.74 mmol) was added. The mixture was stirred at room temperature overnight. The solvent was evaporated under reduced pressure and the residue was dissolved in CH_2Cl_2 and washed three times with a 5% $NaHCO_3$ (aq) solution and once with water. The organic layer was dried with $MgSO_4$, filtrated and concentrated under reduced. The crude product was purified by flash chromatography on silica gel (cyclohexane/EtOAc 1:0 to 1:1 (v/v)) to afford the *O*-trityl hydroxamate. The *O*-trityl hydroxamate intermediate was dissolved in TFA 5%/CH_2Cl_2 (0.46 mL) and triisopropylsilane (0.02 mL) was added. The mixture was stirred at room temperature for 15 min. Solvents were removed under reduced pressure and the residue was triturated with diethyl ether and petroleum ether to give a residue which was purified by flash chromatography on silica gel (CH_2Cl_2/MeOH 10:0 to 95:5 (v/v)).

N-[5-(1-hydroxycarbamoylmethyl-2-naphthalen-2-yl-ethyl)-[1,2,4]oxadiazol-3-ylmethyl]-benzamide (11) colorless oil (0.065 g, 41%). Purity: 93%, LC tR= 2.87 min (method A), MS (ESI+): *m/z*= 431 $[M + H]^+$. ^{1}H NMR (acetone-*d6*) δ: 8.29 (br s, 1H, N$\underline{H}$), 7.95-7.75 (m, 5H), 7.61 (s, 1H), 7.55-7.42 (m, 5H), 7.29-7.27 (m, 1H), 4.66 (s, 2H), 3.96-3.93 (m, 1H), 3.24 (d, J = 6.6 Hz, 2H), 2.66-2.62 (m, 2H). ^{13}C NMR (acetone-*d6*) δ: 181.5, 168.2, 167.0, 166.6, 135.5, 134.3, 133.6, 132.4, 131.4, 128.4 (2C), 128.0, 127.6 (2C), 127.5, 127.3 (3C), 126.0, 125.6, 38.8, 36.4, 35.1, 34.6. HRMS *m/z* calculated for $C_{24}H_{23}N_4O_4$ $(M+H)^+$ 431.1719, found 431.1727.

4-fluoro-N-[5-(1-hydroxycarbamoylmethyl-2-naphthalen-2-yl-ethyl)-[1,2,4]oxadiazol-3-ylmethyl]-benzamide (12) colorless oil (0.044 g, 28%). Purity: 100%, LC tR= 2.90 min (method A), MS (ESI+): *m/z*= 449 $[M + H]^+$. ^{1}H NMR (acetone-*d6*) δ: 8.35 (br t, 1H, N$\underline{H}$), 8.02-7.98 (m, 2H), 7.82-7.74 (m, 3H), 7.60 (s, 1H), 7.46-7.40 (m, 2H), 7.29-7.20 (m, 3H), 4.65 (d, J = 6.0 Hz, 2H), 4.00-3.90 (m, 1H), 3.24 (d, J = 7.2 Hz, 2H), 2.65 (t, J = 8.7 Hz, 2H). ^{13}C NMR (acetone-*d6*) δ: 181.5, 168.1, 167.1, 165.7, 164.6 (d, J_{C-F} = 248 Hz), 163.7, 135.5,

133.5, 132.4, 130.7, 130.0, 129.9, 128.0, 127.6 (2C), 127.5, 127.3, 126.0, 125.6, 115.2 (d, J_{C-F} = 22 Hz), 38.8, 36.4, 35.1, 34.6. HRMS *m/z* calculated for $C_{24}H_{22}N_4O_4F$ $(M+H)^+$ 449.1625, found 449.1668.

Synthesis of compounds 13-15.

The azide **20c-e** (1 equiv) and the alkyne (1equiv) were dissolved separately in DMSO (100-150µL) then added to a mixture of *t*BuOH/water (1/1) or directly solubilised in a mixture of DMF/water (1/1). $CuSO_4$:$5H_2O$ (0.1equiv) and sodium ascorbate (1 equiv) were added. After 12 h of stirring at room temperature, the media was filtered. In most cases, concentration of the filtrate, followed by precipitation in water, filtration and washing with ethyl acetate gave a pure product. If necessary, the final triazole was purified by flash chromatography on silica gel (CH_2Cl_2/MeOH).

For the synthesis of **13** and **15**, the carboxylic acid intermediate (0.65 mmol) was dissolved in DMF (13.0 mL). EDCI (0.225 g, 1.62 mmol), HOBt (0.503 g, 2.01 mmol), N-methylmorpholine (0.89 mL, 8.12 mmol) were added. The mixture was stirred at room temperature for 5 min, and *O*-tritylhydroxylamine (0.447 g, 1.62 mmol) was added. The mixture was stirred at room temperature overnight. The solvent was evaporated under reduced pressure and the residue was dissolved in CH_2Cl_2 and washed three times with a 5% $NaHCO_3$ (aq) solution and once with water. The organic layer was dried with $MgSO_4$, filtrated and concentrated under reduced. The crude product was purified by flash chromatography on silica gel (dichloromethane/MeOH 10:0 to 95:5 (v/v)) to afford the hydroxamates. *O*-trityl hydroxamate intermediate was dissolved in TFA 5%/CH_2Cl_2 (6.6 mL) and triisopropylsilane (0.72 mL) was added. The mixture was stirred at room temperature for 30 min. Solvents were removed under reduced pressure and the residue was triturated with diethyl ether and petroleum ether to give a residue which was purified by flash chromatography on silica gel (CH_2Cl_2/MeOH 10:0 to 95:5 (v/v))

(R)-4-Fluoro-N-[1-(1-hydroxycarbamoyl-2-naphthalen-2-yl-ethyl)-1H-[1,2,3]triazol-4-ylmethyl]-benzamide (13). White solid (65 mg, 11%), Purity: 96%, LC t_R = 2.77 min (method A), MS (ESI+): *m/z* = 434 $[M+H]^+$. ^1H NMR (DMSO-*d6*) δ: 9.08 (t, *J* = 5.0 Hz, 1H), 8.22 (s, 1H), 8.00-7.95 (m, 2H), 7.85-7.77 (m, 2H), 7.73-7.70 (m, 1H), 7.64 (s, 1H), 7.47-7.40 (m, 2H), 7.38-7.29 (m, 3H), 5.45 (t, *J* = 8.0 Hz,, 1H), 4.49 (d, *J* = 5.4 Hz, 2H), 3.54-3.52 (m, 2H). ^{13}C NMR (DMSO) δ: 165.5, 164.4 (J_{C-F} = 247 Hz), 164.3, 145.5, 134.2, 133.3, 132.4, 131.1 (J_{C-F} = 3 Hz), 130.5, 130.4, 128.3, 128.0, 127.9, 127.8, 127.7, 126.5, 126.2, 122.5, 115.7 (J_{C-F} = 22 Hz, 2C), 62.3, 38.0, 35.3. HRMS *m/z* calculated for $C_{23}H_{21}N_5O_3F$ $(M+H)^+$ 434.1628, found 448.1588.

(R)-3-{4-[(4-fluoro-benzoylamino)-methyl]-[1,2,3]triazol-1-yl}-4-naphthalen-2-yl-butyric acid (14) White solid (1.110 g, Quant.). Purity: 100%, LC t_R = 2.47 min (method A), MS (ESI+): *m/z* = 433 $[M+H]^+$. ^1H NMR (DMSO-*d6*): 12.28 (br s, 0.6H, COOH), 9.00 (t, 1H, *J* = 5.1 Hz,1H, CONH), 8,00 (s, 1H), 7.95-7.90 (m, 2H), 7.80-7.68 (m, 3H), 7,49 (s, 1H), 7.45-7.37 (m, 2H), 7.30 (t, *J* = 8.7 Hz, 2H), 7.14 (d, *J* = 8,1 Hz, 1H), 5.21 (br s, 1H), 4.44 (d, *J* = 5.4 Hz, 2H), 3.37-3.25 (m, 2H + H_2O), 3.08-2.97 (m, 2H), 2.66 (dd, *J* = 5.4 and 14.9 Hz, 1H). ^{13}C NMR (DMSO-*d6*): 166.0, 165.4, 164.4 (d, J_{C-F} = 247 Hz), 145.1, 135.0, 133.3, 132.3, 131.1, 130.5 (2C), 130.3, 128.2, 127.9 (3C), 126.5, 126.1, 122.9, 115.7 (d, J_{C-F} = 22 Hz, 2C), 59.3, 41.4, 35.3 (2C). HRMS *m/z* calculated for $C_{24}H_{22}N_4O_3F$ $(M+H)^+$ 433.1676, found 433.1667.

***(S)-4-fluoro-N-[1-(2-hydroxycarbamoyl-1-naphthalen-2-ylmethyl-ethyl)-1H-[1,2,3]triazol-4-ylmethyl]-benzamide* (15)** White solid (0.255 g, 46%). Purity: 96 %, LC tr = 2.75 min, MS (ESI+): *m/z* = 448 $[M+H]^+$. ^1H NMR (DMSO-*d6*) δ: 10.51 (s, 0.9H), 8.99 (br t, *J* = 5.7 Hz, 1H), 8.79 (br s, 0.9H), 7.93-7.88 (m, 3H), 7.78-7.70 (m, 3H), 7.47-7.40 (m, 3H), 7.30 (t, *J* = 8.7 Hz, 2H), 7.14 (d, *J* = 8.4 Hz, 1H), 5.31-5.22 (m, 1H), 4.42 (d, *J* = 5.7

Hz, 2H), 3.32-3.28 (m, H_2O+2H), 2.78 (dd, J = 8.7 and 15.0 Hz, 1H), 2.66 (dd, J = 5.4 and 15.0 Hz, 1H). ^{13}C NMR (DMSO-*d6*) δ: 166.0, 165.4, 164.3 (d, J_{C-F} = 246 Hz), 144.9, 135.0, 133.3, 132.3, 131.1, 130.4 (2C), 130.3, 128.2, 127.8 (2C), 127.7, 126.5, 126.0, 123.0, 115.7 (d, J_{C-F} = 21 Hz, 2C), 59.2, 41.4, 37.6, 35.2. Mp = 190 °C.HRMS *m/z* calculated for $C_{24}H_{23}N_5O_3F$ $[M+H]^+$ 448.1785, found 448.1761.

Synthesis of compounds 16-18

(R)-3-(5-Aminomethyl-[1,2,3]triazol-1-yl)-4-naphthalen-2-yl-butyric acid

(R)-3-Azido-4-naphthalen-2-yl-butyric acid (**19d**) (1.85 g, 7.25 mmol) and propargylamine (0.56 mL, 8.70 mmol) were dissolved in EtOAc (55 mL), and the resulting solution was stirred at room temperature for 10 min. DMTMM (2.41 g, 8.70 mmol) was added and the reaction was stirred at room temperature for 3 h. The mixture was washed twice with 1 M HCl (aq), twice with saturated $NaHCO_3$ (aq) and twice with saturated NaCl (aq), dried over $MgSO_4$, filtered and concentrated under reduced pressure. The residue was purified by flash chromatography on silica gel using CH_2Cl_2 as eluent. *(R)-3-Azido-4-naphthalen-2-yl-N-prop-2-ynyl-butyramide* was obtained as a yellowish oil (1.270 g, 60%). Purity: 90%, LC tR= 2.85 min (method A), MS (ESI+): *m/z* =293 $[M + H]^+$. ^{1}H NMR ($CDCl_3$) δ: 7.85-7.80 (m, 3H), 7.69 (s, 1H), 7.52-7.45 (m, 2H), 7.37 (dd, J = 1.6 and 8.4 Hz, 1H), 5.80 (sl, 1H), 4.29-4.23 (m, 1H), 4.15-3.99 (m, 2H), 3.06 (d, J = 6.8 Hz, 1H), 2.45 (dd, J = 4.5 and 14.9 Hz, 1H), 2.31 (dd, J = 8.6 and 14.9 Hz, 1H), 2.25 (t, J = 2.5 Hz, 2H). ^{13}C NMR ($CDCl_3$) δ: 169.2, 134.2, 133.5, 132.5, 128.4, 128.2, 127.7, 127.6, 127.4, 126.3, 125.8, 79.1, 71.9, 60.3, 40.7, 29.3. The azido compound (1.25 g, 4.28 mmol) was dissolved in DMF (110 mL) and was heated to reflux and left stirring overnight to allow cyclisation. After cooling down the mixture was diluted with EtOAc and was washed with H_2O (3 times). The organic phase was dried with $MgSO_4$ and evaporated under reduced pressure to give *R)-8-Naphthalen-2-ylmethyl-4,5,7,8-tetrahydro-1,2,5,8a-tetraaza-azulen-6-one* (1.05 g, 84%) as a beige solid. Purity: 95%, LC tR= 2.24 min (method A), MS (ESI+): *m/z* =293 $[M + H]^+$. ^{1}H NMR ($CDCl_3$) δ: 7.84-7.78 (m, 3H), 7.66 (s, 1H), 7.52-7.45 (m, 3H), 7.27 (dd, J = 1.6 and 8.4 Hz, 2H), 6.65 (sl, 1H), 5.21-5.18 (m, 1H), 4.38 (dd, J = 5.3 and 17.0 Hz, 1H), 4.27 (dd, J = 6.0 and 17.0 Hz, 1H), 3.71 (dd, J = 3.3 and 13.6 Hz, 1H), 3.55 (dd, J = 8.8 and 13.6 Hz, 1H), 2.98-2.96 (m, 2H). ^{13}C NMR ($CDCl_3$) δ: 172.3, 133.4, 132.8, 132.7, 132.6, 131.2, 128.9, 128.5, 127.7, 127.6, 127.6, 126.3, 126.0, 58.0, 42.2, 35.5. A 6 M solution of HCl (aq) (10.5 mL) was added to (R)-8-Naphthalen-2-ylmethyl-4,5,7,8-tetrahydro-1,2,5,8a-tetraaza-azulen-6-one (0.250 g, 0.86 mmol) and splitted in four microwaves tubes. The mixture was heated under microwave conditions at 85 °C for 1 h (Discover – CEM, Method standard: power max 200 W, ramp time 20 min, hold time 60 min, T 85 °C, internal pressure max 20 bar). The solution was evaporated under reduced pressure to give compound *(R)-3-(5-Aminomethyl-[1,2,3]triazol-1-yl)-4-naphthalen-2-yl-butyric acid* (322 mg, quant.) as an orange solid. Purity: 97%, LC tR= 1.90 min (method A), MS (ESI+): *m/z* = 311 $[M + H]^+$. ^{1}H NMR (DMSO-d_6) δ: 8.49 (sl, 2H), 7.87-7.76 (m, 3H), 7.66 (s, 1H), 7.58 (s, 1H), 7.48-7.45 (m, 2H), 7.28 (dd, J = 1.6 and 8.4 Hz, 2H), 5.26-5.13 (m, 1H), 3.95 (dd, J = 6.3 and 15.8 Hz, 1H), 4.27 (dd, J = 5.2 and 15.8 Hz, 1H), 3.44 (dd, J = 6.3 and 14.0 Hz, 1H), 3.32 (dd, J = 8.6 and 14.0 Hz, 1H), 3.19 (dd, J = 9.3 and 17.3 Hz, 1H), 3.06 (dd, J = 4.5 and 17.3 Hz, 1H). ^{13}C NMR (DMSO-d_6) δ: 172.3, 134.8, 133.3, 133.2, 132.4, 132.1, 128.4, 128.2, 128.0, 127.8, 126.7, 126.2, 56.6, 41.4, 31.7.

***(R)-4-Fluoro-N-[3-(1-hydroxycarbamoylmethyl-2-naphthalen-2-yl-ethyl)-3H-[1,2,3]triazol-4-ylmethyl]-benzamide* (16).** *(R)-3-(5-Aminomethyl-[1,2,3]triazol-1-yl)-4-naphthalen-2-yl-butyric acid* (0.300 mg, 0.97 mmol) was dissolved in dioxane (2.8 mL) and an aqueous saturated solution of K_2CO_3 (5.7 mL). The biphasic

mixture was cooled to 0 °C. A solution of 4-fluoro-benzoyl chloride (0.114 mL, 0.97 mmol) in dioxane (2.8 mL) was slowly added to the amino acid solution and the reaction was allowed to slowly warm to room temperature. After 3 hours, the reaction was dissolved in water and extracted with diethyl ether (twice). The resulting aqueous layer was acidified to pH 1 with 3 M HCl (aq) and extracted with ethyl acetate (3 times). The combined organic layers were dried with $MgSO_4$, filtered and concentrated under reduced pressure to give (R)-3-{5-[(4-Fluoro-benzoylamino)-methyl]-[1,2,3]triazol-1-yl}-4-naphthalen-2-yl-butyric acid as a yellowish solid (435 mg, quant.). Purity: 61%, LC tR= 2.55 min (method A), MS (ESI+): *m/z* =433 $[M + H]^+$. (R)-3-{5-[(4-Fluoro-benzoylamino)-methyl]-[1,2,3]triazol-1-yl}-4-naphthalen-2-yl-butyric acid (0.435 g, 1.01 mmol) was dissolved in DMF (20 mL). EDCI (0.348 g, 2.51 mmol), HOBt (0.779 g, 3.12 mmol), N-methylmorpholine (1.38 mL, 12.57 mmol) were added. The mixture was stirred at room temperature for 5 min, and *O*-tritylhydroxylamine (0.692 g, 2.51 mmol) was added. The mixture was stirred at room temperature overnight. The solvent was evaporated under reduced pressure and the residue was dissolved in CH_2Cl_2 and washed three times with a 5% $NaHCO_3$ (aq) solution and once with water. The organic layer was dried with $MgSO_4$, filtered and concentrated under reduced. The crude product was purified by flash chromatography on silica gel (cyclohexane/EtOAc 1:0 to 1:1 (v/v)) to afford (R)-4-Fluoro-N-{3-[2-naphthalen-2-yl-1-(trityloxycarbamoyl-methyl)-ethyl]-3H-[1,2,3]triazol-4-ylmethyl}-benzamide as a white amorphous solid (0.42 g, 6%). Purity: 88%, LC tR= 3.47 min (method A), MS (ESI+): *m/z* = 690 $[M + H]^+$. *O*-trityl hydroxamate intermediate was dissolved in TFA 5%/CH_2Cl_2 (0.57 mL) and triisopropylsilane (0.03 mL) was added. The mixture was stirred at room temperature for 2 h. Solvents were removed under reduced pressure and he residue was triturated with diethyl ether and petroleum ether to give a residue which was purified by flash chromatography on silica gel (CH_2Cl_2/MeOH 10:0 to 95:5 (v/v)) to afford compound **16** as a white solid (0.012 g, 3 steps 3%). Purity: 97%, LC tR= 2.42 min (method A), MS (ESI+): *m/z* =448 $[M + H]^+$. ^1H NMR (CD_3CN) δ: 9.21 (s, 0.8H, CONH), 7.86-7.68 (m, 6H), 7.47-7.40 (m, 4H), 7.13 (t, *J* = 8.9 Hz, 3H), 5.33-5.19 (m, 1H), 4.43 (dd, *J* = 6.8 and 15.0 Hz, 1H), 3.86 (dd, *J* = 3.7 and 15.0 Hz, 1H), 3.46-3.43 (m, 2H), 3.13 (dd, *J* = 10.5 and 15.5 Hz, 1H), 2.92 (dd, *J* = 4.1 and 15.5 Hz, 1H). ^{13}C NMR (CD_3CN) δ: 167.0, 165.5, 164.6 (d, *J* = 247 Hz), 146.2, 135.0, 134.7, 133.3, 132.5, 130.2, 129.9, 129.8, 128.0, 127.7, 127.5, 127.4, 127.1, 126.2, 125.8, 115.2 (d, J_{C-F} = 21.8 Hz, 2C), 56.9, 41.4, 37.9, 31.3. HRMS *m/z* calculated for $C_{24}H_{23}N_5O_3F$ $(M+H)^+$ 448.1785, found 448.1788.

***(R)-Cyclohexanecarboxylic acid [3-(1-hydroxycarbamoylmethyl-2-naphthalen-2-yl-ethyl)-3H-[1,2,3]triazol-4-ylmethyl]-amide* (*17*).** *(R)-3-(5-Aminomethyl-[1,2,3]triazol-1-yl)-4-naphthalen-2-yl-butyric acid* (0.375 mg, 1.21 mmol) was dissolved in dioxane (3.6 mL) and an aqueous saturated solution of K_2CO_3 (7.1 mL). The biphasic mixture was cooled to 0 °C. A solution of cyclohexanecarbonyl chloride (0.162 mL, 1.21 mmol) in dioxane (3.6 mL) was slowly added to the amino acid solution and the reaction was allowed to slowly warm to room temperature. After 4 hours, the reaction was dissolved in water and extracted with diethyl ether (twice). The resulting aqueous layer was acidified to pH 1 with 3 M HCl (aq) and extracted with ethyl acetate (3 times). The combined organic layers were dried with $MgSO_4$, filtered and concentrated under reduced pressure to give (R)-3-{5-[(Cyclohexanecarbonyl-amino)-methyl]-[1,2,3]triazol-1-yl}-4-naphthalen-2-yl-butyric acid as an orange oil (449 mg, 88%). Purity: 83%, LC tR= 2.59 min (method A), MS (ESI+): *m/z* =421 $[M + H]^+$. (R)-3-{5-[(Cyclohexanecarbonyl-amino)-methyl]-[1,2,3]triazol-1-yl}-4-naphthalen-2-yl-butyric acid (0.449 g, 1.07 mmol) was dissolved in DMF (21 mL). EDCI (0.369 g, 2.67 mmol), HOBt (0.826 g, 3.31 mmol), N-methylmorpholine (1.47 mL, 13.35 mmol) were added. The mixture was stirred at room temperature for 5 min, and *O*-tritylhydroxylamine (0.735 g, 2.67 mmol) was added. The mixture was stirred at room temperature overnight.

The solvent was evaporated under reduced pressure and the residue was dissolved in CH_2Cl_2 and washed three times with a 5% $NaHCO_3$ (aq) solution and once with water. The organic layer was dried with $MgSO_4$, filtered and concentrated under reduced. The crude product was purified by flash chromatography on silica gel (cyclohexane/EtOAc 1:0 to 1:1 (v/v)) to afford (R)- Cyclohexanecarboxylic acid {3-[2-naphthalen-2-yl-1-(trityloxycarbamoyl-methyl)-ethyl]-3H-[1,2,3]triazol-4-ylmethyl}-amide as a white amorphous solid (0.117 g, 14%). Purity: 84%, LC tR = 3.52 min (method A), MS (ESI+): *m/z* = 678 $[M + H]^+$. *O*-trityl hydroxamate intermediate was dissolved in TFA 5%/CH_2Cl_2 (2.3 mL) and triisopropylsilane (0.08 mL) was added. The mixture was stirred at room temperature for 2 h. Solvents were removed under reduced pressure and he residue was triturated with diethyl ether and petroleum ether to give a residue which was purified by flash chromatography on silica gel (CH_2Cl_2/MeOH 10:0 to 95:5 (v/v)) to afford compound **17** as a white solid (0.041 g, 3 steps 8%). Purity: 98%, LC tR= 2.47 min (method A), MS (ESI+): *m/z* =436 $[M + H]^+$. ^1H NMR (CD_3CN) δ: 9.29 (s, 0.7H), 7.85-7.75 (m, 3H), 7.49-7.46 (m, 3H), 7.31 (s, 1H), 7.17 (d, *J* = 8.3 Hz, 1H), 7.31 (s, 1H), 6.69 (sl, 1H), 5.23-5.08 (m, 1H), 3.97 (sl, 2H), 3.69-3.47 (m, 1H), 3.43 (d, *J* = 7.3 Hz, 2H), 3.15-2.88 (m, 3H), 1.64 (d, *J* = 12.2 Hz, 5H), 1.29-1.18 (m, 5H). ^{13}C NMR (CD_3CN) δ: 176.3, 166.9, 146.0, 136.0, 134.7, 133.3, 132.3, 131.9, 128.1, 127.8, 127.6, 127.5, 127.2, 126.3, 125.9, 57.1, 44.4, 41.2, 38.0, 31.1, 29.1, 25.5 (2C), 25.3. HRMS *m/z* calculated for $C_{24}H_{30}N_5O_3$ $(M+H)^+$ 436.2349 found 430.2353.

***(R)-N-hydroxy-4-naphthalen-2-yl-3-{5-[(3-phenyl-propionylamino)-methyl]-[1,2,3]triazol-1-yl}-butyramide* (18)** *(R)-3-(5-Aminomethyl-[1,2,3]triazol-1-yl)-4-naphthalen-2-yl-butyric acid* (0.185 g, 0.60 mmol) was dissolved in dioxane (1.8 mL) and an aqueous saturated solution of K_2CO_3 (3.5 mL). The biphasic mixture was cooled to 0°C. A solution of 3-phenyl-propionyl chloride (0.089 mL, 0.60 mmol) in dioxane (1.8 mL) was slowly added to the amino acid solution and the reaction was allowed to slowly warm to room temperature. After 4 hours, the reaction was dissolved in water and extracted with diethyl ether (twice). The resulting aqueous layer was acidified to pH 1 with 3 M HCl (aq) and extracted with ethyl acetate (3 times). The combined organic layers were dried with $MgSO_4$, filtered and concentrated under reduced pressure to give (R)-4-naphthalen-2-yl-3-{5-[(3-phenyl-propionylamino)-methyl]-[1,2,3]triazol-1-yl}-butyric acid as a yellowish solid (264 mg, Quant.). Purity: 74%, LC tR= 2.59 min (method A), MS (ESI+): m/z= 443 $[M + H]^+$. (R)-4-naphthalen-2-yl-3-{5-[(3-phenyl-propionylamino)-methyl]-[1,2,3]triazol-1-yl}-butyric acid (0.264 g, 0.60 mmol) was dissolved in DMF (17 mL). EDCI (0.293 g, 2.12 mmol), HOBt (0.656 g, 2.63 mmol), N-methylmorpholine (0.92 mL, 10.59 mmol) were added. The mixture was stirred at room temperature for 5 min, and *O*-tritylhydroxylamine (0.583 g, 2.12 mmol) was added. The mixture was stirred at room temperature overnight. The solvent was evaporated under reduced pressure and the residue was dissolved in CH_2Cl_2 and washed three times with a 5% $NaHCO_3$ (aq) solution and once with water. The organic layer was dried with $MgSO_4$, filtered and concentrated under reduced. The crude product was purified by flash chromatography on silica gel (CH_2Cl_2/MeOH 10:0 to 95:5 (v/v)) to afford (R)-4-naphthalen-2-yl-3-{5-[(3-phenyl-propionylamino)-methyl]-[1,2,3]triazol-1-yl}-N-trityloxy-butyramide as a white amorphous solid (0.126 g, 21%). Purity: 72%, LC tR= 3.47 min (method A), MS (ESI+): m/z= 700 $[M + H]^+$. *O*-trityl hydroxamate intermediate was dissolved in TFA 5%/CH_2Cl_2 (1.7 mL) and triisopropylsilane (0.09 mL) was added. The mixture was stirred at room temperature for 15 min. Solvents were removed under reduced pressure and the residue was triturated with diethyl ether and petroleum ether to give a residue which was purified by flash chromatography on silica gel (CH_2Cl_2/MeOH 10:0 to 95:5 (v/v)) to afford compound **18** as a white solid (0.030 g, 3 steps 11%). Purity: 100%, LC tR= 2.45 min (method A), MS (ESI+): m/z= 458 $[M + H]^+$. ^1H NMR (CD_3CN) δ: 9.53 (s, 0.7H, CONHO$\underline{H}$), 7.82-7.72 (m, 3H), 7.46-7.43 (m, 3H),

7.29-7.10 (m, 7H), 6.94 (m, 0.9H, CONH), 5.13-5.04 (m, 1H), 3.91 (dd, J = 5.4 and 15.6 Hz, 1H), 4.15 (dd, J = 4.2 and 15.6 Hz, 1H), 3.38 (d, J = 7.5 Hz, 2H), 3.09-2.96 (m, 2H), 2.80 (t, J = 7.5 Hz, 2H), 2.27 (t, J = 7.5 Hz, 2H). ^{13}C NMR (CD_3CN) δ: 172.2, 167.0, 146.0, 141.2, 135.5, 134.6, 133.3, 132.3, 132.0, 128.3, 128.1, 127.8, 127.5 (2C), 127.5, 127.2, 126.3, 126.0, 125.9, 117.3, 57.1, 41.4, 37.9, 37.0, 31.1, 30.9. HRMS m/z calculated for $C_{26}H_{28}N_5O_3$ $(M+H)^+$ 458.2192, found 458.2209.

Synthesis of azide precursors 20a-b

To a stirred solution of the corresponding β-amino acid (11.1 mmol) in methanol (48 mL) was added dropwise thionyl chloride (12 mL) at 0 °C. After stirring at room temperature overnight, the solvent was evaporated and the crude material precipitated in ether to give the methyl ester. Imidazole-1-sulfonyl azide hydrochloride (1.2 equiv) was added to the β-amino ester (1equiv), K_2CO_3 (1 equiv) and $CuSO_4.5H_2O$ (0.01equiv) in methanol and the mixture was stirred at room temperature overnight. The mixture was concentrated, diluted with H_2O, acidified with concentrated HCl and extracted with ethyl acetate (3 times). The combined organic layers were washed with brine, dried ($MgSO_4$), filtered and concentrated. Purification by preparative liquid chromatography gave the azide (MeOH/H_2O 30/70 to 100% MeOH within 30 minutes). The ester (1equiv) was dissolved in MeOH. $HONH_2$.HCl (7.2 equiv) was dissolved in MeOH. KOH (11.4 equiv) was dissolved in MeOH. The KOH solution was poured into the $HONH_2$.HCl solution, and the resulting mixture was cooled to 0 °C for 1 h. The KOH/$HONH_2$ solution was then filtered into the solution of the ester, and the reaction mixture was stirred at room temperature until completion. After removal of the solvent, the mixture was dissolved in ethyl acetate and washed successively with a 1N solution of HCl and brine. Removal of the solvent gave a brown oil which was purified by preparative HPLC (MeOH/H_2O 30/70 to 100% MeOH within 30 minutes).

3-azido-N-hydroxy-4-phenylbutanamid (20a). Orange oil (75 mg, 25%), Purity: 100%, LC t_R= 4.04 min (method B), MS (ESI-): *m/z* =219 $(M-H)^-$, ^{1}H NMR (CD_3OD) δ: 7.38-7.24 (m, 5H), 4.11-4.02 (m, 1H), 2.91 (dd, J = 5.3 Hz and J = 13.8 Hz , 1H), 2.79 (dd, J = 8.4 Hz and J = 13.8 Hz , 1H), 2.34 (dd, J = 4.7 Hz and J = 14.5 Hz , 1H), 2.22 (dd, J = 9.1 Hz and J = 14.5 Hz , 1H). ^{13}C NMR (CD_3OD) δ: 168.2, 137.3, 129.1, 128.2, 126.5, 60.8, 40.3, 37.2.

(3R)-3-azido-N-hydroxy-4-naphthylbutanamide **(20b)** Orange oil (144 mg, 29%), Purity: 90%, LC t_R= 5.25 min (method B), MS (ESI+): *m/z* =271 $(M+H)^+$. ^{1}H NMR (CD_3OD) δ: 7.84-7.80 (m, 3H), 7.74 (sl, 1H), 7.47-7.40 (m, 3H), 4.20 (dddd, J = 4.7, 5.3, 8.2 Hz and 9.1 Hz , 1H), 3.08 (dd, J = 5.3 Hz and J = 13.7 Hz , 1H), 2.98 (dd, J = 8.2 Hz and J = 13.7 Hz , 1H), 2.38 (dd, J = 4.7 Hz and J = 14.6 Hz , 1H) 2.28 (dd, J = 9.1 Hz and J = 14.6 Hz , 1H). ^{13}C NMR (CD_3OD) δ: 168.3, 134.8, 133.6, 132.5, 127.9, 127.8, 127.3, 127.2, 125.8, 125.4, 60.7, 40.4, 37.3.

Synthesis of azide precursors 20c-e

Imidazole-1-sulfonyl azide hydrochloride (1.2 equiv) was added to the amino-acid (1equiv), K_2CO_3 (1 equiv) and $CuSO_4.5H_2O$ (0.01equiv) in methanol and the mixture was stirred at room temperature overnight. The mixture was concentrated, diluted with H_2O, acidified with concentrated HCl and extracted with ethyl acetate (3 times). The combined organic layers were washed with brine, dried ($MgSO_4$), filtered and concentrated. Purification by preparative liquid chromatography gave the azide (MeOH/H_2O 30/70 to 100% MeOH within 30 minutes).

(2R)-2-Azido-3-naphthalen-2-yl-propionic acid. **(20c)**. Brown solid (764 mg, 79%), Purity: 100%, LC t_R = 2.39 min, MS (ESI-): *m/z* = 240 $[M-H]^-$. ^{1}H NMR ($CDCl_3$) δ: 7.83-7.81 (m, 3H), 7.72 (s, 1H), 7.51-7.47 (m, 2H),

7.39-7.37 (dd, J = 1.5 and 8.4 Hz, 1H), 4.25 (dd, J = 4.8 and 8.7 Hz, 1H), 3.40 (dd, J = 4.5 and 13.8 Hz, 1H), 3.19 (dd, J = 9 and 14.1 Hz, 1H). ^{13}C NMR ($CDCl_3$) δ: 133.5, 133.1, 132.6, 128.6, 128.2, 127.8, 127.1, 126.4, 126.0, 57.0, 37.7

(R)-3-Azido-4-naphthalen-2-yl-butyric acid **(20d)**. Yellow oil (2.34 g, quant.), Purity: 72%, LC tR= 2.84 min (method A), MS (ESI+): *m/z* = 228 [M - 28 (N2) + H]$^+$. ^1H NMR ($CDCl_3$) δ: 7.85-7.80 (m, 3H), 7.69 (s, 1H), 7.52-7.44 (m, 2H), 7.37 (dd, J = 1.6 and 8.4 Hz, 1H), 4.77 (sl, 1H), 4.22-4.13 (m, 1H), 3.14-2.99 (m, 2H), 2.66-2.51 (m, 2H). ^{13}C NMR ($CDCl_3$) δ: 175.7, 134.0, 133.5, 132.5, 128.5, 128.2, 127.7, 127.6, 127.3, 126.3, 125.9, 59.8, 40.7, 38.5.

(3S)-3-Azido-4-naphthalen-2-yl-butyric acid **(20e)**. Yellow amorphous solid (902 mg, quantitative yield), Purity: 61%, LC t_R= 3.12 min (method A), MS (ESI-): *m/z* =254 (M-H)$^-$. ^1H NMR ($CDCl_3$) δ: 7.85-7.81 (m, 3H), 7.69 (s, 1H), 7.52-7.45 (m, 2H), 7.37 (dd, J = 1.8 and 8.4 Hz, 1H), 4.92 (brs, 1H), 4.22-4.13 (m, 1H), 3.11 (dd, J = 7.2 and 13.5 Hz, 1H), 7.37 (dd, J = 6.6 and 13.5 Hz, 1H), 2.61-2.57 (m, 2H). ^{13}C NMR ($CDCl_3$) δ: 176.3, 134.0, 133.5, 132.5, 128.5, 128.2, 127.7, 127.6, 127.3, 126.3, 125.9, 59.8, 40.7, 38.6.

Standard procedure A for the synthesis of N-Prop-2-ynyl-arylamide or N-Prop-2-ynyl-alkylamide and N-Prop-2-ynyl-arylsulfonamide or N-Prop-2-ynyl-alkylsulfonamide from Supplementary Table S1. Propargylamine (200 µL, 1.1 equiv) and diisopropylamine (462 µL, 1.2 equiv) were solubilized in dichloromethane (4 mL). The reaction media was cooled down to 0 °C and 4-Methyl-benzoyl chloride (353 µL, 1 equiv) or the corresponding chloride or sulfonylchloride was added dropwise. The reaction was stirred at room temperature overnight. The organic layer was washed with HCl 1N (2x4 mL), with $NaHCO_3$ 5% (2x4 mL), with water (2x4 mL) and with brine (1x4 mL), then dried over $MgSO_4$ and reduced under pressure to give the product. If necessary deprotection of Boc groups is performed using TFA/DCM (1.5/4) at room temperature during 1.5 h.

*2-Methyl-pentanoic acid prop-2-ynylamide (**5a**).* Yellow powder (231 mg, 57%), Purity: 96%. LC t_R= 3.90 min, MS (ESI+): *m/z* =154 (M+H)$^+$. ^1H NMR ($CDCl_3$) δ: 5.72 (s, 1H), 4.06 (dd, J = 2.7, and 5.4 Hz, 2H), 2.25 (m, 1H+1H), 1.70-1.55 (m, 1H), 1.43-1.24 (m, 3H), 1.14 (d, J=7.2 Hz, 3H), 0.90 (t, J= 6.9 Hz, 3H). Mp = 38.7-39.8 °C.

*3-Cyclohexyl-N-prop-2-ynyl-propionamide (**5b**).* White powder (230 mg, 45%), Purity: 89%. LC t_R= 5.11 min, MS (ESI+): *m/z* =194 (M+H)$^+$. ^1H NMR ($CDCl_3$) δ: 5.65 (s, 1H), 4.05 (dd, J = 2.7, and 5.4 Hz, 2H), 2.24 (m, 2H+1H), 1.68 (m, 5H), 1.52 (m, 2H), 1.20 (m, 4H), 0.90 (m, 2H). Mp = 71.1-71.9 °C.

*N-Prop-2-ynyl-isobutyramide (**5c**).* White powder (142 mg, 43%), LC t_R= 3.01 min , MS (ESI+): *m/z* =126 (M+H)$^+$. ^1H NMR ($CDCl_3$) δ: 5.62 (s, 1H), 4.04 (dd, J=2.5 and 5.1 Hz, 2H), 2.37 (sept, J = 6.8 Hz, 1H), 1.16 (d, J= 6.8 Hz, 6 H). Mp = 63.3-64.1 °C.

*Cyclopentanecarboxylic acid prop-2-ynylamide (**5d**).* Pale yellow powder (324 mg, 81%), Purity: 93%, LC t_R= 3.58 min, MS (ESI+): *m/z* =152 (M+H)$^+$.^1H NMR ($CDCl_3$) δ: 5.62 (s, 1H), 4.06 (dd, J = 2.7, and 5.4 Hz, 2H), 2.54 (m, 1H), 2.23 (t, J = 2.7 Hz, 1H), 1.88-1.73 (m, 8H). Mp = 103.2-103.7 °C.

*Thiophene-2-carboxylic acid prop-2-ynylamide (**5e**).* White powder (395 mg, 96%), Purity: 100%, LC t_R= 3.37 min , MS (ESI+): *m/z* =166 (M+H)$^+$. ^1H NMR ($CDCl_3$) δ: 7.59 (dd, J = 1.1 and 3.7 Hz, 1H), 7.49 (dd, J = 1.1 and 5.0 Hz, 1H), 7.09 (dd, J = 3.7, and 5.0 Hz 1H), 6.52 (sl, 1H), 4.25 (dd, J = 2.5 and 5.3 Hz, 2H), 2.28 (t, J = 2.5 Hz, 1H). Mp = 115.5-118.5 °C.

*N-Prop-2-ynyl-benzamide (**5f**).* White powder (482 mg, 92%), Purity: 100%, LC t_R= 3.64 min , MS (ESI+): *m/z* =160 $(M+H)^+$. ^1H NMR ($CDCl_3$) δ: 7.80 (m, 1H), 7.54-7.48 (m, 1H), 7.46-7.40 (m, 2H), 6.36 (sl, 1H), 4.25 (dd, *J* = 2.6 and 5.2 Hz, 2H), 2.27 (t, *J* = 2.6 Hz, 1H). Mp = 105.9-108.7 °C.

*4-Cyano-N-prop-2-ynyl-benzamide (**5g**).* White powder (193 mg, 46%), Purity: 100%, LC t_R= 3.67 min, MS (ESI+): *m/z* =185 $(M+H)^+$. ^1H NMR ($CDCl_3$) δ: 7.91 (dt, J=8.7 and 2.1 Hz, 2H), 7.77 (dt, J=8.7 and 2.1 Hz, 2H), 6.36 (s, 1H), 4.28 (dd, *J* = 2.7 and 5.1 Hz, 2H), 2.33 (t, *J* = 2.7 Hz, 1H). Mp = 179.3-180.3 °C.

*N-Prop-2-ynyl-4-trifluoromethyl-benzamide (**5h**).* White powder (180 mg, 30%), Purity: 100%, LC t_R= 4.87 min, MS (ESI+): *m/z* =228 $(M+H)^+$. ^1H NMR ($CDCl_3$) δ: 7.92 (dd, *J* = 0.6 and 8.7 Hz, 2H), 7.72 (dd, *J* = 0.6 and 8.7 Hz, 2H), 6.39 (s, 1H), 4.30 (dd, *J* = 2.7, and 5.4 Hz, 2H), 2.33 (t, *J* = 2.7 Hz, 1H). Mp = 148.9-149.4 °C.

*3-Cyclopentyl-N-prop-2-ynyl-propionamide (**5i**).* White powder (447 mg, 93%), Purity: 93%, LC t_R= 4.74 min, MS (ESI+): *m/z* =180 $(M+H)^+$.^1H NMR ($CDCl_3$) δ: 5.64 (s, 1H), 4.05 (dd, *J* = 2.7, and 5.4 Hz, 2H), 2.23 (t, *J* = 2.7 Hz, 1H), 2.22 (m, 2H), 1.82-1.46 (m, 9H), 1.13-1.06 (m, 2H). Mp = 72.7-73.4 °C.

*3-Methyl-N-prop-2-ynyl-butyramide (**5j**).* White powder (329 mg, 89%), Purity: 92%,LC t_R= 3.35 min, MS (ESI+): *m/z* =140 $(M+H)^+$. ^1H NMR ($CDCl_3$) δ: 5.67 (s, 1H), 4.05 (dd, *J* = 2.7, and 5.4 Hz, 2H), 2.23 (t, *J* = 2.7 Hz, 1H), 2.20-2.05 (m, 2H+1H), 0.96 (t, *J* = 6.3Hz, 6H). Mp = 47.8-49.0 °C.

*Pentanoic acid prop-2-ynylamide (**5k**).* White powder (317 mg, 86%), Purity: 90%, LC t_R= 3.45 min, MS (ESI+): *m/z* =140 $(M+H)^+$. ^1H NMR ($CDCl_3$) δ: 5.67 (s, 1H), 4.06 (dd, *J* = 2.7, and 5.4 Hz, 2H), 2.25-2.19 (m, 2H+1H), 1.64 (m, 2H), 1.35 (m, 2H), 0.93 (t, J=7.2Hz, 3H). Mp = 42.1-42.7 °C.

*Cyclohexanecarboxylic acid prop-2-ynylamide (**5l**).* Pale yellow powder (428 mg, 98%), Purity: 89%, LC t_R= 4.03 min, MS (ESI+): *m/z* =166 $(M+H)^+$. ^1H NMR ($CDCl_3$) δ: 5.67 (s, 1H), 4.05 (dd, *J* = 2.7, and 5.4 Hz, 2H), 2.22 (t, J= 2.7Hz, 1H), 2.10 (tt, *J* = 3.6 and 11.7 Hz, 1H), 1.90-1.76 (m, 4H), 1.68-1.64 (m, 1H), 1.50-1.18 (m, 5H). Mp = 100.6-101.3 °C.

*2-Methylsulfanyl-N-prop-2-ynyl-nicotinamide (**5m**).* Pale yellow powder (128 mg, 35%), Purity: 100%, LC t_R= 3.51 min, MS (ESI+): *m/z* =207 $(M+H)^+$.^1H NMR ($CDCl_3$) δ: 8.53 (dd, *J* = 1.8 and 4.8 Hz, 1H), 7.86 (dd, *J* = 1.8 and 7.5 Hz, 1H), 7.08 (dd, *J* = 4.8 and 7.5 Hz, 1H), 6.61 (s, 1H), 4.28 (dd, *J* = 2.7, and 5.4 Hz, 2H), 2.61 (s, 3H), 2.31 (t, *J* = 2.7 Hz, 1H). Mp = 120.0-121.0 °C.

*Furan-2-carboxylic acid prop-2-ynylamide (**5n**).* White powder (246 mg, 63%), Purity: 100%, LC t_R= 2.94 min, MS (ESI+): *m/z* =150 $(M+H)^+$. ^1H NMR ($CDCl_3$) δ: 7.46 (dd, J= 0.6 and 1.8 Hz, 1H), 7.15 (dd, J= 0.6 and 3.6 Hz, 1H), 6.55 (bs, 1H), 6.52 (dd, J= 1.8 and 3.6 Hz, 1H), 4.24 (dd, *J* = 2.7 and 5.4 Hz, 2H), 2.29 (t, *J* = 2.7 Hz, 1H). Mp = 66.6-67.3 °C

*N-Prop-2-ynyl-2-thiophen-2-yl-acetamide (**5o**).* White powder (303 mg, 64%), Purity: 100%, LC t_R= 3.62 min, MS (ESI+): *m/z* =180 $(M+H)^+$. ^1H NMR ($CDCl_3$) δ: 7.29 (m, 1H), 7.03 (m, 1H), 6.98 (m, 1H), 5.80 (s, 1H, NH), 4.05 (dd, *J* = 2.7 and 5.4 Hz, 2H), 3.82 (s, 2H), 2.22 (t, *J* = 2.7 Hz, 1H). Mp = 98.0-98.4 °C

*3-Phenyl-N-prop-2-ynyl-propionamide (**5p**).* Beige powder (371 mg, 75%), Purity: 100%, LC t_R= 4.23 min, MS (ESI+): *m/z* =188 $(M+H)^+$. ^1H NMR ($CDCl_3$) δ: 7.34-7.20 (m, 5H), 5.59 (s, 1H, NH), 4.04 (dd, *J* = 2.7 and 5.4 Hz, 2H), 2.99 (t, J= 7.5 Hz, 2H), 2.51 (t, *J* = 7.5 Hz, 2H), 2.23 (t, *J* = 2.7 Hz, 1H). Mp = 66.7-67.1 °C

*4-Methoxy-N-prop-2-ynyl-benzamide (**5q**).* White powder (403 mg, 87%), Purity: 100%, LC t_R= 3.82 min, MS (ESI+): *m/z* =190 $(M+H)^+$. ^1H NMR ($CDCl_3$) δ: 7.76 (dt, *J* = 2.1 and 9.6 Hz, 2H), 6.94 (dt, *J* = 3.0 and 9.6 Hz, 2H), 6.21 (bs, 1H), 4.27 (dd, *J*= 2.7 and 5.1 Hz, 2H), 3.87 (s, 3H), 2.30 (t, *J*=2.7 Hz, 1H). Mp = 132.9-133.6 °C.

*4-Fluoro-N-prop-2-ynyl-benzamide (**5r**).* White powder (592 mg, 100%), Purity: 100%, LC t_R= 3.90 min, MS (ESI+): *m/z* =178 $(M+H)^+$. ^1H NMR ($CDCl_3$) δ: 7.82 (dd, J=8.9 and 5.3 Hz, 2H), 7.14 (t, J=8.9Hz, 2H), 6.24 (s, 1H), 4.26 (dd, *J* = 2.4 and 5.1 Hz, 2H), 2.31 (t, *J* = 2.4 Hz, 1H). Mp = 144.8-145.4 °C.

3,5,5-Trimethyl-hexanoic acid prop-2-ynylamide (**5s**). Yellow oil (368 mg, 71%), Purity: 90%, LC t_R= 5.77 min, MS (ESI+): *m/z* =196 $(M+H)^+$. ^{1}H NMR ($CDCl_3$) δ: 5.59 (s, 1H), 4.04 (dd, *J* = 2.5 and 5.2 Hz, 2H), 2.21 (t, *J* = 2.6 Hz, 1H), 2.19 (dd, *J* = 5.5 and 13.1 Hz, 1H), 2.01-2.11 (m, 1H), 1.95 (dd, *J* = 8.1 and 13.1 Hz, 1H), 1.23 (dd, *J* = 3.7 and 14.0 Hz, 1 H), 1.10 (dd, *J* = 6.4 and 14.0 Hz, 1 H), 0.97 (d , *J* = 6.4 Hz, 3 H), 0.90 (s, 9 H).

*(1R,2R)-2-Phenyl-cyclopropanecarboxylic acid prop-2-ynylamide (**5t**).* white powder (332 mg, 63%), Purity: 97%, LC t_R= 4.61 min, MS (ESI+): *m/z* =200 (M+H)+. ^{1}H NMR ($CDCl_3$) δ: 7.29 (m, 3H), 7.10 (m, 2H), 5.90 (s, 1H), 4.11 (dd, *J* = 2.7, and 5.4 Hz, 2H), 2.52 (m, 1H), 2.26 (t, *J* = 2.7 Hz, 1H), 1.69-1.60 (m, 3H), 1.32-1.25 (m, 1H). Mp = 11.4-112.0 °C.

*2-Cyclohexyl-N-prop-2-ynyl-acetamide (**5u**).* White powder (196 mg, 41%), Purity: 96%, LC t_R= 5.00 min, MS (ESI+): *m/z* =180 $(M+H)^+$. ^{1}H NMR ($CDCl_3$) δ: 5.54 (s, 1H), 4.05 (dd, *J* = 2.5 and 5.2 Hz, 2H), 2.21 (t, *J* = 2.6 Hz, 1 H), 2.04 (d, *J* = 6.9 Hz, 2H), 1.85-1.60 (m, 5H+1H), 1.33-1.04 (m, 3H), 0.98-0.85 (m, 2H). Mp = 103.5-104.1 °C.

*2-Methyl-N-prop-2-ynyl-butyramide (**5v**)* Pale yellow powder (192 mg, 52%), Purity: 89%, LC t_R= 3.24 min, MS (ESI+): *m/z* =140 $(M+H)^+$. ^{1}H NMR ($CDCl_3$) δ: 5.67 (s, 1H), 4.06 (dd, *J* = 2.7, and 5.1 Hz, 2H), 2.23 (t, *J* = 2.7 Hz, 1H), 2.12 (m, 1H), 1.68 (m, 1H), 1.45 (m, 1H), 1.14 (d, J= 6.9 Hz, 3H), 0.91 (t, *J* = 7.5 Hz, 3H). Mp = 39.4-40.0 °C.

*Adamantane-1-carboxylic acid prop-2-ynylamide (**5w**).* White powder (433 mg, 75%), Purity: 91%, LC t_R= 5.62 min, MS (ESI+): *m/z* =218 $(M+H)^+$. ^{1}H NMR ($CDCl_3$) δ: 5.72 (s, 1H), 4.03 (dd, *J* = 2.6 and 5.0 Hz, 2H), 2.22 (t, *J* = 2.6 Hz, 1 H), 2.05 (m, 3H), 1.85 (m, 6H), 1.79-1.66 (m, 6H). Mp = 124.6-125.3 °C.

*2-Phenyl-N-prop-2-ynyl-butyramide (**5x**).* Yellow powder (360 mg, 68%), Purity: 100%, LC t_R= 4.73 min, MS (ESI+): *m/z* =202 $(M+H)^+$. ^{1}H NMR ($CDCl_3$) δ: 7.38-7.26 (m, 5H), 5.67 (s, 1H), 4.06 (ddd, *J* = 2.7, 5.4 and 17.7 Hz, 1H), 3.94 (ddd, *J* = 2.7, 5.1 and 17.7 Hz, 1H), 3.26 (t, *J* = 7.5 Hz, 1H), 2.29-2.14 (m, 1H+1H), 1.89-1.74 (m, 1H), 0.89 (t, *J* = 7.5 Hz, 3H). Mp = 49.6-50.0 °C.

*4-Methyl-N-prop-2-ynyl-benzamide (**5y**).* White powder (395 mg, 86%), Purity: 100%, LC t_R= 4.17 min, MS (ESI+): *m/z* =174 $(M+H)^+$. ^{1}H NMR ($CDCl_3$) δ: 7.70 (dt, J= 2.1 and 8.1 Hz, 2H), 7.24 (d, *J* = 7.8 Hz, 2H), 6.39 (bs, 1H), 4.26 (m, 2H), 2.41 (s, 3H), 2.28 (m, 1H). Mp = 116.8-117.2 °C.

*6-Chloro-N-prop-2-ynyl-nicotinamide (**5z**).* White powder (186 mg, 36%), Purity: 100%, LC t_R= 3.48 min, MS (ESI+): *m/z* =195 $(M+H)^+$. ^{1}H NMR ($CDCl_3$) δ: 8.78 (dd, *J* = 0.6 and 2.4 Hz, 1H), 8.12 (dd, *J* = 2.4 and 8.4 Hz, 1H), 7.45 (dd, *J* = 0.6 and 8.4 Hz, 1H), 6.39 (s, 1H), 4.28 (dd, *J* = 2.7 and 5.4 Hz, 2H), 2.34 (t, *J* = 2.7 Hz, 1H). Mp = 140.9-142.3 °C.

*2-Ethyl-N-prop-2-ynyl-butyramide (**5aa**).* Pale yellow powder (326 mg, 80%), Purity: 92%, LC t_R= 3.73 min, MS (ESI+): *m/z* =154 $(M+H)^+$. ^{1}H NMR ($CDCl_3$) δ: 5.78 (s, 1H), 4.08 (dd, *J* = 2.7, and 5.4 Hz, 2H), 2.23 (m, 1H), 1.89 (m, 1H), 1.70-1.41 (m, 4H), 0.90 (t, J= 7.5 Hz, 6H). Mp = 65.0-66.6 °C.

*((S)-5-tert-Butoxycarbonylamino-1-prop-2-ynylcarbamoyl-pentyl)-carbamic acid tert-butyl ester (**5ab**).* Colorless oil (694 mg, 63%). LC t_R= 5.11 min, MS (ESI+): *m/z* =384 $(M+H)^+$. ^{1}H NMR ($CDCl_3$) δ: 6.66 (sl, 1H), 5.18 (sl, 1H), 4.65 (sl, 1H), 4.11-4.06 (m, 1H), 4.04 (dd, *J* = 2.5 and 5.3 Hz, 1H), 3.11 (dd, *J* = 6.0 and 12.4 Hz, 2H), 2.22 (t, *J* = 2.5 Hz, 1H), 1.90-1.78 (m, 2H), 1.69-1.60 (m, 1H), 1.52-1.32 (m, 4H), 1.44 (s, 18H).

*3,5-Dimethyl-isoxazole-4-carboxylic acid prop-2-ynylamide (**5ac**).* Pale yellow powder (227 mg, 49%), Purity: 100%, LC t_R= 3.51 min, MS (ESI+): *m/z* =179 $(M+H)^+$.^{1}H NMR ($CDCl_3$) δ: 5.74 (s, 1H), 4.20 (dd, *J* = 2.7, and 5.4 Hz, 2H), 2.64 (s, 3H), 2.45 (s, 3H), 2.30 (t, *J* = 2.7 Hz, 1H). Mp = 101.2-102.3 °C

*(S)-2,6-Diamino-hexanoic acid prop-2-ynylamide (**5ad**).* Yellow oil (284 mg, quant.). LC t_R= 0.69 min , MS (ESI+): *m/z* =184 $(M+H)^+$. ^{1}H NMR ($CDCl_3$) δ: 4.04 (dd, *J* = 2.5 Hz, 1H), 3.85 (t, *J* = 6.5 Hz, 1H), 2.93 (t, *J* = 7.6 Hz, 2H), 2.66 (t, *J* = 2.5 Hz, 1 H), 1.93-1.84 (m, 2H), 1.76-1.65 (m, 2H), 1.53-1.45 (m, 2H).

*N-But-3-ynyl-4-fluoro-benzamide (**5ae**).* White solid (627 mg, 94%), Purity : 100%, LC t_R = 2.11 min, MS (ESI+): *m/z* = 192 $[M+H]^+$. ^{1}H NMR ($CDCl_3$) δ: 7.82-7.78 (m, 2H), 7.14-7.08 (m, 2H), 6.57 (bs, 1H), 3.60 (q, *J* = 6.3 Hz, 2H), 2.55-2.50 (m, 2H), 2.06 (t, *J* = 2.7 Hz, 1H)

*Cyclopropanesulfonic acid prop-2-ynylamide (**6a**).* Yellow oil (172 mg, 76%). LC t_R= 2.82min , MS (ESI+): *m/z* =160 $(M+H)^+$. ^{1}H NMR ($CDCl_3$) δ: 4.65 (sl, 1H), 3.97 (dd, *J* = 2.5 and 6.2 Hz, 2H), 2.56 (dddd, *J* = 4.9, 7.9, 9.8 and 12.8 Hz, 1H), 2.35 (t, *J* = 2.5 Hz, 1H), 1.26-1.20 (m,2H), 1.08-1.01 (m, 2H).

*Propane-2-sulfonic acid prop-2-ynylamide (**6b**).* Yellow oil (162 mg, 59%), LC t_R= 3.56 min, MS (ESI+): *m/z* =162 $(M+H)^+$. ^{1}H NMR ($CDCl_3$) δ: 4.36 (s, 1H), 3.96 (dd, *J* = 2.5 and 6.1 Hz, 2H), 3.29 (sept, *J* = 6.8 Hz, 1H), 2.34 (t, *J* = 2.5 Hz, 1H), 1.41 (d, J=6.8 Hz, 6H).

*2-Methyl-propane-1-sulfonic acid prop-2-ynylamide (**6c**).* Yellow oil (488 mg, 44%). LC t_R= 4.47 min, MS (ESI+): *m/z* =176 $(M+H)^+$. ^{1}H NMR ($CDCl_3$) δ: 4.47 (s, 1H), 3.95 (dd, *J* = 2.4 and 6.1 Hz, 2H), 3.05 (d, *J* = 6.4 Hz, 2H), 2.35 (t, *J* = 2.5 Hz, 1H), 2.30 (m, 1H), 1.12 (d, *J* = 6.8 Hz, 6H).

*Benzo[1,2,5]oxadiazole-4-sulfonic acid prop-2-ynylamide (**6d**).* Brown powder (490 mg, 78%), Purity: 100%, LC t_R= 4.57 min, MS (ESI+): *m/z* =238 $(M+H)^+$. ^{1}H NMR ($CDCl_3$) δ: 8.09 (dd, *J* = 0.7 and 9.0 Hz, 1H), 8.05 (dd, *J* = 0.7 and 6.7 Hz, 1H), 7.55 (dd, *J* = 6.7 and 9.0 Hz, 1H), 5.35 (m, 1H), 3.98 (dd, *J* = 6.3 and 2.5 Hz, 2H), 1.77 (t, *J* = 2.5 Hz, 1H). Mp = 107.9-109.9 °C

*Thiophene-2-sulfonic acid prop-2-ynylamide (**6e**).* white powder (330 mg, 66%), Purity: 100%. LC t_R= 3.98 min , MS (ESI+): *m/z* =202 $(M+H)^+$. ^{1}H NMR ($CDCl_3$) δ: 7.66 (dd, *J* = 1.4 and 3.8 Hz, 1H), 7.62 (dd, *J* = 1.4 and 5.0 Hz, 1H), 7.10 (dd, *J* = 3.8, and 5.0 Hz 1H), 4.83 (sl, 1H), 3.90 (dd, *J* = 2.6 and 6.0 Hz, 2H), 2.14 (t, *J* = 2.6 Hz, 1H). Mp = 76.3-77.3 °C.

*N-Prop-2-ynyl-benzenesulfonamide (**6f**).* Yellow oil (660 mg, quant.), Purity: 100%. LC t_R= 4.14 min , MS (ESI+): *m/z* =196 $(M+H)^+$. ^{1}H NMR ($CDCl_3$) δ: 7.91 (ddd, *J* = 1.4, 5.2 and 7.4 Hz, 2H), 7.64-7.58 (m, 1H), 7.53-7.48 (m, 1H), 7.56-7.50 (m, 2H), 4.83 (sl, 1H), 3.86 (dd, *J* = 2.5 and 6.1 Hz, 2H), 2.08 (t, *J* = 2.5 Hz, 1H).

*4-Fluoro-N-prop-2-ynyl-benzenesulfonamide (**6g**).* White powder (372 mg, 66%), Purity: 100%. LC t_R= 4.83 min, MS (ESI+): *m/z* =213 $(M+H)^+$. ^{1}H NMR ($CDCl_3$) δ: 7.93 (m, 2H), 7.20 (m, 2H), 4.61 (s, 1H), 3.88 (dd, *J* = 2.7 and 6.3 Hz, 2H), 2.10 (t, *J* = 2.7 Hz, 1H). Mp = 84.5-85.1 °C.

*N-Prop-2-ynyl-C-(4-trifluoromethyl-phenyl)-methanesulfonamide (**6h**).* White powder (207 mg, 90%), Purity: 100%. LC t_R= 5.73 min, MS (ESI+): *m/z* =278 $(M+H)^+$. ^{1}H NMR ($CDCl_3$) δ: 7.67 (d, *J* = 8.4 Hz, 2H), 7.60 (d, *J* = 8.4 Hz, 2H), 4.50 (t, J=5.7 Hz, 1H), 4.43 (s, 2H), 3.94 (dd, *J* = 2.4 and 6.0 Hz, 2H), 2.45 (t, *J* = 2.4 Hz, 1H). Mp = 124.8-125.3 °C.

*C-Methanesulfonyl-N-prop-2-ynyl-methanesulfonamide (**6i**).* White powder (76 mg, 13%) ^{1}H NMR (MeOD) δ: 4.45 (s, 2H), 3.80 (d, *J* = 2.6 Hz, 2H), 3.19 (s, 3H), 3.10 (t, *J* = 2.6 Hz, 1H). Mp = 156.4-164.7 °C.

*4-Oxazol-5-yl-N-prop-2-ynyl-benzenesulfonamide (**6j**).* Pale yellow powder (97 mg, 14%), Purity: 99%. LC t_R= 4.55 min, MS (ESI+): *m/z* =263 $(M+H)^+$.^{1}H NMR ($CDCl_3$) δ: 7.99 (s, 1H), 7.95 (dt, *J* = 1.8 and 8.7 Hz, 2H), 7.80 (dt, *J* = 1.8 and 8.7 Hz, 2H), 7.51 (s, 1H), 4.69 (t, *J* = 6.0 Hz, 1H), 3.90 (dd, *J* = 6.1 and 2.5 Hz, 2H), 2.09 (t, *J* = 2.5 Hz, 1H). Mp = 153.7-156.6 °C.

*3,3,3-Trifluoro-propane-1-sulfonic acid prop-2-ynylamide (**6k**).* White powder (304 mg, 69%). LC t_R= 4.56 min, MS (ESI+): *m/z* =216 $(M+H)^+$. ^{1}H NMR ($CDCl_3$) δ: 4.69 (s, 1H), 4.00 (dd, *J* = 2.5 and 6.1 Hz, 2H), 3.40 (ddd, *J* = 0.5, 4.5 and 8.1 Hz, 2H), 2.76-2.60 (m, 2H), 2.42 (t, *J* = 2.5 Hz, 1H). Mp = 40.0-40.8 °C.

*Cyclohexanesulfonic acid prop-2-ynylamide (**6l**).* Yellow oil (466 mg, 87%) LC t_R= 4.92 min, MS (ESI+): *m/z* =202 $(M+H)^+$. ^1H NMR ($CDCl_3$) δ: 4.34 (m, 1H), 3.94 (dd, *J* = 2.6 and 6.2 Hz, 2H), 3.02 (tt, *J* = 3.4 and 12.0 Hz, 1H), 2.33 (t, *J* = 2.5 Hz, 1H), 2.22 (m, 2H), 1.93-1.88 (m, 2H), 1.75-1.66 (m, 1H), 1.52-1.47 (m, 1H), 1.35-1.16 (m, 4H).

*4-Difluoromethoxy-N-prop-2-ynyl-benzenesulfonamide (**6m**).* White powder (705 mg, 100%), Purity: 100%. LC t_R= 5.28 min, MS (ESI+): *m/z* =262 $(M+H)^+$. ^1H NMR ($CDCl_3$) δ: 7.91 (ddd, *J* = 2.0, 2.5 and 8.7 Hz, 2H), 7.24 (d, *J* = 8.7 Hz, 2H), 6.60 (t, *J* = 72.5 Hz, 1H), 4.63 (t, J=6.5 Hz, 1H), 3.87 (dd, *J* = 6.1 and 2.5 Hz, 2H), 2.09 (t, *J* = 2.5 Hz, 1H). Mp = 78.5-79.8 °C.

*4-Propyl-N-prop-2-ynyl-benzenesulfonamide (**6n**).* Pale yellow powder (493 mg, 78%), Purity: 100%. LC t_R= 5.97 min, MS (ESI+): *m/z* =238 $(M+H)^+$.^1H NMR ($CDCl_3$) δ: 7.80 (dt, *J* = 8.4 and 1.8 Hz, 2H), 7.32 (d, *J* = 8.4 Hz, 2H), 4.57 (t, *J* = 5.7 Hz, 1H), 3.84 (dd, *J* = 2.4 and 6.1 Hz, 2H), 2.67 (t, *J* = 7.5 Hz, 2H), 2.09 (t, *J* = 2.7 Hz, 1H), 1.68 (sext, *J* = 7.5 Hz, 2H), 0.99 (t, J=7.5 Hz, 3H). Mp = 55.4-56.0 °C.

*Benzo[b]thiophene-2-sulfonic acid prop-2-ynylamide (**6o**).* Yellow powder (255 mg, 55%), Purity: 90%. LC t_R= 5.62 min, MS (ESI+): *m/z* =252 $(M+H)^+$. ^1H NMR ($CDCl_3$) δ: 7.93-7.87 (m, 3H), 7.54-7.44 (m, 2H), 4.84 (s, 1H), 3.97 (s, 2H), 2.10 (t, *J* = 2.7 Hz, 1H). Mp = 60.2-61.7 °C.

*Biphenyl-4-sulfonic acid prop-2-ynylamide (**6p**).* White powder (500 mg, 74%), Purity: 100%. LC t_R= 5.47 min , MS (ESI+): *m/z* =272 $(M+H)^+$. ^1H NMR ($CDCl_3$) δ: 7.98 (dd, *J* = 1.8 and 8.6 Hz, 2H), 7.75 (dd, *J* = 1.8, and 8.6 Hz, 2H), 7.63 (dd, *J* = 1.6, and 8.4 Hz 2H), 7.53-7.42 (m, 3H), 4.80 (t, *J* = 6.0 Hz, 1H), 3.91 (dd, *J* = 2.5 and 6.0 Hz, 2H), 2.13 (t, *J* = 2.5 Hz, 1H). Mp = 130.6-133.9 °C.

*4-Butoxy-N-prop-2-ynyl-benzenesulfonamide (**6q**).* Pale yellow powder (307 mg, 55%), Purity: 100%. LC t_R= 6.20 min, MS (ESI+): *m/z* =238 $(M+H)^+$. ^1H NMR ($CDCl_3$) δ: 7.81 (dt, *J* = 9.0 and 2.0 Hz, 2H), 6.97 (dt, *J* = 9.0 and 2.0 Hz, 2H), 4.60 (t, *J* = 6.0 Hz, 1H), 4.03 (t, J= 6.5 Hz, 2H), 3.82 (dd, *J* = 6.0 and 2.5 Hz, 2H), 2.12 (t, *J* = 2.5 Hz, 1H), 1.79 (m, 2H), 1.48 (m, 2H), 0.99 (t, J=7.4 Hz, 3H). Mp = 70.5-70.7 °C.

*N-Prop-2-ynyl-4-trifluoromethyl-benzenesulfonamide (**6r**).* White powder (319 mg, 46%), Purity: 100%. LC t_R= 5.76 min, MS (ESI+): *m/z* =264 $(M+H)^+$. ^1H NMR ($CDCl_3$) δ: 8.04 (d, *J* = 8.4 Hz, 2H), 7.80 (d, *J* = 8.4 Hz, 2H), 4.75 (s, 1H), 3.92 (dd, *J* = 2.4 and 6.0 Hz, 2H), 2.09 (t, *J* = 2.4 Hz, 1H). Mp = 134.5-136.2 °C.

*C-(7,7-Dimethyl-2-oxo-bicyclo[2.2.1]hept-1-yl)-N-prop-2-ynyl-methanesulfonamide (**6s**).* White powder (443 mg, 62%). LC t_R= 5.11 min, MS (ESI+): *m/z* =270 $(M+H)^+$. ^1H NMR ($CDCl_3$) δ: 6.10 (s, 1H), 4.09 (ddd; *J* = 2.5, 8.6 and J_{AB} = 18.2 Hz, 1H), 3.93 (ddd, *J* = 2.5, 3.9 and J_{AB} =18.2 Hz, 1H), 3.73 (d, J_{AB} = 15.2 Hz, 1 H), 3.04 (d, J_{AB} =15.2 Hz, 1H), 2.43 (ddd, *J* = 2.3, 4.8 and 18.6 Hz, 1H), 2.31 (t, J=2.5 Hz, 1H), 2.15-1.90 (m, 5H), 1.50-1.42 (m, 1H), 1.00 (s, 3H), 0.90 (s, 3H). Mp = 88.6-94.7 °C.

*4-(2-Oxo-pyrrolidin-1-yl)-N-prop-2-ynyl-benzenesulfonamide (**6t**).* White powder (327 mg, 82%), Purity: 100%. LC t_R= 4.37 min, MS (ESI+): *m/z* =279 $(M+H)^+$.^1H NMR ($CDCl_3$) δ: 8.05 (t, *J* = 5.7 Hz, 1H), 7.86 (dt, *J* = 9.0 and 2.4 Hz, 2H), 7.77 (dt, *J* = 9.0 and 2.4 Hz, 2H), 3.86 (t, *J* = 6.9 Hz, 2H), 3.65 (dd, *J* = 5.7 and 2.4 Hz, 2H), 3.03 (t, *J* = 2.4 Hz, 1H), 2.53 (t, *J* = 7.8 Hz, 2H), 2.07 (m, 2H). Mp = 180.8-181.6 °C.

*5-Phenyl-thiophene-2-sulfonic acid prop-2-ynylamide (**6u**).* White powder (515 mg, 70%), Purity: 100%. LC t_R= 6.10 min, MS (ESI+): *m/z* =278 $(M+H)^+$. ^1H NMR ($CDCl_3$) δ: 7.63-7.60 (m, 3H), 7.47-7.39 (m, 2H), 7.27 (s, 2H), 4.70 (t, *J* = 6.3 Hz, 1H), 3.95 (dd, *J* = 2.7 and 6.3 Hz, 2H), 2.18 (t, *J* = 2.7 Hz, 1H). Mp = 80.5-80.9 °C.

*4-Phenoxy-N-prop-2-ynyl-benzenesulfonamide (**6v**).* Yellow powder (517 mg, 77%), Purity: 100%. LC t_R= 6.08 min, MS (ESI+): *m/z* =288 $(M+H)^+$. ^1H NMR ($CDCl_3$) δ: 7.85 (dt, *J* = 8.8 and 2.8 Hz, 2H), 7.42 (m, 2H), 7.23 (m, 1H), 7.07 (m, 4H), 4.67 (t, *J* = 6.0 Hz, 1H), 3.85 (dd, *J* = 6.0 and 2.5 Hz, 2H), 2.14 (t, *J* = 2.5 Hz, 1H). Mp = 83.4-84.0 °C.

*4-Prop-2-ynylsulfamoyl-N-(2,2,2-trifluoro-ethyl)-benzamide (**6w**).* White powder (438 mg, 93%), Purity: 100%. LC t_R= 4.78 min, MS (ESI+): *m/z* =321 $(M+H)^+$. ^1H NMR (MeOD) δ: 8.00 (s, 4H), 4.15 (d, J_{AB} = 9.3 Hz, 1H), 4.09 (d, J_{AB} = 9.3 Hz, 1H), 3.81 (d, *J* = 2.5 Hz, 2H), 2.44 (t, *J* = 2.5 Hz, 1H). Mp = 186.1-188.4 °C.

*4'-Fluoro-biphenyl-4-sulfonic acid prop-2-ynylamide (**6x**).* Beige powder (613 mg, 86%), Purity: 98%. LC t_R= 6.12 min, MS (ESI-): *m/z* =288 $(M-H)^-$. ^1H NMR ($CDCl_3$) δ: 7.96 (dt, *J* = 8.5 and 2.0 Hz, 2H), 7.69 (dt, *J* = 8.5 and 2.0 Hz, 2H), 7.59 (dd, *J* = 8.7 and 5.1 Hz, 2H), 7.18 (t, *J* = 8.7 Hz, 2H), 4.69 (t, *J* = 6.0 Hz, 1H), 3.90 (dd, *J* = 6.0 and 2.5 Hz, 2H), 2.12 (t, *J* = 2.5 Hz, 1H). Mp = 154.7-155.6 °C.

*4-(2-Methyl-thiazol-4-yl)-N-prop-2-ynyl-benzenesulfonamide (**6y**).* White powder (446 mg, 58%), Purity: 100%. LC t_R= 5.30 min, MS (ESI+): *m/z* =293 $(M+H)^+$.^1H NMR ($CDCl_3$) δ: 8.02 (d, *J* = 8.7 Hz, 2H), 7.90 (d, *J* = 8.7 Hz, 2H), 7.48 (s, 1H), 4.78 (t, *J* = 5.9 Hz, 1H), 3.87 (dd, *J* = 6.1 and 2.5 Hz, 2H), 2.79 (s, 3H), 2.09 (t, *J* = 2.5 Hz, 1H). Mp = 169.1-169.6 °C.

Supplementary Scheme S1: Synthesis of alkyne reagents **5a-ad**, **6a-y**.[a]

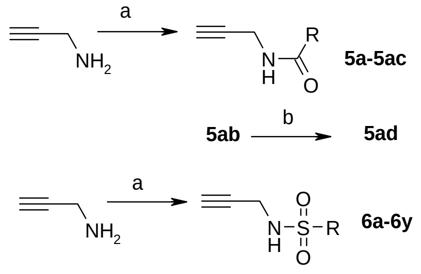

[a] *Reagents and conditions :* (a) ClCOR or $ClSO_2R$, DIEA, CH_2Cl_2, rt, overnight; (b) TFA/CH_2Cl_2 (1.5/4), room temp., 1.5 h, quant.

Supplementary Scheme S2. Synthesis of 1,5-triazoles 16-18.[a]

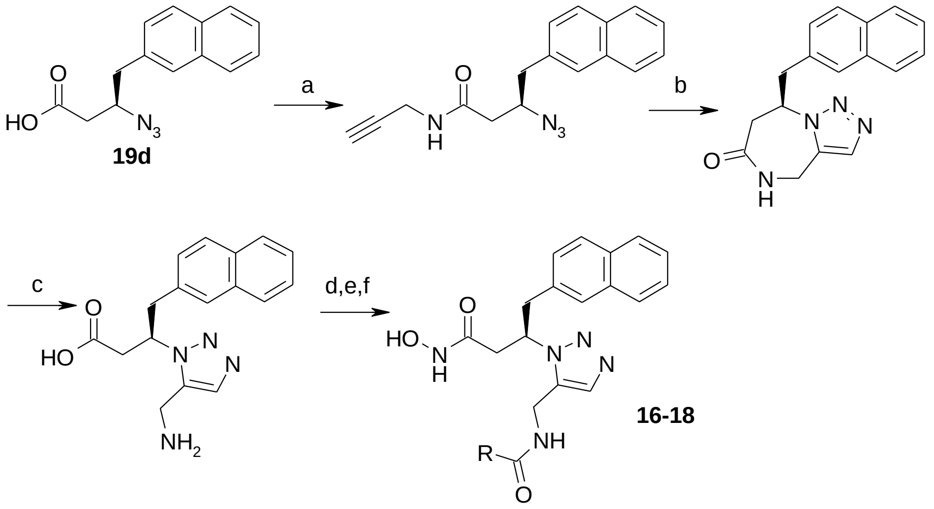

[a] *Reagents and conditions :* (a) propargylamine, DMTMM, EtOAc, room temp., 3 h, 60% ; (b) DMF, reflux, 24 h, 84%; (c) HCl 6 M, H_2O, MW, 85 °C, 1 h, quantitative yield; (d) RCOCl, dioxane, sat. K_2CO_3, 0 °C to rt, 3–5 h; (e) *O*-tritylhydroxylamine, EDCI, HOBt, N-methylmorpholine, DMF, room temp., overnight; (f) TFA, TIS, CH_2Cl_2, rt, 2 h.

The introduction of the 1,5-disubstituted pattern by a ring constrained Huisgen cycloaddition. [4] Azido compound was coupled to propargylamine with 4-(4,6-dimethoxy[1,3,5]triazin-2-yl)-4-methylmorpholinium chloride (DMTMM) to produce the corresponding alkyne, which could give efficiently the bicyclic triazole by intramolecular thermal cycloaddition. A microwave-assisted lactam hydrolysis under acidic conditions yields 1,5-disubstituted triazole intermediate. Subsequent acylations followed by the introduction of the hydroxamate group via coupling with *O*-tritylhydroxylamine gave the desired triazoles **16-18** after three steps.[5,6]

Supplementary References

[1] Qiu, W. Q. et al. Insulin-degrading enzyme regulates extracellular levels of amyloid beta-protein by degradation. *J. Biol. Chem*. **273**, 32730-8 (1998).

[2] Edbauer, D., Willem, M., Lammich, S., Steiner, H. & Haass, C. Insulin-degrading Enzyme Rapidly Removes the β-Amyloid Precursor Protein Intracellular Domain (AICD). *J. Biol. Chem.* **277**, 13389-13393 (2002).

[3] Farris, W. et al. Insulin-degrading enzyme regulates the levels of insulin, amyloid beta-protein, and the beta-amyloid precursor protein intracellular domain in vivo. *Proc. Natl. Acad. Sci. U. S. A.* **100**, 4162-4167 (2003).

[4] (a) Buysse, K. et al. Amino Triazolo Diazepines (Ata) as Constrained Histidine Mimics. *Org. Lett.* **13**, 6468 (2011). (b) Balducci, E., Bellucci, L., Petricci, E., Taddei, M. & Tafi, A. Microwave-Assisted Intramolecular Huisgen Cycloaddition of Azido Alkynes Derived from α-Amino Acids. *J. Org. Chem.* **74**, 1314 (2009).

[5] Pokorski, J. K. et al. Introduction of a Triazole Amino Acid into a Peptoid Oligomer Induces Turn Formation in Aqueous Solution. *Org. Lett.* **9**, 2381 (2007).

[6] Flipo, M. et al. Novel Selective Inhibitors of the Zinc Plasmodial Aminopeptidase PfA-M1 as Potential Antimalarial Agents. *J. Med. Chem.* **50**, 1322-1334 (2007).
